# Investigation of Host–Guest Interactions in 2-Ureido-4-ferrocenylpyrimidine Derivatives

**DOI:** 10.3390/ijms252413552

**Published:** 2024-12-18

**Authors:** Márk Váradi, Soma J. Keszei, Ágnes Gömöry, Margit Kovács, Tamás Kégl, Lajos Fodor, Rita Skoda-Földes

**Affiliations:** 1 Research Group of Organic Synthesis and Catalysis, University of Pannonia, Egyetem u. 10, 8200 Veszprém, Hungary; varadi.mark@mk.uni-pannon.hu; 2 Centre for Energy Research, Institute of Technical Physics and Materials Science, Konkoly-Thege út 29-33, 1121 Budapest, Hungary; keszei.soma@ek.hun-ren.hu; 3 MS Proteomics Research Group, Hungarian Research Network, Research Centre for Natural Sciences, Magyar tudósok körútja 2, 1117 Budapest, Hungary; gomory.agnes@ttk.hu; 4 NMR Laboratory, University of Pannonia, Egyetem u. 10, 8200 Veszprém, Hungary; kovacs.margit@mk.uni-pannon.hu; 5 Department of General and Inorganic Chemistry, University of Pécs, Ifjúság útja 6, 7624 Pécs, Hungary; tkegl@gamma.ttk.pte.hu; 6 János Szentágothai Research Center, Ifjúság útja 34, 7624 Pécs, Hungary; 7 HUN-REN-PTE Research Group for Selective Chemical Syntheses, Ifjúság útja 6, 7624 Pécs, Hungary; 8 Research Group of Environmental and Inorganic Photochemistry, University of Pannonia, Egyetem u. 10, 8200 Veszprém, Hungary; fodor.lajos@mk.uni-pannon.hu

**Keywords:** hydrogen bonds, NMR spectroscopy, conformational equilibrium, cyclic voltammetry, electrode modification

## Abstract

In the present study, synthesis, conformational behavior, host–guest complex formation, and electrochemical properties of novel 6-substituted-2-ureido-4-ferrocenylpyrimidines were explored. A comprehensive NMR spectroscopic investigation was carried out to confirm the structure and conformational equilibrium of the ureidopyrimidines through studying the temperature- and concentration dependence of NMR spectra. Low-temperature NMR measurements were used to clarify structural changes inflicted by a 2,6-diaminopyridine guest. Association constant (K_assoc_) values of host–guest complexes were calculated based on low-temperature titrations. It was shown that the introduction of a pyridin-2-yl substituent in the pyrimidine ring in host **10** induced a considerable change not only in the conformational equilibrium of the host itself but also in that of the host–guest complex. Geometries and relative stabilities of the conformers of host **10** as well as its host–guest complexes were determined by quantum chemical calculations. Electrochemical behavior of ureidopyrimidine hosts and host–guest complexes was investigated by cyclic voltammetry (CV) and linear sweep voltammetry (LSV) measurements. Two ureidopyrimidine derivatives were immobilized on the surface of spectral graphite electrodes, and their electrochemical response on the addition of 2,6-diaminopyridine was compared. These results also supported the importance of the pyridin-2-yl substituent in the efficient sensing of the guest.

## 1. Introduction

Due to its good stability, reversible redox properties [1], and easy chemical modification of the cyclopentadienyl rings [2], ferrocene (Fc) has found wide application in diverse fields of chemistry, medicine, and materials science [3]. Fc-derivatives can be used as ligands, electrocatalysts [4], redox markers, or as constituents in redox-responsive polymer gels [5] or molecular machines [6]. Fc-modified electrodes have been developed for the electrochemical detection of heavy metals, pesticides, and other pollutants [7].

Ferrocene derivatives can be excellent building blocks in sensors where the ferrocene moiety serves as a mediator [8] or can form host–guest complexes with ions or neutral molecules via a properly designed side chain [9]. Different organic moieties attached to the substituents on the cyclopentadienyl rings via non-covalent interactions may induce a shift in the redox potential of the ferrocene-ferrocenium redox couple [10]. These changes can be followed by electrochemical methods, which makes it possible to detect various analytes. While the properties of a wide range of ferrocene-based cation [11]- or anion sensors [12] have been investigated in detail, less attention has been turned towards complexation with neutral guests [9].

Among non-covalent interactions, hydrogen bonds play a pivotal role in the formation of host–guest complexes [13]. The stability of an adduct held together by hydrogen bonds greatly depends on the acidity of the donor and the basicity of the acceptor atom. Another important factor is the number of participating hydrogen bonds: the greater their number, the stronger the association in the adduct [14]. At the same time, the pattern of hydrogen bond donor and acceptor groups can also have a considerable effect on the association constants of intermolecular adducts [15,16,17]. Among H-bonding arrays involving heterocycles, ureidopyrimidinones introduced by Meijer [18] were the most successful derivatives that have been used to construct supramolecular polymers, organocatalysts, sensors, etc. [19]. The introduction of an Fc moiety into similar compounds made it possible to follow the dimerization process of ureidopyrimidinones not only by NMR and UV/Vis spectroscopy but also by electrochemical methods [20,21,22,23]. In addition to ureido-pyrimidinones, ureido-heterocycles with triple H-bonding arrays or offering different bonding patterns can also be useful in molecular recognition, as no considerable competition with homodimerization should be reckoned with [19].

**Figure 1:**
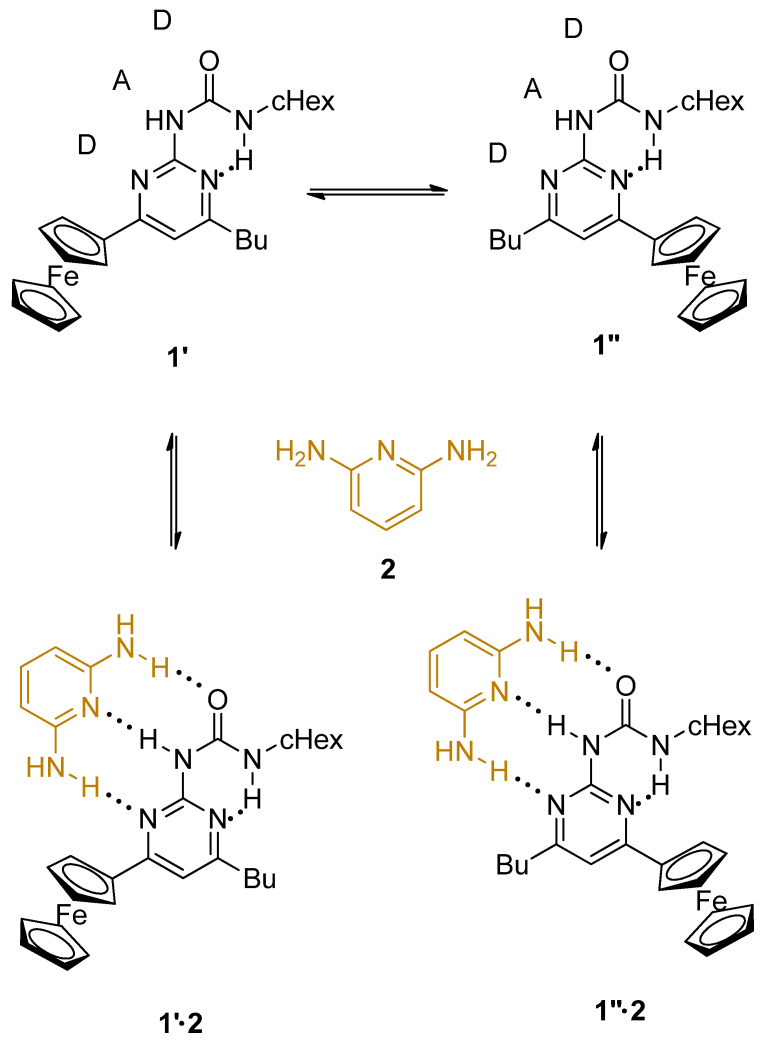
Conformers of ureidopyrimidine host **1** and its complexation with2,6-diaminopyridine (DAP, **2**).

During our previous studies, a series of 2-ureido-4-ferrocenylpyrimidine hosts (e.g., **1**, Figure 1) with an acceptor−donor−acceptor (ADA) bonding pattern were synthesized, and their complexation with 2,6-diaminopyridine (DAP, **2**) was followed by NMR spectroscopy or by electrochemical measurements in non-competitive solvents [24]. Also, the presence of an intramolecular hydrogen bond and the existence of two isomeric forms (**1**′ and **1**″) in solution were proved by NMR measurements and were supported by quantum chemical calculations. Based on the latter, conformer **1**′, with the ferrocenyl group pointing away from the urea moiety, could be assigned as the major isomer. (Calculations showed a similar preference for this conformer in the case of some other derivatives with alkyl and aryl substituents.) Both NMR results and calculations proved an increase in the ratio of the major conformer upon addition of DAP (**2**). It was also shown that by a proper modification of the urea side chain, the ureidopyrimidine could be immobilized on the surface of a carbon electrode via sol–gel electrodeposition [25].

Based on our previous studies, it was postulated that the introduction of a further heteroatom into the substituent of the pyrimidine ring might result in a new weak interaction between the host and guest (DAP). This might trigger a change in the conformational equilibria that could be traced by NMR spectroscopy or might lead to a stronger response to the presence of DAP in electrochemical measurements.

Accordingly, new derivatives with heterocycles attached to position C6 were prepared, and their solution-phase host–guest interactions were investigated by NMR spectroscopy, cyclic voltammetry, and quantum chemical calculations. Also, the modification of the surface of a spectral graphite electrode by the hosts and the effect of DAP on the electrochemical response of the modified electrode are presented.

## 2. Results and Discussion

### 2.1. Synthesis

**Scheme 1:**
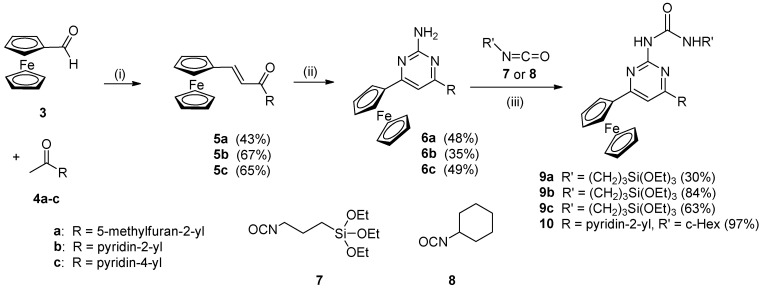
Synthetic route to novel 6-substituted 2-ureido-4-ferrocenylpyrimidines (**9a**–**c**, **10**) (reaction conditions: (i) NaOH, r.t., 10 min–1 h; (ii) guanidine carbonate, EtOH, reflux, 3 h; and (iii) 100 °C, 3 h).

New ureidopyrimidine derivatives (**9a**–**c**, **10**) containing a ferrocene moiety and a heterocyclic side chain have been synthesized using a three-step synthetic route based on procedures developed in our group previously (Scheme 1) [24,25]. Ferrocenyl-carboxaldehyde (**3**) was reacted with a heterocycle bearing an acetyl group (**4a**–**c**) in the presence of a base to give the corresponding enone (**5a**–**c**). The reaction was carried out under solvent-free conditions to avoid byproduct formation [26]. Isolated yields of the enone derivatives were only moderate (43–67%), due to the solidification of the reaction mixture upon conversion. The use of the ketone reagent in excess caused the formation of a byproduct in significant ratios via Michael addition of the ketone on the enone product (determined by GC-MS). Application of equimolar amounts of the organic reagents suppressed this undesired reaction.

Ferrocenyl-propenones (**5a**–**c**) were reacted in the next step with guanidinium carbonate and a base to provide 2-amino-4-ferrocenylpyrimidines (**6a**–**c**). The application of polar protic solvents (e.g., ethanol) turned out to be favorable in the heterocyclic ring-formation reaction. The excess guanidinium-carbonate could easily be removed from the mixture by extraction with water. Furthermore, the one-pot version of the first two synthetic steps showed that the purification of the enone derivatives prior to heterocycle formation is not necessary. The latter methodology led to products **6a**–**c** with approximately the same yields as the two-step procedure but produced less waste.

**Figure 2:**
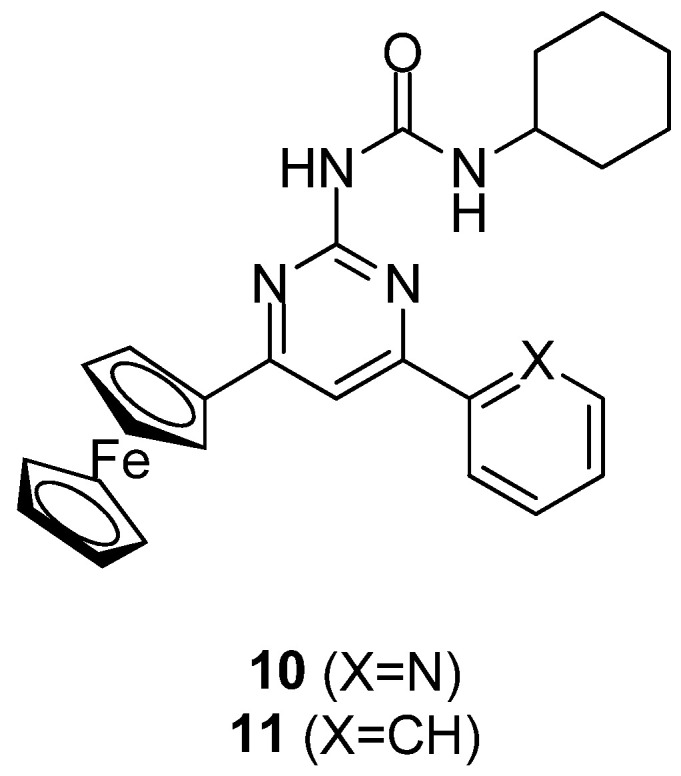
Structure of cyclohexylureas **10** and **11**.

6-Substituted-2-ureido-4-ferrocenylpyrimidines (**9a**–**c**, **10**) were obtained in the last step in the reaction of the aminopyrimidines (**6a**–**c**) with isocyanates (**7**, **8**). The reaction was conducted in a solvent-free way so that the catalytic effect of the product urea can prevail [27]. The excess isocyanate could be recycled after purification by column chromatography. Isocyanates were chosen to yield ureas suitable for immobilization (**7**) and for solution phase studies (**8**). The cyclohexyl derivative was chosen to make it possible to compare the properties of **10** and the 6-phenyl derivative (**11**, Figure 2) produced according to our previous study [24].

### 2.2. NMR Studies: Structure of Ureidopyrimidine Hosts

**Figure 3:**
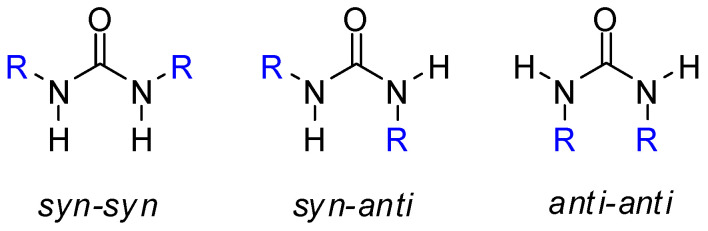
Possible conformations of *N*,*N*′-dialkylureas.

Ureidopyrimidines can be present in different conformations in solution depending on the relative position of the groups attached to the urea nitrogen atoms (Figure 3). Although quantum chemical calculations showed a preference for the syn–syn orientation in the case of *N*,*N*′-dialkylureas [28], an intramolecular hydrogen bond between the urea NH and the pyrimidine = N moieties in ureidopyrimidine-type molecules stabilizes the anti-syn conformation. Such structural orientation for similar compounds in the solid state was proved by X-ray crystallography [24]. Based on our previous results [24,29], a rapid interconversion between two conformers of ureidopyrimidines (**9a**–**c**, **10**, **11**) can be assumed at room temperature due to the rotation around the C2-N (urea) bond. In the present research, detailed NMR studies were carried out with compound **10** to reveal the effect of the pyridin-2-yl group on the conformational behavior of ureidopyrimidine hosts.

**Figure 4:**
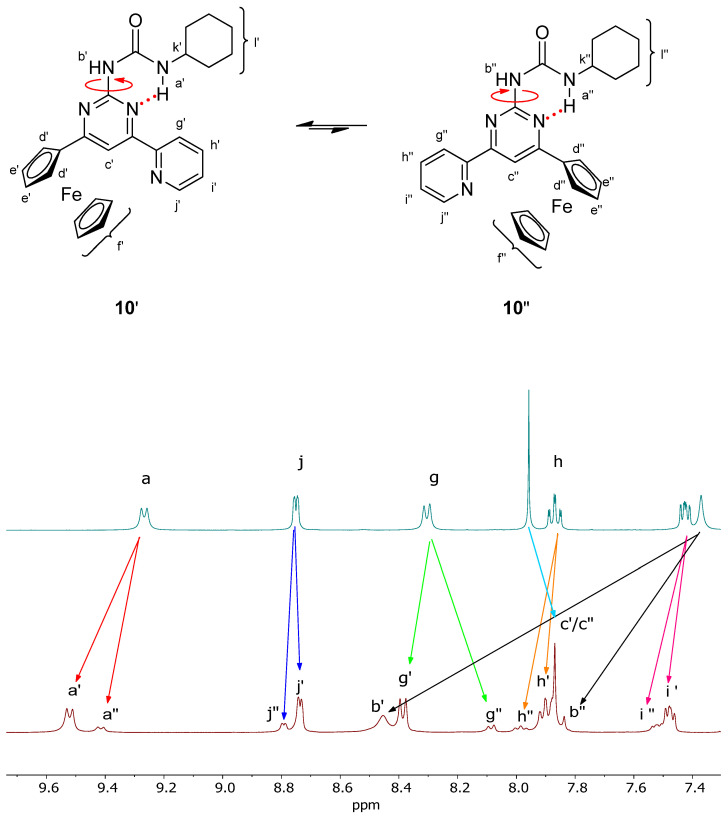
Part of the ^1^H NMR spectra of **10** at 300 K (**top**) and 209 K (**bottom**) (CDCl_3_, 78 mM **10**, 400 MHz).

**Figure 5:**
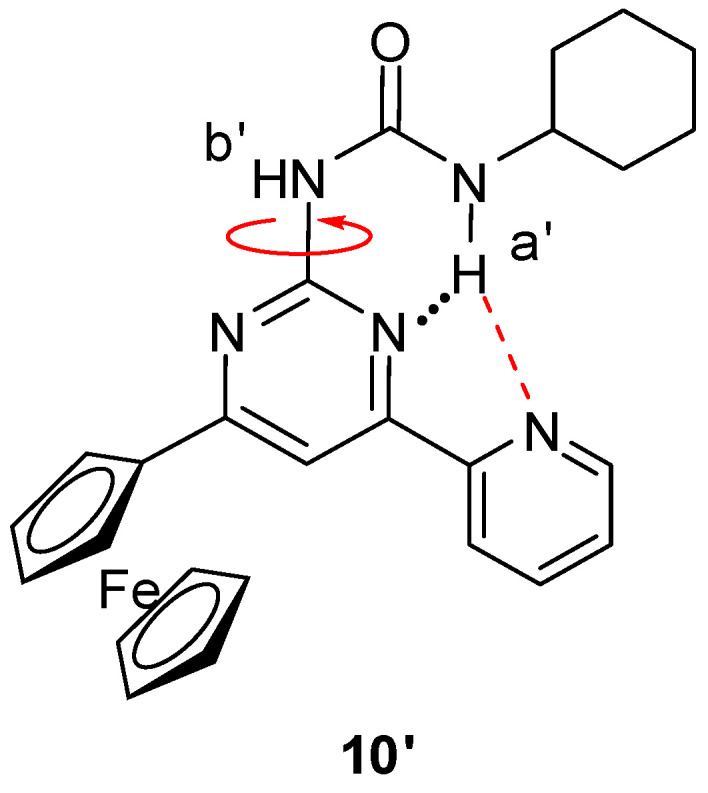
Assumed stabilization effect of the pyridin-2-yl group on the major conformer **10**′.

Low-temperature NMR measurements verified the coexistence of the two conformers. In contrast to the ^1^H NMR spectrum obtained at room temperature, two sets of signals could be detected for ferrocenyl, aromatic, and urea protons of **10** at 209 K (Figure 4 and Figure S1). (According to computational results discussed later, structure **10**′ is more stable, and thus, it is assigned as the major conformer.) A similar phenomenon could be observed for **9a**–**c** and **11** (Figures S2–S5); however, the ratio of the two conformers depended on the structure of the substituents (Table 1). Although some variation in these values can be observed in all cases, the pyridin-2-yl substituent on the pyrimidine ring led to a strong preference for the major conformer. (It should be mentioned that our previous results are in accordance with the data shown in entries 1, 3, and 6, Table 1, with minor/major conformer ratios of 0.85 and 0.67 for derivatives with R=R′=Ph and R=Bu, R′=cHex substituents, respectively [24].) The effect of the pyridine-2-yl group might be explained by a further weak interaction between pyridine nitrogen and NH_a_ (Figure 5). It can also be seen that the concentration has only a minimal effect on the ratio of conformers (entries 4 and 5).

**Figure 6:**
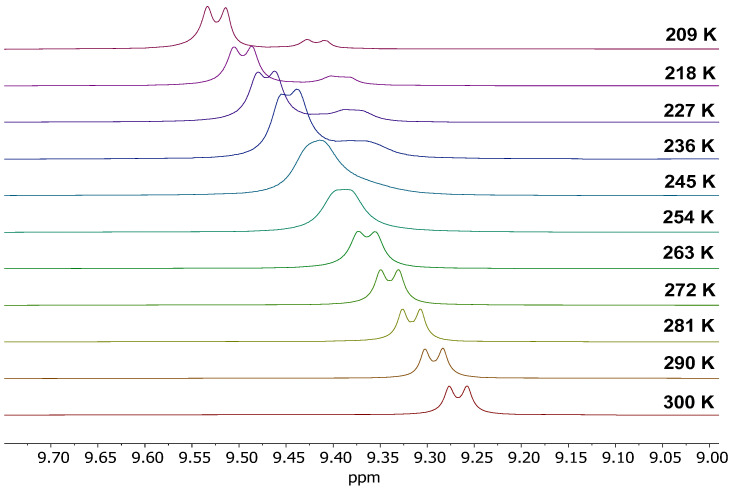
Coalescence of the signal of proton NH_a_ of **10** (CDCl_3_, 78 mM **10**, 400 MHz).

To investigate the temperature dependence and energies regarding the intramolecular movements, NMR spectra of **10** were acquired at different temperatures. The coalescence temperature of the two sets of NMR signals was found to be 245 K (Figure 6). Urea proton NH_b_ exhibited the largest splitting and shift upon cooling (Figure 4), presumably due to partial dimerization. (It should be mentioned that a considerably smaller shift could be observed at a lower concentration (Figure S1c)). The signals of the cyclohexyl protons varied the least (Figure S1, proton ‘l’), probably because of the stable equatorial position of the ureido group, which is justified by computational studies discussed later.

**Figure 7:**
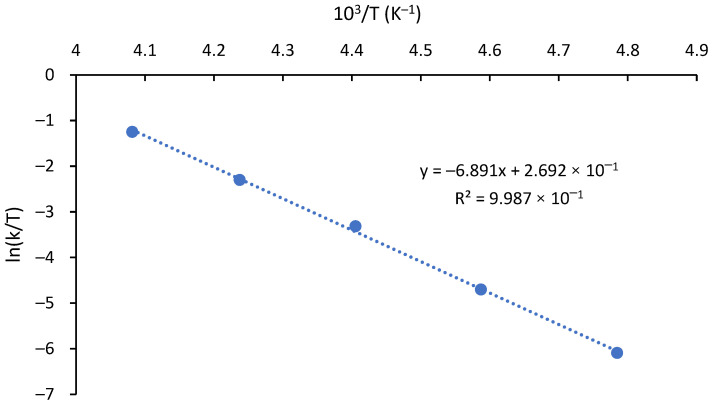
Eyring plot for the rotational conformation change reaction of **10**.

Lineshape fitting of NMR spectra around the coalescence temperature to the signal of proton NH_a_ of **10** was applied in order to gain the Eyring plot (Figure 7) for the rotational conformation change reaction of **10** (Table S1). Proton NH_a_ was found suitable for the lineshape fitting because in the NMR spectra of compound **10,** the peak is well isolated and does not move significantly to changes in temperature or concentration. The calculated enthalpy, entropy, and 298 K free energy of activation were 57.3 kJ/mol, 26.3 J/molK, and 49.5 kJ/mol, respectively.

The lineshape fitting of NMR spectra of **11** at 209 K yielded reaction rate constant (k) values greater than that of **10** (Table S2 and Figure S6). This outcome presumes that the conformational change happens less readily in the case of **10**, possibly because the additional weak interaction with the pyridin-2-yl group hinders the rotation around the C–N bond and stabilizes the **10**′ form (Figure 5). The k values of ureidopyrimidines **9a**–**c** are also in agreement with this theory (Table 1).

**Figure 8:**
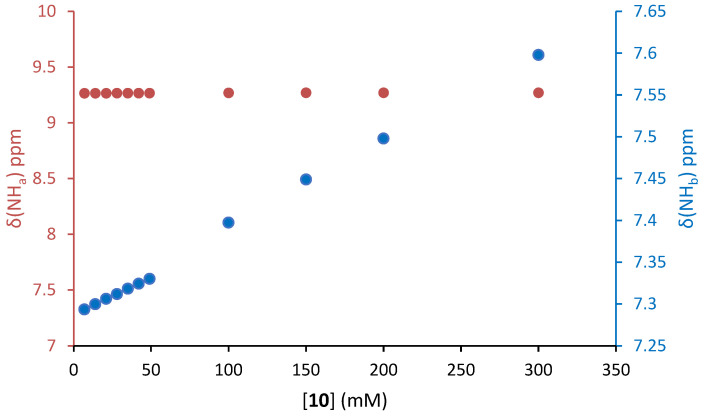
Change of chemical shift of urea protons with the concentration of **10** (CDCl_3_, 299 K, 400 MHz).

**Figure 9:**
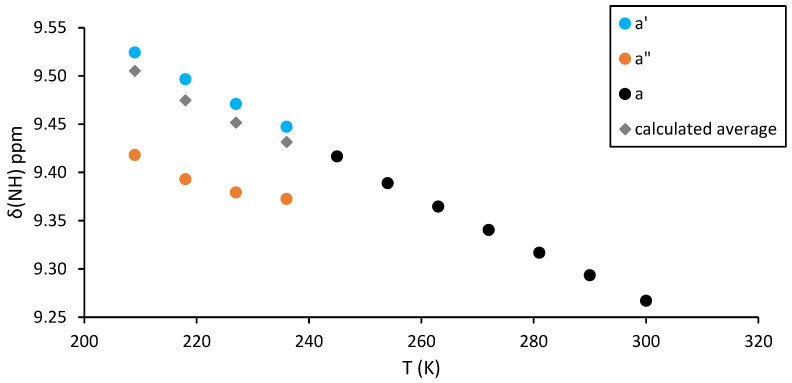
Temperature dependence of the chemical shift of NH_a_ proton of **10** (CDCl_3_, 78 mM **10**, 400 MHz) Calculated average values at each temperature are given as the mole-fraction-weighted sum of the chemical shifts of a′ and a″ protons of the conformers.

**Figure 10:**
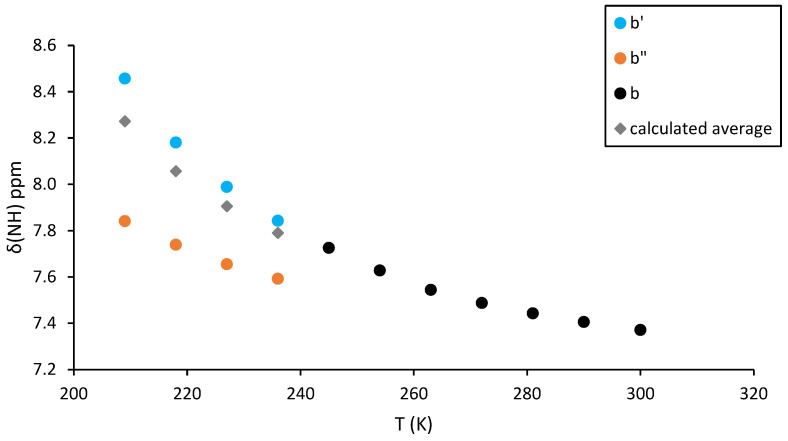
Temperature dependence of the chemical shift of NH_b_ proton of **10** (CDCl_3_, 78 mM **10**, 400 MHz) Calculated average values at each temperature are given as the mole-fraction-weighted sum of the chemical shifts of b′ and b″ protons of the conformers.

In the temperature range of 300 K–245 K, the ^1^H NMR spectra contained a signal with a strong downfield shift (9.2–9.4 ppm at room temperature) and a singlet (7.2–7.4 ppm at room temperature) corresponding to the urea NH protons (a and b for **10**, respectively) (Figure S7). This assignation is also confirmed by the ^1^H–^15^N–HSQC NMR spectrum of compound **10** (Figure S8). The former signal displayed concentration-independent behavior (Figure 8 and Figure S9) and moved even more downfield at low temperatures (Figure 6, Figure 9, and Figure S7), while the latter showed concentration dependence (Figure 8 and Figure S9) and temperature dependence (Figure 10 and Figure S7) as well. (Similar features were observed for ureidopyrimidine **11** (Figures S10–S15).)

**Scheme 2:**
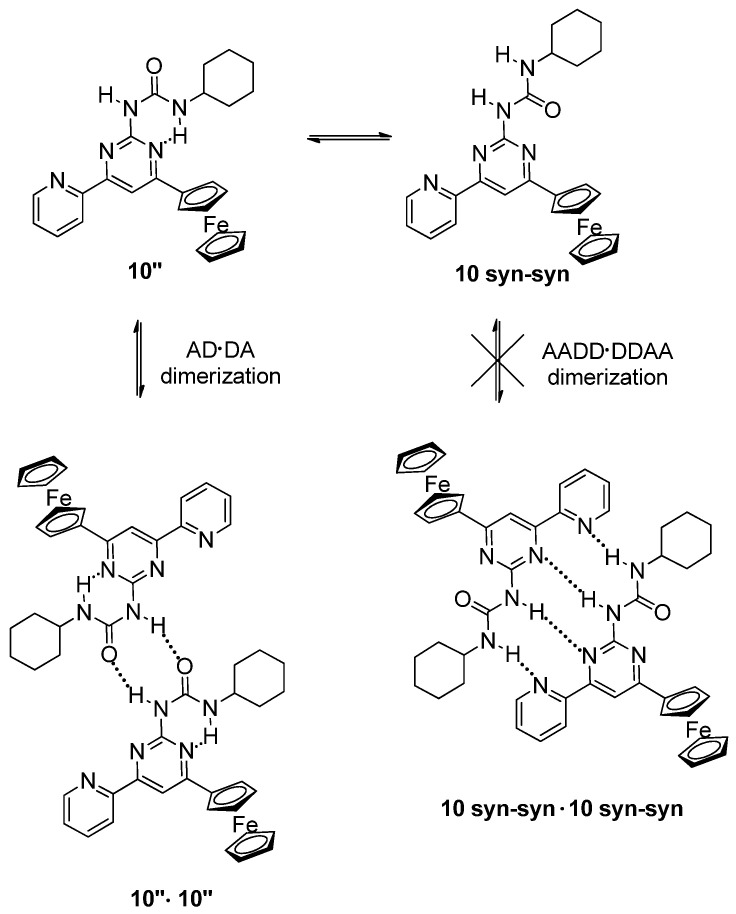
Some possible dimerization pathways of **10**.

Intramolecularly hydrogen-bonded protons are insensitive to changes in concentration [30]; however, elevating the temperature causes the break of the hydrogen bond, and thus the nucleus becomes more shielded [31]. Chemical shift change values (Δδ/ΔT) (measured in non-bonding solvent) higher than −5 ppb/K imply that the proton in question is mostly engaged in a hydrogen bond or not H-bonded at all [32]. The downfield resonance, NH_a_, displays a −2.6 ppb/K change and is found in the same region as intramolecularly H-bonded protons (11.3–7.7 ppm range based on benzoylureas) [33]. All this suggests that this resonance is that of an intramolecularly H-bonded proton. However, the upfield signal, belonging to urea proton NH_b_, displayed both dilution and temperature dependence. The latter phenomenon can be attributed either to solvent viscosity changes [34] or dimerization. Δδ/ΔT values lower than −6 ppb/K indicate that the H-bonded (most likely dimerized) and the solvent-exposed states of the NH proton are in equilibrium [35]. Negative temperature dependence of the chemical shift of −10.7 ppb/K for this resonance for compound **10** is in accordance with this theory. NH resonances of protons engaged in intermolecular H-bonds in urea dimers appear approximately in the 9.9 ppm–9.3 ppm chemical shift range in the NMR spectra according to detailed investigation involving the self-assembly of urea-calix [4]arenes [36,37]. This value is higher than the chemical shift of the NH_b_ proton even at the highest concentration measured (300 mM), which is close to the solubility limit. The δ(NH_b_) vs. concentration curve (Figure 8) is far from saturation in this concentration range, which implies a small extent of association. Also, the Δδ/ΔT value is much lower at the concentration of 10 mM (−3.3 ppb/K) (Figure S1c) compared to the rate mentioned above (−10.7 ppb/K for the 78 mM solution), which supports a low association in diluted solutions. The internally hydrogen-bonded conformers of **10** can form AD∙DA-type dimers (e.g., **10**″∙**10**″, Scheme 2). This assumption is justified by the structure of a similar ureidopyrimidine derivative [24], which was found to form such a dimer in the solid state as proved by X-ray crystallography. At the same time, dissociation of the intramolecular H-bond may result in conformational changes that might lead to a dimer of the AADD∙DDAA type (**10syn–syn**∙**10syn–syn**, Scheme 2) in the case of ureidopyrimidines with a pyridin-2-yl substituent. However, the existence of such a quadruply hydrogen-bonded structure is unlikely due to the nonlinearity of the AADD motif. It can also be refuted experimentally by the quasi-independence of the chemical shift of the internally H-bonded urea proton NH_a_ regarding changes in concentration (Figure 8). It should be mentioned that no additional information could be retrieved from spectra acquired at 185 K in CD_2_Cl_2_, so further low-temperature experiments were carried out in CDCl_3_ at 209 K.

### 2.3. Complexation Studies: NMR Titration

**Figure 11:**
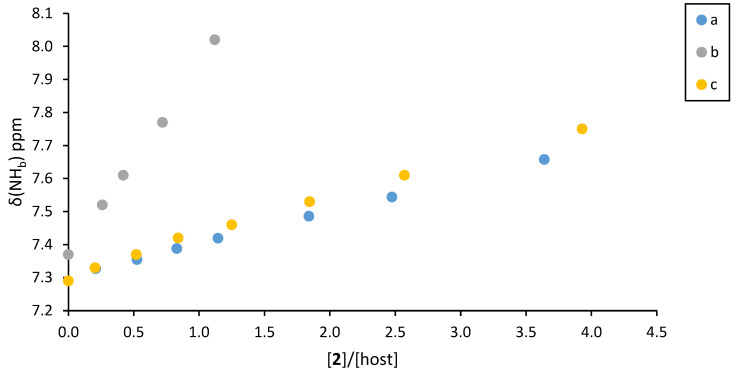
Changes in the chemical shift values of proton NH_b_ of hosts upon titration with **2** (a: 10 mM **10**, b: 78 mM **10**, c: 10 mM **11**, CDCl_3_, 300 K, 400 MHz).

In the next experiments, the formation of host–guest complexes of ureidopyrimidines was investigated. ^1^H NMR spectra acquired upon increasing the ratio of guest **2** bearing a DAD H-bonding pattern show the downfield shift of the NH_b_ proton of the host molecule **10** capable of intermolecular H-bonding (Figure 11 and Figure S16). (Hosts **9a**, **9b,** and **11** displayed similar behavior upon titration with **2** (Figures S17–S19).) The change in the chemical shift, that is, the response to the presence of DAP (**2**), is similar for hosts **10** and **11** but depends greatly on the concentration: at an elevated concentration of **10**, the change of δ(NH_b_) is greater (Figure 11, Figures S16 and S20).

**Figure 12:**
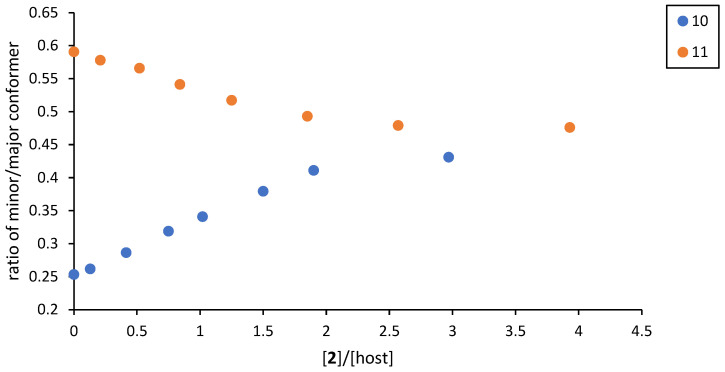
Change in the ratio of minor/major conformers upon titration with **2** (CDCl_3_,10 mM **10** and **11**, 209 K, 400 MHz).

Spectra taken at low temperatures show that the ratio of the two different conformers of the host also changes upon complex formation (Figure 12). In the NMR spectra, only an average signal of free hosts and their complexes with **2** can be seen; no separate resonances appear for the adducts, probably due to weak association. Since the ratio of integrals of NH_b_″/NH_b_′ signals in the spectra (Figures S21 and S26) contains the unbound hosts as well, the change in the ratio of host conformers **H**″/**H**′ can only originate from a shift in the ratio of the adducts formed with **2**, since the ratio of free host conformers is a constant value at a given temperature (see K_conf_ in Table 2).

**Figure 13:**
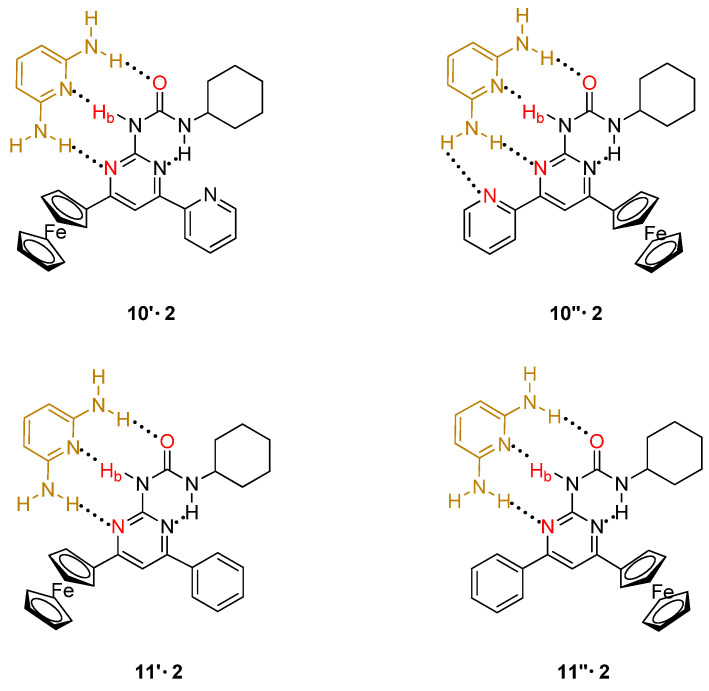
Structure of possible complexes of **2** and conformers of **10** (**10**′ and **10**″) and **11** (**11**′ and **11**″).

In contrast to the complexation of alkyl/aryl-substituted hosts investigated before [24], the ratio of the two different complexes is shifted towards the complex of the minor conformer (**10**″∙**2**, Figure 13), with an increase in the total minor/total major ratio from 0.25 to 0.43 upon addition of 3.0 equivalents of DAP (**2**) at 209 K (Figure 12, Figures S21 and S22). This could be attributed to the DAP-inflicted shift of equilibrium towards the minor conformer **10**″, suggesting a change in the relative stabilities of the complexes as compared to the free conformers of host **10**. (see computational studies). At a higher concentration of **10**, the downfield shift of NH_b_′ and NH_b_″ resonances is similar to that of the diluted sample (Figures S23 and S24), although the change in the **10**″/**10**′ ratio is moderately greater (Figure S25). In contrast, titration of host **11** with DAP (**2**) resulted in a decrease in the amount of the minor conformer (Figure 12), with a change in the ratio of **11**″/**11**′ from 0.59 to 0.48 upon addition of 3.9 equivalents of **2**, showing an increase in the stability of **11**′∙**2** at the expense of **11**″∙**2** (Figure 13, Figures S26 and S27). The latter observation is in accordance with NMR and computational results obtained for different alkyl/aryl substituted derivatives previously [24].

**Scheme 3:**
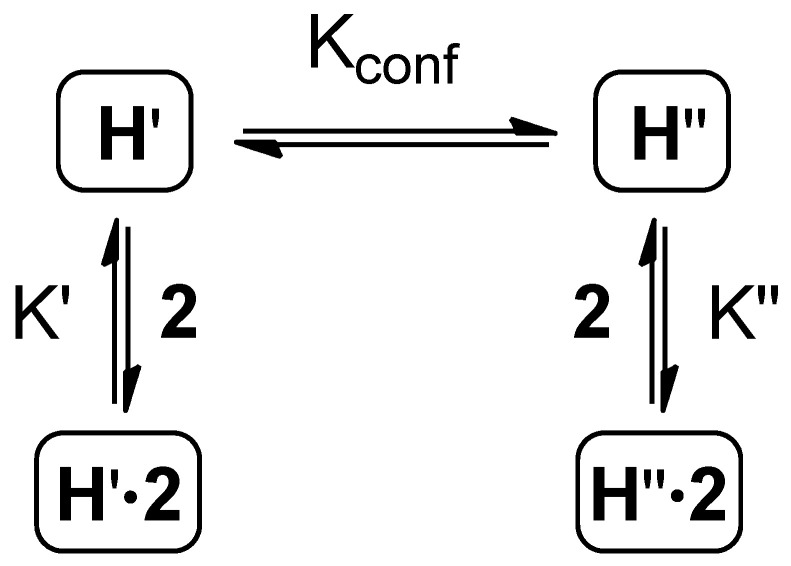
Assumed scheme of the coupling of guest-binding and conformational change (**H**′/**H**″: major/minor host, **H**′∙**2**/**H**″∙**2**: host–guest complex of major/minor conformer, K_conf_: equilibrium constant of the conformational change, K′/K″: association constant of the host–guest complex formed from major/minor conformer and **2**).

**Figure 14:**
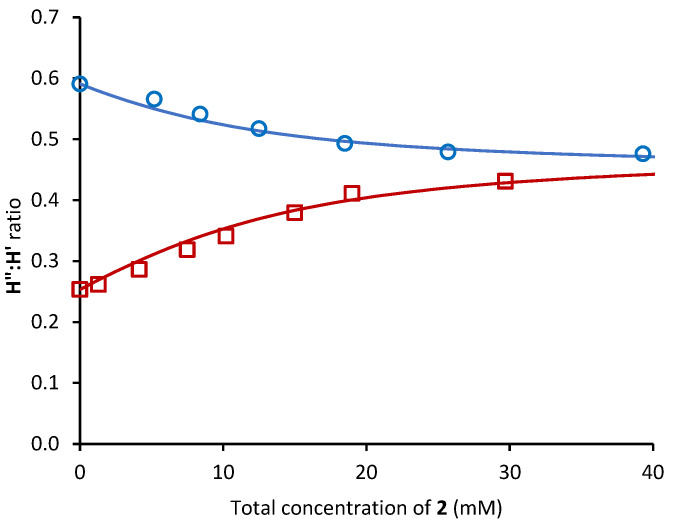
Measured (symbols) and calculated (solid lines) ratios of minor (**H**″) and major (**H**′) conformers. The red line and square symbols represent the data obtained for **10**, and the blue line and circle symbols represent the data obtained for **11**.

**Figure 15:**
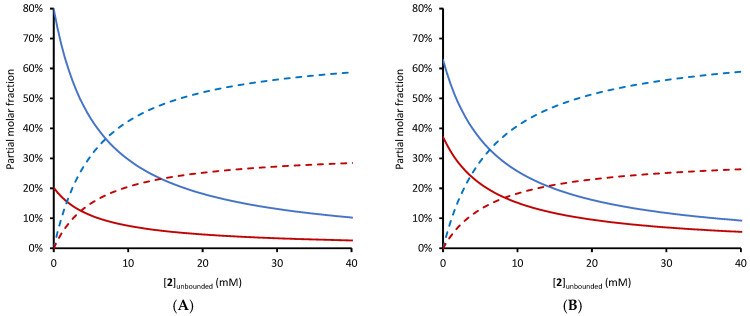
Partial molar fraction of compound **10** (**A**) and **11** (**B**). The red and blue lines represent the minor (″) and major (′) conformers, respectively. Dashed lines show the calculated data for adducts, while solid lines indicate the fractions of free host molecules.

The individual association constants of the major and minor conformers of **10** and **11** were determined based on the model depicted in Scheme 3 (as described in Section 3.4) by fitting a curve on the ratio of the total concentration of conformers **H**″/**H**′ (Figure 14) determined from low-temperature NMR spectra (Figure S22 for **10** and Figure S27 for **11**). This way the partial molar fraction functions of the species of systems **10**+**2** and **11**+**2** could also be calculated (Figure 15). The results are shown in Table 2. The association constants K′ in the case of **10**, **11,** and K″ of **11** are relatively low compared to the association constant of complex **10**″∙**2**, which is probably the result of the contribution of the pyridin-2-yl functionality to the binding of DAP (**2**). In addition, the ratio of K″/K′ shows a high contrast for the two hosts: 1.91 ± 0.1 for **10** and 0.758 ± 0.005 for **11**. These relations indicate that while minor conformer **10**″ binds **2** more effectively than **10**′, the case is reversed for compound **11**, and there is a greater difference in the binding affinities of the conformers of **10** than that of **11**. Considering the stability constant of association K_ass_, which accounts for the overall binding ability of the species, it can be seen that compound **10** has an overall higher affinity towards **2** than **11** does.

### 2.4. Electrochemical Measurements in Solution

**Figure 16:**
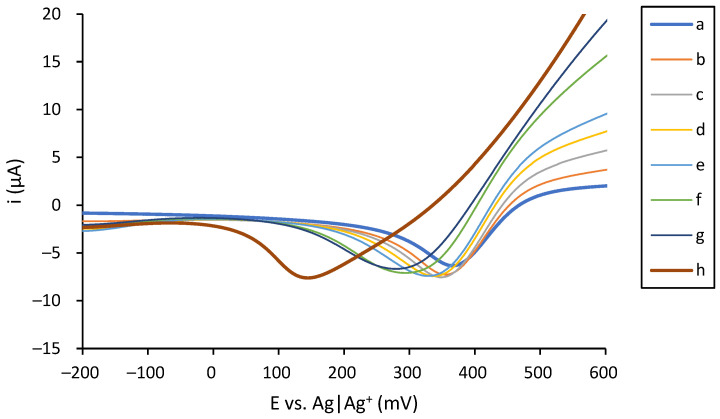
Linear sweep voltammetric (LSV) response of **2**/**10** mixtures (0.56 mM **10** in CH_2_Cl_2_, glassy carbon working electrode (OD = 3 mm), Ag|Ag^+^ reference electrode, Pt-wire counter electrode, 0.1 M [*n*Bu_4_N][PF_6_] supporting electrolyte, 100 mVs^−1^ scan rate, equivalents of **2**: a: 0, b: 0.5, c: 1.0, d: 1.5, e: 2.0, f: 4.0, g: 5.0, h: 20.0).

**Figure 17:**
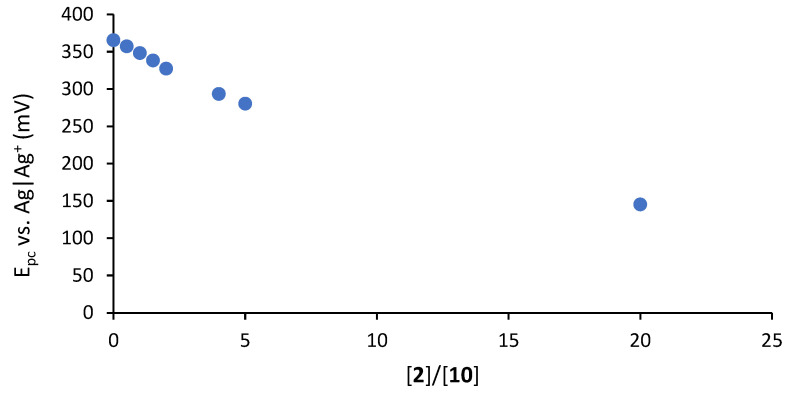
Change of the cathodic peak potential of LSV response curves of **10** as a function of the [2]/[10] ratio (0.56 mM **10** in CH_2_Cl_2_, glassy carbon working electrode (OD = 3 mm), Ag|Ag^+^ reference electrode, Pt-wire counter electrode, 0.1 M [*n*Bu_4_N][PF_6_] supporting electrolyte, 100 mVs^−1^ scan rate).

First, we explored the electrochemical behavior of **10** in organic media (Figures S28–S31). The cyclic voltammograms of **10** showed a quasi-reversible redox pair characteristic of the ferrocenyl moiety with an acceptable peak separation of 105 mV and a half-wave potential of 397 mV vs. the Ag|Ag^+^ reference electrode. Next, the electrochemical response of **10** on the addition of **2** was investigated in solution. In accordance with previous experiments [24], involving *n*Bu derivative **1**, a cathodic shift of the cathodic peak potential (E_pc_)values was observed by cyclic voltammetry (Figures S32 and S33). A similar change was detected during the titration of **11** with **2** (Figures S34 and S35). However, the flattening of the peaks and the disappearance of the reduction wave show that the electrochemical process becomes irreversible quickly at higher concentrations of **2**. This can be explained by the undesired polymerization reaction of **2** (Figure S36). It can be seen that the oxidation wave of the polymerization of **2** overlaps with the oxidation peak of **10** to such an extent that the Fc/Fc^+^ peak cannot be distinguished. Because of this, in further experiments only the change in E_pc_ was monitored; that seems to be undisturbed by the polymerization. Comparing the LSV curves of **10** (Figure 16, Figure 17, and Figure S37) with **11** (Figures S38 and S39) upon stepwise addition of **2**, it can be seen that the change in the reduction potential of **10** was greater than that of **11**, showing the beneficial effect of the pyridin-2-yl group in the electrochemical sensing of **2**.

### 2.5. Immobilization of Host Molecules

In the next step, the possibility of the electrochemical detection of DAP (**2**) with the help of electrodes modified with ureidopyrimidine species was investigated.

**Figure 18:**
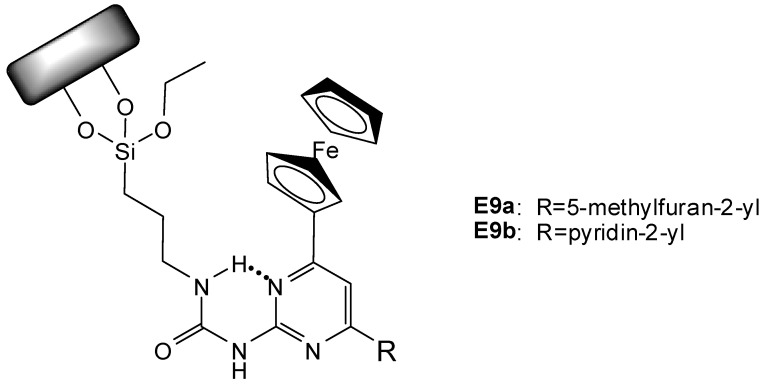
Structure of modified electrodes **E9a** and **E9b**.

**Figure 19:**
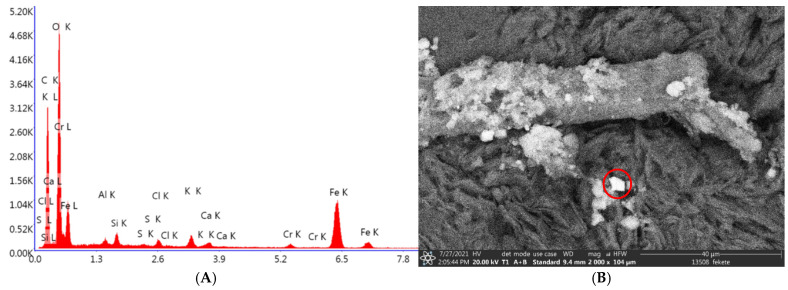
Elemental composition of the surface of **E9b** (**A**) (for the area indicated with a red circle on image (**B**)) and scanning electron microscopic (SEM) image of the surface of **E9b** (**B**) (FEI/Thermo Fisher Apreo S SEM equipment, high vacuum, Octane Elect Plus EDS detector, and 5.0 and 20.0 kV accelerating voltage).

According to a procedure reported previously [25], immobilization of ureidopyrimidines **9a** and **9b** on the surface of spectral graphite electrodes (**E9a** and **E9b**, Figure 18) was carried out by sol–gel electrodeposition in an ethanol–water mixture in the presence of a silica precursor, tetraethyl orthosilicate, to aid the incorporation of the host into a silica framework. SEM images of the graphite electrode modified with **9b** (**E9b**) proved the presence of **9b** deposited on the surface (Figure 19 and Table S3).

### 2.6. Electrochemical Measurements with Immobilized Hosts

**Figure 20:**
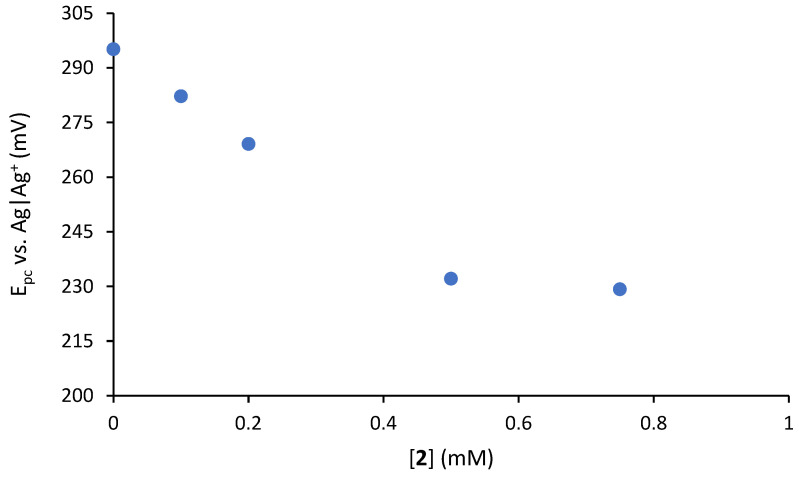
Change of the cathodic potential of **E9b** as a function of **2** concentration (Ag|Ag^+^ reference electrode, Pt-wire counter electrode, 0.1 M [*n*Bu_4_N][PF_6_] supporting electrolyte in CH_2_Cl_2_, 100 mVs^−1^ scan rate).

Cyclic voltametric measurements were carried out using the modified graphite electrodes (**E9a** and **E9b**) to examine the detection of **2** and to compare the effect of the introduction of two different heteroatoms into the pyrimidine substituent. As expected on the basis of electrochemical measurements with **2**/**10** mixtures (see Section 2.4), a cathodic shift of the E_pc_ was noticed in the case of the immobilized host **E9b** (Figure 20 and Figure S40). The E_pc_ of **E9b** seemed to reach saturation around 0.5 mM concentration of **2**. In contrast, the cathodic peak potential of the furanyl derivative **E9a** did not change significantly (Figures S41 and S42). These results again show that the incorporation of the pyridin-2-yl moiety results in a greater response to the presence of guest **2**.

### 2.7. Computational Studies

Quantum chemical computations were employed to investigate the relative stabilities of host (**10**′ vs. **10**″) molecules as well as those of the adducts (**10**′∙**2**, **10**″∙**2**), calculating molecular geometries and energies using the composite method r^2^SCAN-3c in combination with the SMD solvation model employing CHCl_3_ as solvent. In line with the ratio of conformers in the low-temperature NMR spectra, species **10**′ was found to be more stable by 3.6 kJ/mol in comparison to conformer **10**″. Assuming a Boltzmann distribution (p_1_/p_2_ = exp(−ΔG/RT)), the ratio of the two stable conformers is 87%:13%, which is a good agreement with the experimental 80%:20% ratio. For the free energy barrier of the conformational change, 61.5 kJ/mol was obtained at 298 K, which is slightly higher than the experimentally obtained barrier (49.5 kJ/mol).

**Figure 21:**
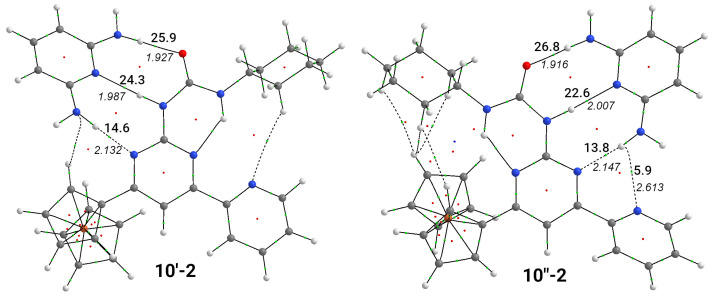
Bond paths of adducts **10**′∙**2** and **10**″∙**2** with estimated H-bond energy values (kJ/mol) and bond distances (in italics, Å). Weak interactions with less than 4 kJ/mol interaction energies are omitted for clarity.

Forming adducts with **2** decreases the free energy difference between the two conformers to 1.1 kJ/mol, with **10**′∙**2** still being more stable with a ratio of 65%:35%. The increased relative stability of adduct **10**″∙**2** may be attributed to an excess hydrogen bond between the pyridine nitrogen and a hydrogen of the amino group. The hydrogen bond strength can be estimated according to Espinosa and coworkers, taking into account the half value of the potential energy density at the bond critical point [38] that can be evaluated within the framework of the QTAIM methodology. For both adducts, the strongest H-bonds are attributed to the interaction between the urea oxygen and the NH_2_ group of 2,6-diaminopyridine (Figure 21). The interaction strength of the second NH_2_ group with the nitrogen atom of the pyrimidine moiety is approximately half of the O–NH_2_ interaction. For **10**″∙**2**, one of the NH_2_ hydrogens also interacts with the pyridine nitrogen with an estimated interaction strength of 5.9 kJ/mol, enabling a stronger binding for the diaminopyridine guest molecule.

## 3. Materials and Methods

### 3.1. General Comments

The following reagents were purchased and used without any further purification: 2-acetylpyridine, 2-acetyl-5-methylfuran, 4-acetylpyridine, cyclohexyl isocyanate, tetraethyl orthosilicate, 2,6-diaminopyridine (Aldrich, Burlington, MA, USA), 3-isocyanatopropyl triethoxysilane (AK Scientific, Union City, CA, USA), guanidine carbonate (Acros Organics, Antwerpen, Belgium), sodium hydroxide, cetyl trimethylammonium bromide (Molar Chemicals, Halásztelek, Hungary), potassium nitrate, potassium chloride (Reanal, Budapest, Hungary), and tetrabutylammonium hexafluorophosphate (TCI, Tokyo, Japan). Solvents were dried by routine procedures and stored on previously heated molecular sieves under an Ar atmosphere. Compound **11** was synthesized, as described earlier by Fehér et al. [24]. Reactions were monitored using Merck (Rahway, NJ, USA) Silicagel F_254_ TLC plates. Melting points of synthesized compounds were determined on a Kofler hot-plate instrument (HMK Franz Küstner Nacht BG, Dresden, Germany). ^1^H and {^1^H}^13^C NMR measurements were carried out in CDCl_3_ on a Bruker Avance 400 (Billerica, MA, USA) spectrometer (running TopSpin 2.1. software) at 400 and 100 MHz. Chemical shift values (δ) are given relative to CHCl_3_ (7.26 and 77.00 ppm for ^1^H and ^13^C, respectively) in ppm. GC-MS measurements were carried out on a Shimadzu GCMS-QP2010 SE instrument (Kyoto, Japan) (running GCMS solution 4.11 SU2 software). Infrared spectra were recorded on an IRAffinity 1-S Shimadzu instrument (Kyoto, Japan) using ATR crystal topping(running LabSolutions IR 2.26 software). HRMS measurements were carried out on aQ-TOF Premier mass spectrometer (Waters Corporation, Milford, MA, USA) using MassLynx 4.1 software. Scanning electron microscopic measurements were carried out on a FEI/Thermo Fisher Apero S SEM instrument (Waltham, MA, USA) in high vacuum in scanning mode using the Octane Elect Plus EDS detector, applying 5.0 and 20.0 kV accelerating voltage (running EDAX TEAM 4.5 software). Electrochemical measurements were performed on a BioLogic SAS SP-150 potentiostat (Claix, France) (running EC-LAB v.11.41 software).

### 3.2. Electrochemistry

Electrochemical measurements were carried out on a BioLogic SP-150 potentiostat (using EC-LAB v.11.41 software). All measurements were performed in a one-compartment, three-electrode cell configuration containing a reference electrode Ag|Ag^+^|[*n*Bu_4_N][ClO_4_] (0.01 M AgNO_3_, 0.1 M [*n*Bu_4_N][ClO_4_] in MeCN), a glassy carbon working electrode (OD = 3 mm), and a counter electrode (Pt-wire). Cyclic voltammetric measurements were performed at room temperature under Ar in CH_2_Cl_2_ with 0.1 M [*n*Bu_4_N][PF_6_] as the supporting electrolyte (with a cell volume of 10 cm^3^) at a scan rate of 100 or 500 mVs^−1^, forward scanning. Starting potential was set to the open channel potential. All electrodes were at a distance of 1 cm from each other. All samples were previously degassed with Ar bubbling, and the glassy carbon electrode was polished on a 0.5 μm alumina slurry after each measurement.

### 3.3. Lineshape Fitting

The *dnmr* module of Bruker’s TopSpin 3.6.5 program was used to carry out lineshape fitting of low-temperature NMR spectra. During the fitting of spectra, the fitting window (F1P and F2P) was selected to contain only proton H_j_ of compound **10** and NH_b_ in the case of compound **11**. One spin system was allocated to contain protons H′ and H″, and for the fitting of coupling constants, the program was allowed to create the signal of a mutually coupled proton (Nuc3) outside the fitting window so that the best overlap can be achieved. Besides the chemical shift values (Nu(iso)) and coupling constants (J1 and J2) of signals of H′ and H″, the reaction rate constant (k) of the conformation change reaction and the mole fraction (X) of conformers of **10** and **11** were also fitted. The fittings were accepted if the overlap percentage rose above 95%.

### 3.4. Determination of Equilibrium Constants

K_conf_ was determined from the integrals of peaks of NH_b_′ and NH_b_″ of NMR spectra obtained at 209 K. K′ and K″ individual association constants were determined by a fitting procedure using the Solver tools of MS Excel.

The overall association constant (K_ass_) can be expressed as follows:

(1) $$H+2⇌H\cdot 2\;\;\;\;\;\;\;K_{ass}=\frac{\left[H\cdot 2\right]}{\left[H\right][2]}$$

(2) $$K_{ass}=\frac{K^{\prime }}{1+K_{conf}}+\frac{K^{\prime \prime }}{1+1/K_{conf}}.$$

Equilibrium constants were calculated based on the following equations for host **H** (**10** or **11**):

(3) $$H^{\prime }⇌H^{\prime \prime }\;\;\;\;\;\;\;K_{conf}=\frac{\left[H^{\prime \prime }\right]}{\left[H^{\prime }\right]}$$

(4) $$H^{\prime }+2⇌H^{\prime }\cdot 2\;\;\;\;\;\;\;K^{\prime }=\frac{\left[H^{\prime }\cdot 2\right]}{\left[H^{\prime }\right][2]}$$

(5) $$H^{\prime \prime }+2⇌H^{\prime \prime }\cdot 2\;\;\;\;\;\;\;K^{\prime \prime }=\frac{\left[H^{\prime \prime }\cdot 2\right]}{\left[H^{\prime \prime }\right][2]}$$

(6) $$\left[H^{\prime \prime }\right]=K_{conf}[H^{\prime }]$$

(7) $$\left[H^{\prime }\cdot 2\right]=K^{\prime }\left[H^{\prime }\right][2]$$

(8) $$\left[H^{\prime \prime }\cdot 2\right]=K^{\prime \prime }\left[H^{\prime \prime }\right]\left[2\right]=K^{\prime \prime }K_{conf}[H^{\prime }]\left[2\right]$$

Total analytical concentrations of **H** (T_H_) and **2** (T_**2**_) can be calculated by Equations (9) and (10):

(9) $$T_{H}=[H^{\prime }]\left(1+K_{Conf}+K^{\prime }\left[2\right]+K^{\prime \prime }K_{Conf}[2]\right),$$

(10) $$T_{2}=\left[H^{\prime }\right]\left[2\right]\left(K^{\prime }+K^{\prime \prime }K_{Conf}\right)+[2].$$

The x′ and x″ molar fractions were calculated from the chemical shifts of NH_b_ protons of **H** (Figure S22 for **10** and Figure S27 for **11**) by fitting a saturation curve to the experimental data to obtain δNH_b_ (complexed) as a saturation value:

(11) $$x^{\prime }=\frac{{∆\delta NH^{\prime }}_{b}}{{∆\delta NH^{\prime }}_{b}\left(complexed\right)-{∆\delta NH^{\prime }}_{b}(free)},$$

(12) $$x^{\prime \prime }=\frac{{∆\delta NH^{\prime \prime }}_{b}}{{∆\delta NH^{\prime \prime }}_{b}\left(complexed\right)-{∆\delta NH^{\prime \prime }}_{b}(free)}.$$

and these fractions can also be expressed as follows:

(13) $$x^{\prime }=\frac{\left[H^{\prime }\cdot 2\right]}{\left[H^{\prime }\cdot 2\right]+[H^{\prime }]},$$

and

(14) $$x^{\prime }=\frac{\left[H^{\prime \prime }\cdot 2\right]}{\left[H^{\prime \prime }\cdot 2\right]+[H^{\prime \prime }]}.$$

The minor/major ratio (*R*) obtained from the ratio of the area of the corresponding NH_b_ peaks can be expressed as follows:

(15) $$R=\frac{\left[H^{\prime \prime }\cdot 2\right]+[H^{\prime \prime }]}{\left[H^{\prime }\cdot 2\right]+[H^{\prime }]}.$$

From Equations (13)–(15), Equation (16) can be obtained as follows:

(16) $$\frac{x^{\prime }}{x^{\prime \prime }}=\frac{\left[H^{\prime }\cdot 2\right]}{\left[H^{\prime \prime }\cdot 2\right]}\cdot \frac{\left[H^{\prime \prime }\cdot 2\right]+\left[H^{\prime \prime }\right]}{\left[H^{\prime }\cdot 2\right]+\left[H^{\prime }\right]}=\frac{\left[H^{\prime }\cdot 2\right]}{\left[H^{\prime \prime }\cdot 2\right]}\cdot R.$$

x′/x″ and *R* can be obtained from experimental data (see above), while

(17) $$\frac{\left[H^{\prime }\cdot 2\right]}{\left[H^{\prime \prime }\cdot 2\right]}=\frac{K^{\prime }}{K_{conf}K^{\prime \prime }}.$$

K_conf_ was determined from the integrals of peaks of NH_b_’ and NH_b_″ of NMR spectra obtained at 209 K. This way, K′/K″ can be calculated from experimental data. Using this ratio K′, [**H**] and [**2**] can be fitted by using Equations (9) and (10).

### 3.5. Computational Details

All the structures were optimized without symmetry constraints with tight convergence criteria with the Orca 6.0 program package [39,40] using the r^2^SCAN-3c composite method that is based on the r^2^SCAN meta-GGA functional [41] combined with the D4 dispersion correction [42] and a geometrical counter-poise correction in combination with the modified def2-mTZVPP basis set. The r^2^SCAN-3c method is particularly suitable for the accurate evaluation of geometries, thermochemistry, and non-covalent interactions [43]. The stationary points were characterized by frequency calculations in order to verify that they have zero imaginary frequencies for equilibrium geometries and one imaginary frequency for the transition state. Thermochemistry corrections were taken from frequency calculations at 298.15 K and 1 atm. QTAIM (Quantum Theory of Atoms in Molecules) analyses of the wavefunction [44] were carried out with the AIMAll Version 19.10.12. software [45]. On the optimized geometries, the energies were recomputed at the same level of theory, including solvation corrections utilizing the SMD [46] model, with dielectric constant ε = 4.81 for CDCl_3_.

### 3.6. Synthesis and Characterization of Synthesized Compounds

#### 3.6.1. Ferrocenyl-Propenones **5a**–**c**

Ferrocenyl-carboxaldehyde (**3**) (1 mmol, 214.0 mg) and sodium hydroxide (1 mmol, 40.0 mg) were measured into a Schlenk tube, and the atmosphere was changed to argon. Then, **4a**/**4b**/**4c** (1 mmol, 124.1 mg, 116 μL **4a**; 121.1 mg, 112 μL **4b**; 121.1 mg, 111 μL **4c**) was added under a stream of argon, and the reaction mixture was stirred by a magnetic stirrer for 1 h at 25 °C. The progress of the reaction was monitored by TLC. Column chromatography of the mixture (silica gel) yielded **5a**/**5b**/**5c** as purple solids.

*(E)-1-(5-Methylfuran-2-yl)-3-ferrocenylprop-2-en-1-one* (**5a**) Yield: 43% (137.6 mg); R_f_ = 0.42 (toluene/EtOAc 8:1); m.p. 142–146 °C; ^1^H NMR (400 MHz, CDCl_3_, 25 °C, TMS, ppm): δ = 7.77 (d, ^3^J_H,H_ = 15.5 Hz, 1H; Fc–CH=CH–C=O), 7.20–7.18 (m, 1H; Fur–H3), 6.97 (d, ^3^J_H,H_ = 15.5 Hz, 1H; Fc–CH=CH–C=O), 6.20–6.18 (m, 1H; Fur–H4), 4.59 (t, ^3^J_H,H_ = 1.9 Hz, 2H; Cp), 4.47 (t, ^3^J_H,H_ = 1.9 Hz, 2H; Cp), 4.17 (s, 5H; Cp), 2.44–2.42 (m, 3H; CH_3_); ^13^C{^1^H} NMR (101 MHz, CDCl_3_, 25 °C, TMS, ppm): δ = 177.0, 157.7, 152.8, 145.2, 118.9, 118.5, 109.3, 79.4, 71.4, 69.9, 69.1, 14.4; IR (ATR, cm^−1^): ṽ = 1654 (s) (C=O), 1600 (s) (C=C); ESI–MS *m*/*z* (%): 343 (100) [M + Na]^+^, 320 (20) [M]^+^; HRMS (ESI) *m*/*z* calcd for C_18_H_16_O_2_Fe+Na^+^: 343.0397 [M + Na]^+^; found: 343.0384.
*(E)-1-(Pyridin-2-yl)-3-ferrocenylprop-2-en-1-one* (**5b**) Yield: 67% (212.5 mg); R_f_ = 0.54 (hexane/EtOAc 4:1); m.p. 156–160 °C; ^1^H NMR (400 MHz, CDCl_3_, 25 °C, TMS, ppm): δ = 8.76–8.71 (m, 1H; Py–H6), 8.21–8.16 (m, 1H; Py–H3), 7.91 (d, ^3^J_H,H_ = 15.8 Hz, 1H; Fc–CH=CH–C=O, overlapped), 7.88–7.83 (m, 1H; Py–H4, overlapped), 7.84 (d, ^3^J_H,H_ = 15.8 Hz, 1H; Fc–CH=CH–C=O, overlapped), 7.43–7.50 (m, 1H; Py–H5), 4.68 (t, ^3^J_H,H_=1.9 Hz, 2H; Cp), 4.50 (t, ^3^J_H,H_ = 1.9 Hz, 2H; Cp), 4.18 (s, 5H; Cp); ^13^C{^1^H} NMR (101 MHz, CDCl_3_, 25 °C, TMS, ppm): δ = 188.6, 154.9, 148.9, 147.3, 137.1, 126.7, 123.0, 118.0, 79.6, 71.6, 70.0, 69.6; IR (ATR, cm^−1^): ṽ = 1661 (m) (C=O), 1591 (m) (C=C); ESI–MS *m*/*z* (%): 318 (100) [M + H]^+^, 340 (98) [M + Na]^+^; HRMS (ESI) *m*/*z* calcd for C_18_H_15_NOFe+Na^+^: 340.0401 [M + Na]^+^; found: 340.0405.
*(E)-1-(Pyridin-4-yl)-3-ferrocenylprop-2-en-1-one* (**5c**) Yield: 65% (206.2 mg); R_f_ = 0.16 (CHCl_3_/EtOAc 8:1); m.p. 145–150 °C; ^1^H NMR (400 MHz, CDCl_3_, 25 °C, TMS, ppm): δ = 8.89–8.74 (m, 2H; Py–H2,6), 7.79 (d, ^3^J_H,H_ = 15.4 Hz, 1H; Fc–CH=CH–C=O), 7.76–7.69 (m, 2H; Py–H3,5), 7.01 (d, ^3^J_H,H_ = 15.4 Hz, 1H; Fc–CH=CH–C=O), 4.61 (t, ^3^J_H,H_ = 1.9 Hz, 2H; Cp), 4.55 (t, ^3^J_H,H_ = 1.9 Hz, 2H; Cp), 4.19 (s, 5H; Cp); ^13^C{^1^H} NMR (101 MHz, CDCl_3_, 25 °C, TMS, ppm): δ = 189.0, 150.8, 149.5, 145.1, 121.6, 118.2, 78.7, 72.2, 70.1, 69.5; IR (ATR, cm^−1^): ṽ = 1655 (s) (C=O), 1578 (s) (C=C); ESI–MS *m*/*z* (%): 318 (100) [M + H]^+^; HRMS (ESI) *m*/*z* calcd for C_18_H_16_NOFe^+^: 318.0581 [M + H]^+^; found: 318.0583.

#### 3.6.2. 2-Amino-6-ferrocenylpyrimidines **6a**–**c**

**5a**/**5b**/**5c** (2.5 mmol, 800.5 mg **5a**/793.0 mg **5b**/793.0 mg **5c**), guanidine carbonate (5 mmol, 900.9 mg), and sodium hydroxide (5 mmol, 200.0 mg) were measured into a Schlenk tube, and the atmosphere was changed to argon. Ethanol (5 mL) was added under a stream of argon. The Schlenk tube was equipped with a condenser and a buffer tank filled with argon, and then the reaction mixture was stirred with a magnetic stirrer and refluxed in an oil bath for 3 h at 95 °C. The progress of the reaction was monitored by TLC. After the completion of the reaction, the solvent was removed in vacuo. The crude product was dissolved in dichloromethane, and the excess guanidine carbonate was removed with an aqueous extraction. Column chromatography of the mixture (silica gel) yielded **6a**/**6b**/**6c** as an orange-brown solid.

*2-Amino-4-ferrocenyl-6-(5-methylfuran-2-yl)-pyrimidine* (**6a**) Yield: 48% (431.0 mg); R_f_ = 0.43 (toluene/EtOAc 1:1); m.p. decomposed at 200 °C; ^1^H NMR (400 MHz, CDCl_3_, 25 °C, TMS, ppm): δ = 7.07–7.04 (m, 1H; Fur–H3), 7.01 (s, 1H; Pyr–H), 6.18–6.15 (m, 1H; Fur–H4), 5.09 (bs, 2H; NH_2_), 4.97 (t, ^3^J_H,H_ = 1.9 Hz, 2H; Cp), 4.45 (t, ^3^J_H,H_ = 1.9 Hz, 2H; Cp), 4.11 (s, 5H; Cp), 2.45 (bs, 3H; CH_3_); ^13^C{^1^H} NMR (101 MHz, CDCl_3_, 25 °C, TMS, ppm): δ = 169.3, 163.2, 155.8, 155.0, 150.9, 112.6, 108.8, 101.5, 81.2, 70.8, 70.1, 68.1, 14.2; IR (ATR, cm^−1^): ṽ = 3306 (b) (NH_2_), 3169 (b) (NH_2_), 1568 (s) (Pyr); ESI–MS *m*/*z* (%): 360 (100) [M + H]^+^; HRMS (ESI) *m*/*z* calcd for C_19_H_18_N_3_OFe^+^: 360.0799 [M + H]^+^; found: 360.0795.
*2-Amino-4-ferrocenyl-6-(pyridin-2-yl)-pyrimidine* (**6b**) Yield: 35% (311.7 mg); R_f_ = 0.24 (EtOAc); m.p. 166–168 °C; ^1^H NMR (400 MHz, CDCl_3_, 25 °C, TMS, ppm): δ = 8.76–8.71 (m, 1H; Py–H6), 8.39–8.34 (m, 1H; Py–H3), 7.86–7.81 (m, 1H; Py–H4), 7.75 (s, 1H; Pyr–H), 7.41–7.35 (m, 1H; Py–H5), 5.11 (bs, 2H; NH_2_), 5.05 (t, ^3^J_H,H_ = 1.9 Hz, 2H; Cp), 4.47 (t, ^3^J_H,H_ = 1.9 Hz, 2H; Cp), 4.11 (s, 5H; Cp); ^13^C{^1^H} NMR (101 MHz, CDCl_3_, 25 °C, TMS, ppm): δ = 170.4, 163.3, 163.2, 155.1, 149.5, 137.1, 124.9, 121.7, 104.4, 81.3, 70.9, 70.1, 68.3; IR (ATR, cm^−1^): ṽ = 3323 (b) (NH_2_), 3190 (b) (NH_2_), 1560 (s) (Pyr); ESI–MS *m*/*z* (%): 357 (100) [M + H]^+^; HRMS (ESI) *m*/*z* calcd for C_19_H_17_N_4_Fe^+^: 357.0803 [M + H]^+^; found: 357.0799.
*2-Amino-4-ferrocenyl-6-(pyridin-4-yl)-pyrimidine* (**6c**) Yield: 49% (436.4 mg); R_f_ = 0.44 (EtOAc); m.p. decomposed at 210 °C; ^1^H NMR (400 MHz, CDCl_3_, 25 °C, TMS, ppm): δ = 8.70–8.84 (m, 2H; Py–H3,5), 7.93–7.85 (m, 2H; Py–H2,5), 7.13 (s, 1H; Pyr–H), 5.23 (bs, 2H; NH_2_), 4.98 (t, ^3^J_H,H_ = 1.9 Hz, 2H; Cp), 4.49 (t, ^3^J_H,H_ = 1.9 Hz, 2H; Cp), 4.10 (s, 5H; Cp); ^13^C{^1^H} NMR (101 MHz, CDCl_3_, 25 °C, TMS, ppm): δ=170.7, 163.6, 162.2, 150.6, 145.4, 121.2, 104.2, 80.8, 71.2, 70.2, 68.2; IR (ATR, cm^−1^): ṽ = 3321 (b) (NH_2_), 3094 (b) (NH_2_), 1557 (s) (Pyr); ESI–MS *m*/*z* (%): 357 (100) [M + H]^+^; HRMS (ESI) *m*/*z* calcd for C_19_H_17_N_4_Fe^+^: 357.0803 [M + H]^+^; found: 357.0805.

#### 3.6.3. 2-Ureido-4-ferrocenylpyrimidines **9a**–**c**, **10**

*1-(4-Ferrocenyl-6-(5-methylfuran-2-yl)-pyrimidin-2-yl)-3-triethoxysilylpropylurea* (**9a**) Yield: 30% (182.0 mg); R_f_ = 0.33 (CHCl_3_/EtOAc 4:1); m.p. decomposed at 217 °C; ^1^H NMR (400 MHz, CDCl_3_, 25 °C, TMS, ppm): δ = 9.41–9.33 (m, 1H; Si–CH_2_CH_2_CH_2_–NH), 7.22 (bs, 1H; CH_2_NH–CO–NH); 7.14 (s, 1H; Pyr–H) 7.08–7.04 (m, 1H; Fur–H3), 6.21–6.16 (m, 1H; Fur–H4), 4.94 (t, ^3^J_H,H_ = 1.8 Hz, 2H; Cp), 4.51 (t, ^3^J_H,H_ = 1.8 Hz, 2H; Cp), 4.11 (s, 5H; Cp), 3.83 (quart, ^3^J_H,H_ = 7.0 Hz, 6H; (CH_3_CH_2_O)_3_Si), 3.48–3.40 (m, 2H; Si–CH_2_CH_2_CH_2_–NH), 2.45 (bs, 3H; CH_3_), 1.87–1.77 (m, 2H; Si–CH_2_CH_2_CH_2_–NH), 1.23 (t, ^3^J_H,H_ = 7.0 Hz, 9H; (CH_3_CH_2_O)_3_Si), 0.80–0.74 (m, 2H; Si–CH_2_CH_2_CH_2_–NH); ^13^C{^1^H} NMR (101 MHz, CDCl_3_, 25 °C, TMS, ppm): δ = 158.0, 155.8, 154.7, 150.2, 113.7, 109.1, 103.3, 80.2, 71.4, 70.3, 68.2, 58.6, 42.9, 23.9, 18.5, 14.2, 8.2; IR (ATR, cm^−1^): ṽ = 3233 (b) (NHCONH), 1684 (m) (NHCONH),1576 (s) (Pyr), 1508 (m) (CONH), 1078 (m) (Si–O–C), 789 (b) (Si–CH_2_); ESI–MS *m*/*z* (%): 607 (50) [M + H]^+^, 629 (100) [M + Na]^+^; HRMS (ESI) *m*/*z* calcd for C_29_H_38_N_4_O_5_SiFeNa^+^: 629.1859 [M + Na]^+^; found: 629.1863.
*1-(4-Ferrocenyl-6-(pyridin-2-yl)-pyrimidin-2-yl)-3-triethoxysilylpropylurea* (**9b**) Yield: 84% (253.5 mg); R_f_ = 0.65 (EtOAc); m.p. 67 °C; ^1^H NMR (400 MHz, CDCl_3_, 25 °C, TMS, ppm): δ = 9.43–9.32 (m, 1H; (Si–CH_2_CH_2_CH_2_–NH), 8.81–8.66 (m, 1H; Py–H6) 8.35–8.20 (m, 1H; Py–H3), 7.95 (s, 1H; Pyr–H), 7.90–7.82 (m, 1H; Py–H4), 7.47 (bs, 1H; CH_2_NH–CO–NH), 7.44–7.38 (m, 1H; Py–H5), 5.00 (t, ^3^J_H,H_ = 1.9 Hz, 2H; Cp), 4.53 (t, ^3^J_H,H_ = 1.9 Hz, 2H; Cp), 4.10 (s, 5H; Cp), 3.83 (quart, ^3^J_H,H_ = 7.0 Hz, 6H; (CH_3_CH_2_O)_3_Si), 3.50–3.44 (m, 2H; Si–CH_2_CH_2_CH_2_–NH), 1.88–1.78 (m, 2H; Si–CH_2_CH_2_CH_2_–NH), 1.22 (t, ^3^J_H,H_ = 7.0 Hz, 9H; (CH_3_CH_2_O)_3_Si), 0.81–0.74 (m, 2H; Si–CH_2_CH_2_CH_2_–NH); ^13^C{^1^H} NMR (101 MHz, CDCl_3_, 25 °C, TMS, ppm): δ = 158.07, 154.66, 153.97, 149.61, 137.28, 125.48, 121.76, 106.57, 80.07, 71.60, 70.30, 68.41, 58.57, 42.97, 23.91, 18.47, 8.24; IR (ATR, cm^−1^): ṽ = 3250 (b) (NHCONH), 1680 (m) (NHCONH), 1576 (s) (Pyr), 1530 (m) (CONH), 1080 (m) (Si–O–C), 789 (b) (Si–CH_2_); ESI–MS *m*/*z* (%): 604 (82) [M + H]^+^, 626 (100) [*M* + Na]^+^; HRMS (ESI) *m*/*z* calcd for C_29_H_37_N_5_O_4_SiFeNa^+^: 626.1862 [M + Na]^+^; found: 626.1863.
*1-(4-Ferrocenyl-6-(pyridin-4-yl)-pyrimidin-2-yl)-3-triethoxysilylpropylurea* (**9c**) Yield: 63% (190.1 mg); R_f_ = 0.15 (CHCl_3_/EtOAc 1:1); m.p. decomposed at 190 °C; ^1^H NMR (400 MHz, CDCl_3_, 25 °C, TMS, ppm): δ = 9.27–9.21 (m, 1H; (Si–CH_2_CH_2_CH_2_–NH), 8.85–8.79 (m, 2H; Py–H2,6), 7.88–7.84 (m, 2H, Py–H3,5), 7.36 (bs, 1H; CH_2_NH–CO–NH); 7.30 (s, 1H; Pyr–H), 4.96 (t, ^3^J_H,H_ = 1.9 Hz, 2H; Cp), 4.59 (t, ^3^J_H,H_ = 1.9 Hz, 2H; Cp), 4.13 (s, 5H; Cp), 3.84 (quart, ^3^J_H,H_ = 7.0 Hz, 6H; (CH_3_CH_2_O)_3_Si), 3.50–3.43 (m, 2H; Si–CH_2_CH_2_CH_2_–NH), 1.88–1.78 (m, 2H; Si–CH_2_CH_2_CH_2_–NH), 1.23 (t, ^3^J_H,H_ = 7.0 Hz, 9H; (CH_3_CH_2_O)_3_Si), 0.80–0.74 (m, 2H; Si–CH_2_CH_2_CH_2_–NH); ^13^C{^1^H} NMR (101 MHz, CDCl_3_, 25 °C, TMS, ppm): δ=13C NMR (101 MHz, CDCl3) δ 158.49, 154.38, 150.92, 144.26, 121.07, 106.46, 79.49, 71.95, 70.41, 68.35, 58.61, 42.97, 23.87, 18.49; IR (ATR, cm^−1^): ṽ = 3260 (b) (NHCONH), 1682 (m) (NHCONH), 1584 (s) (Pyr), 1522 (m) (CONH), 1077 (m) (Si–O–C), 752 (b) (Si–CH_2_); ESI–MS *m*/*z* (%): 604 (100) [M + H]^+^, 626 (55) [M + Na]^+^; HRMS (ESI) *m*/*z* calcd for C_29_H_37_N_5_O_4_SiFeNa^+^: 626.1862 [M + Na]^+^; found: 626.1873.
*1-Cyclohexyl-3-(4-ferrocenyl-6-(pyridin-2-yl)-pyrimidin-2-ylurea* (**10**) Yield: 97% (292.7 mg); R_f_ = 0.50 (EtOAc); m.p. 142–146 °C; ^1^H NMR (400 MHz, 300 mM in CDCl_3_, 25 °C, TMS, ppm): δ=9.40–9.13 (m, 1H; ((cHex)CH–NH)), 8.82–8.67 (m, 1H; Py–H6), 8.40–8.20 (m, 1H, Py–H3), 7.94 (s, 1H; Pyr–H), 7.91–7.80 (m, 1H; Py–H4), 7.46–7.40 (m, 1H; Py–H5, overlapped), 7.37 (bs, 1H; CH–NH–CO–NH, overlapped), 4.98 (t, ^3^J_H,H_ = 1.9 Hz, 2H; Cp), 4.53 (t, ^3^J_H,H_ = 1.9 Hz, 2H; Cp), 4.09 (s, 5H; Cp), 3.96–3.76 (m, 1H; NH–CO–NH–CH(cHex)), 2.24–2.13 (m, 2H; cHex), 1.90–1.79 (m, 2H; cHex), 1.73–1.65 (m, 1H; cHex), 1.54–1.23 (m, 5H; cHex); ^13^C{^1^H} NMR (101 MHz, CDCl_3_, 25 °C, TMS, ppm): δ = 158.1, 154.1, 153.9, 149.6, 137.2, 125.5, 121.8, 106.5, 80.2, 71.6, 70.3, 68.4, 49.2, 34.1, 25.9, 25.3; IR (ATR, cm^−1^): ṽ = 3254 (b) (NHCONH), 1678 (m) (NHCONH), 1572 (s) (Pyr), 1524 (m) (CONH); ESI–MS *m*/*z* (%): 482 (100) [M + H]^+^, 504 (38) [M + Na]^+^; HRMS (ESI) *m*/*z* calcd for C_26_H_27_N_5_OFeNa^+^: 504.1463 [M + Na]^+^; found: 504.1454.

### 3.7. Immobilization of Ureidopyrimidines ***9a*** and ***9b*** on Electrode Surface, Preparation of Modified Electrodes ***E9a*** and ***E9b***

For the immobilization of the triethoxysilylpropyl derivatives **9a**,**b** a sol–gel electrodeposition method was used. The surface of the spectral graphite electrode was previously scoured on sandpaper and the electrode sonicated for a minute in dichloromethane, then in ethanol, and lastly in water. The immobilization mixture was prepared as follows. Ureidopyrimidine **9a** or **9b** (0.1 mmol, 60.7 mg **9a,** or 60.4 mg **9b**), potassium nitrate (2 mmol, 202.2 mg), cetyltrimethylammonium bromide (0.5 mmol, 182.2 mg), 8 cm^3^ ethanol, 2 cm^3^ water, and tetraethyl orthosilicate (0.4 mmol, 83.3 mg, 89.3 μL) were measured into a glass vessel, and the mixture was stirred at 25 °C for 15 min with a magnetic stirrer. Then, the immobilization mixture was transferred into the electrochemical cell and deoxygenated with Ar bubbling for 2 min. The previously prepared spectral graphite electrode was immersed into the mixture as a working electrode, Ag|AgCl|KCl as a reference electrode, and Pt-wire as a counter electrode. Moreover, 1300 mV voltage was applied to the mixture for 15 min, and then the electrode was placed in a 110 °C oven for 10 min and then in a desiccator filled with Ar to let it cool down.

## 4. Conclusions

Low-temperature NMR investigations prove the existence of two different conformers in the solutions of 2-ureido-4-ferrocenylpyrimidines **9a**–**c**, **10,** and **11**. Comparison of NMR data shows that the incorporation of a pyridin-2-yl substituent on C6 of the pyrimidine ring (compounds **9b** and **10**) has a strong influence on the ratio of the conformers, increasing the amount of the **x**′-type conformer at the expense of that of **x**″, probably due to a weak interaction between pyridine nitrogen and a urea NH. Moreover, the same moiety leads to a reversal of the binding strength of the conformers when entrapped in host–guest complexes formed with a guest bearing a DAD H-bonding pattern (DAP) and a higher overall association constant as compared to the phenyl-substituted host **11**. This can be explained by an extra weak interaction between pyridine nitrogen and an amino group of DAP in complex **10**″∙**2**. The structure and relative stability of free and complexed conformers of **10** were calculated by computational methods, and the results supported the conclusions drawn from the NMR experiments. The presence of the pyridin-2-yl group was found to be also advantageous in the electrochemical sensing of the DAP guest, both in solution and after immobilization of the host on an electrode, compared to phenyl- and furan-2-yl substituted hosts, respectively.

## Figures and Tables

**Table 1:** Ratio of major and minor conformers of ureidopyrimidines at 209 K.

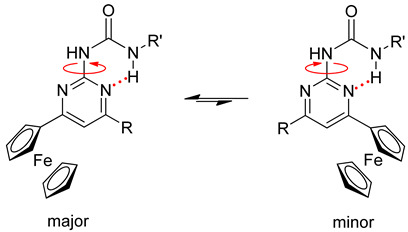						
Entry	Host	R	R′	Minor/Major ^a^	k (209 K) (Hz) ^a^	c (mM) ^b^
1	**9a**	5-methylfuran-2-yl	(CH_2_)_3_Si(OEt)_3_	0.70	1.01	5
2	**9b**	Pyridin-2-yl	(CH_2_)_3_Si(OEt)_3_	0.29	0.27	173
3	**9c**	Pyridin-4-yl	(CH_2_)_3_Si(OEt)_3_	0.59	2.96	50
4	**10**	Pyridin-2-yl	c-Hex	0.25	0.54	10
5	**10**	Pyridin-2-yl	c-Hex	0.26	0.47	78
6	**11**	Ph	c-Hex	0.59	2.38	10

^a^ Determined from lineshape fitting of spectra acquired at 209 K. ^b^ Concentration of the host in the NMR sample in CDCl_3_.

**Table 2:** Calculated association constants (K′, K″) and stability constant of association (K_ass_) for the complexation of compounds **10** and **11** with guest **2**.

	10	11
K_conf_	0.253	0.591
K″/K′	1.91 ± 0.1	0.758 ± 0.005
K′ (mM^−1^)	0.14 ± 0.03	0.16 ± 0.03
K″ (mM^−1^)	0.27 ± 0.05	0.12 ± 0.03
K_ass_ (mM^−1^)	0.17 ± 0.05	0.15 ± 0.03

## Data Availability

Data are contained within the article or Supplementary Materials.

## Supplementary Materials

The following supporting information can be downloaded at: https://www.mdpi.com/article/10.3390/ijms252413552/s1.
