# Supplementary material for: Investigation of Host–Guest Interactions in 2-Ureido-4-ferrocenylpyrimidine Derivatives
Source: Int J Mol Sci. 2024 Dec 18;25(24):13552. doi: 10.3390/ijms252413552 (PMC11677767; doi:10.3390/ijms252413552)
Supplement: Supplementary file 1 [file ijms-25-13552-s001.zip › ijms-3372643-supplementary.pdf]

Supporting information

Investigation of host-guest interactions in 2-ureido-4-ferrocenylpyrimidine derivatives

Márk Váradi [1], Soma J. Keszei [2], Ágnes Gömöry [3], Margit Kovács [4], Tamás Kégl [5,6,7], Lajos Fodor [8] and Rita Skoda-Földes [1,*]

[1] Research Group of Organic Synthesis and Catalysis, University of Pannonia, Egyetem u. 10, 8200 Veszprém, Hungary; varadi.mark@mk.uni-pannon.hu

[2] Centre for Energy Research, Institute of Technical Physics and Materials Science, Konkoly-Thege út 29-33, 1121 Budapest, Hungary; keszei.soma@ek.hun-ren.hu

[3] MS Proteomics Research Group, Hungarian Research Network, Research Centre for Natural Sciences, Magyar tudósok körútja 2, 1117 Budapest, Hungary; gomory.agnes@ttk.hu

[4] NMR Laboratory, University of Pannonia, Egyetem u. 10, 8200 Veszprém, Hungary; kovacs.margit@mk.uni-pannon.hu

[5] Department of General and Inorganic Chemistry, University of Pécs, Ifjúság útja 6, 7624 Pécs, Hungary; tkegl@gamma.ttk.pte.hu

[6] János Szentágothai Research Center, Ifjúság útja 34, 7624 Pécs, Hungary

[7] HUN-REN-PTE Research Group for Selective Chemical Syntheses, Ifjúság útja 6, 7624 Pécs, Hungary

[8] Research Group of Environmental and Inorganic Photochemistry, University of Pannonia, Egyetem u. 10, 8200 Veszprém, Hungary; fodor.lajos@mk.uni-pannon.hu

***** Correspondence: skodane.foldes.rita@mk.uni-pannon.hu

S1. Supplementary NMR data

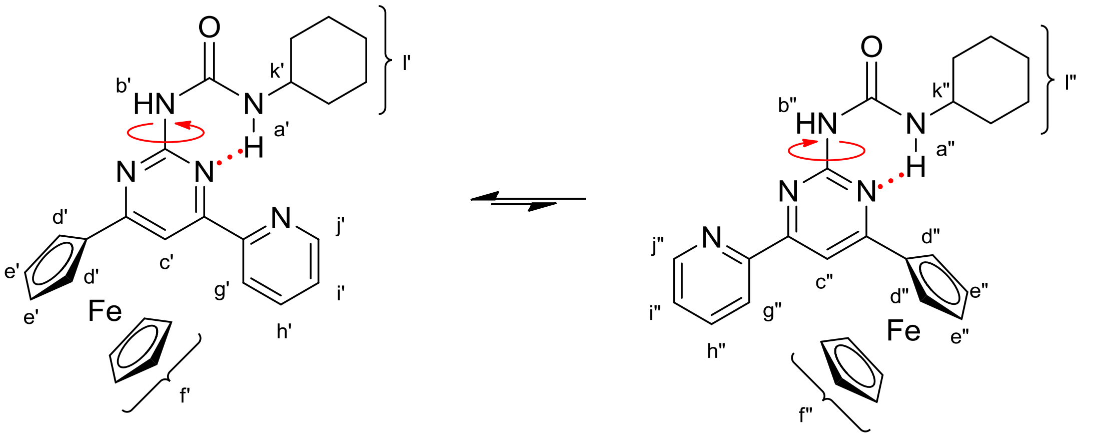

10' **10''**

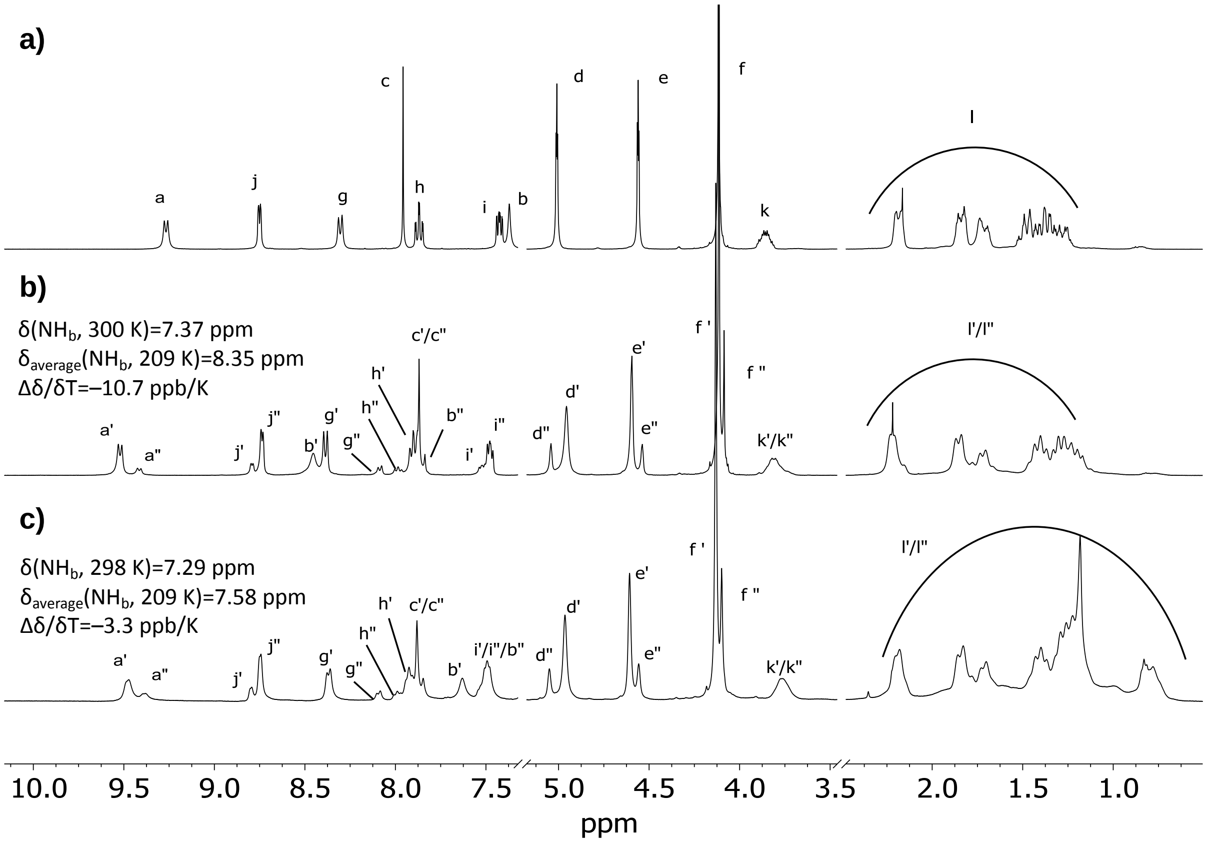

Figure S1. ^{1}H NMR spectra of **10 a**) at 300 K, 78 mM **10**; **b**) at 209 K, 78 mM **10**; **c**) 209 K, 10 mM **10** ($CDCl_3$, 400 MHz).

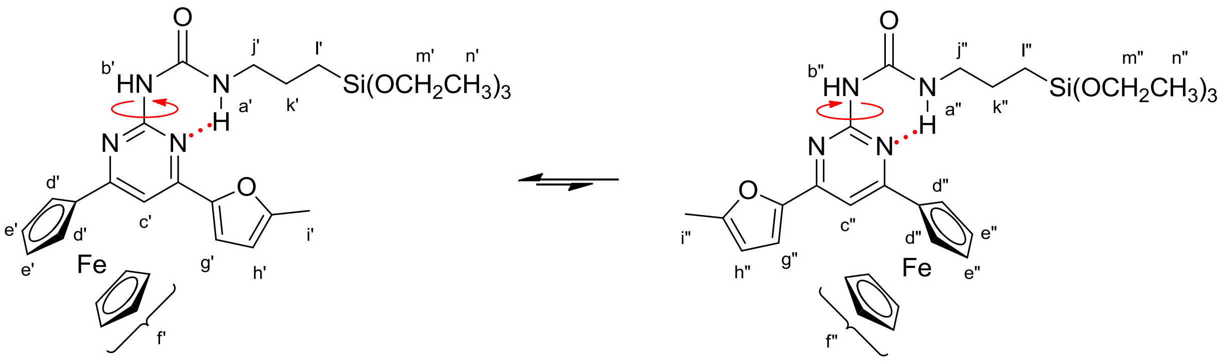

9a' **9a"**

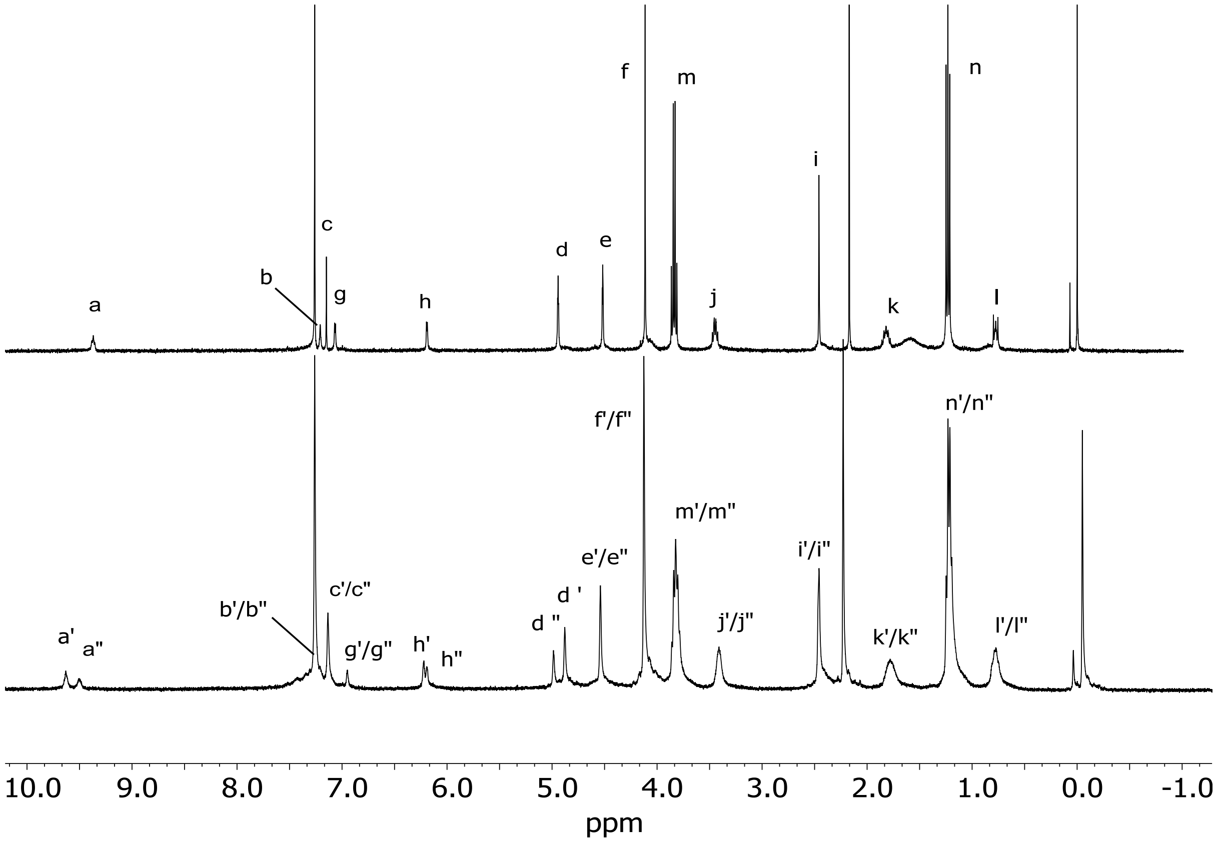

Figure S2. ^{1}H NMR spectra of **9a** at 300 K (top) and **9a**'/**9a**" at 209 K (bottom) ($CDCl_3$, 5 mM **9a**, 400 MHz).

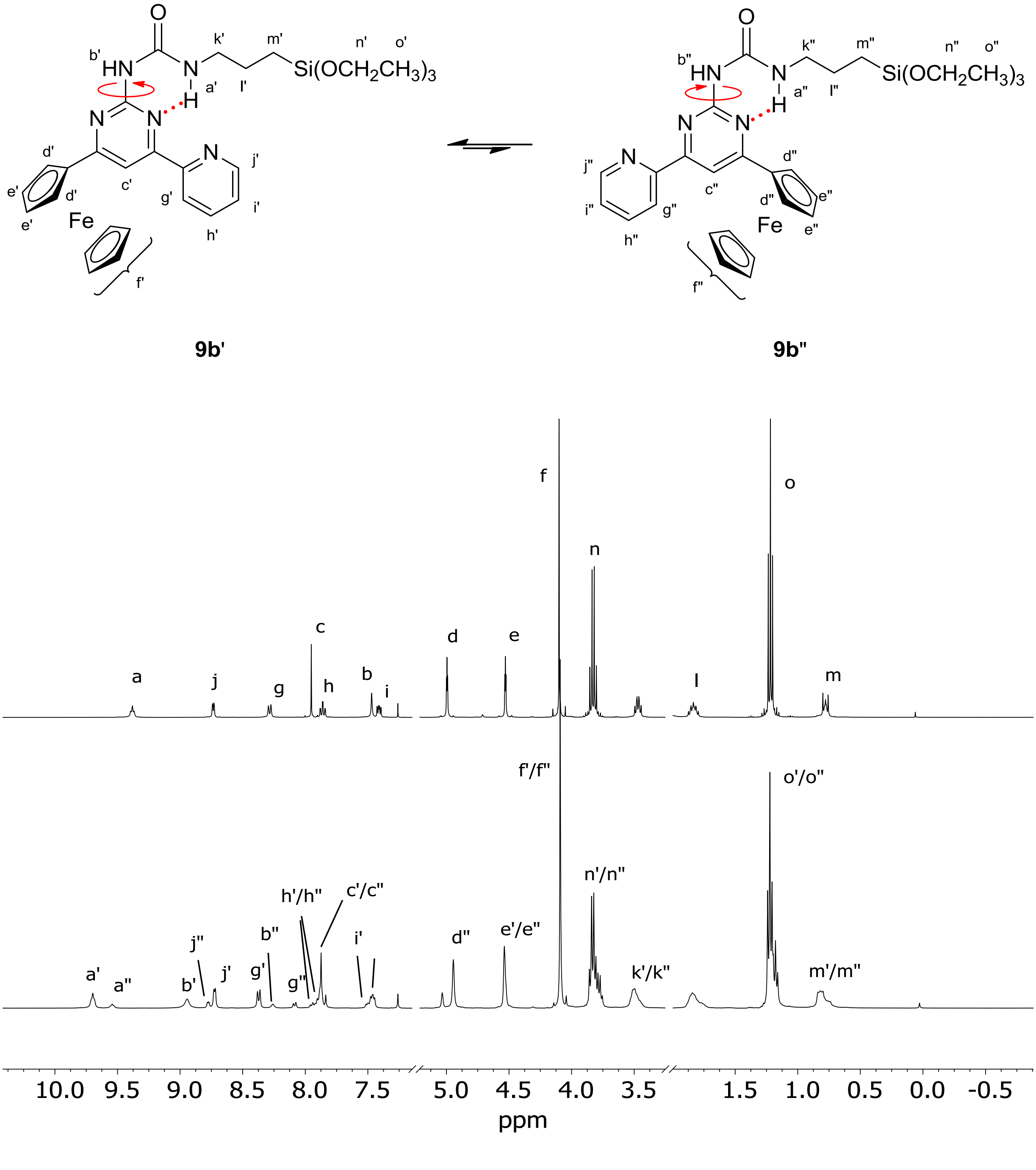

Figure S3. ^{1}H NMR spectra of **9b** at 300 K (top) and **9b**'/**9b**" at 209 K (bottom) ($CDCl_3$, 173 mM **9b**, 400 MHz).

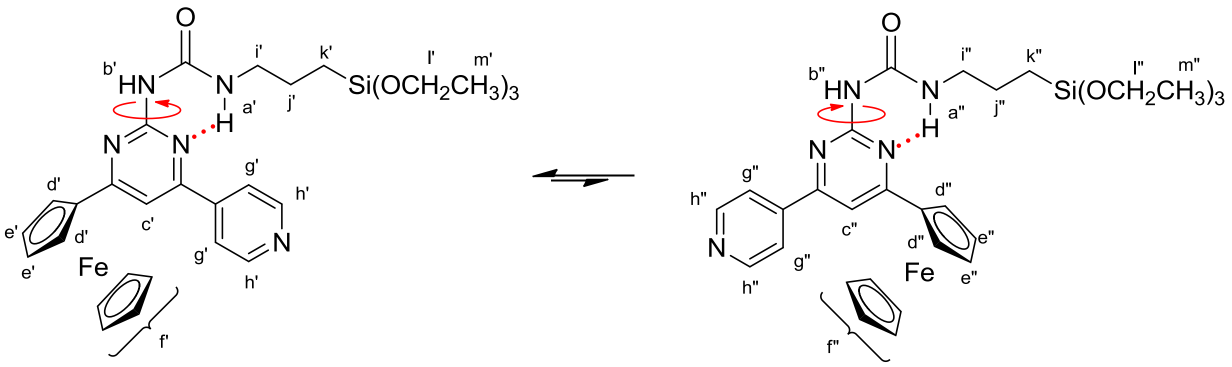

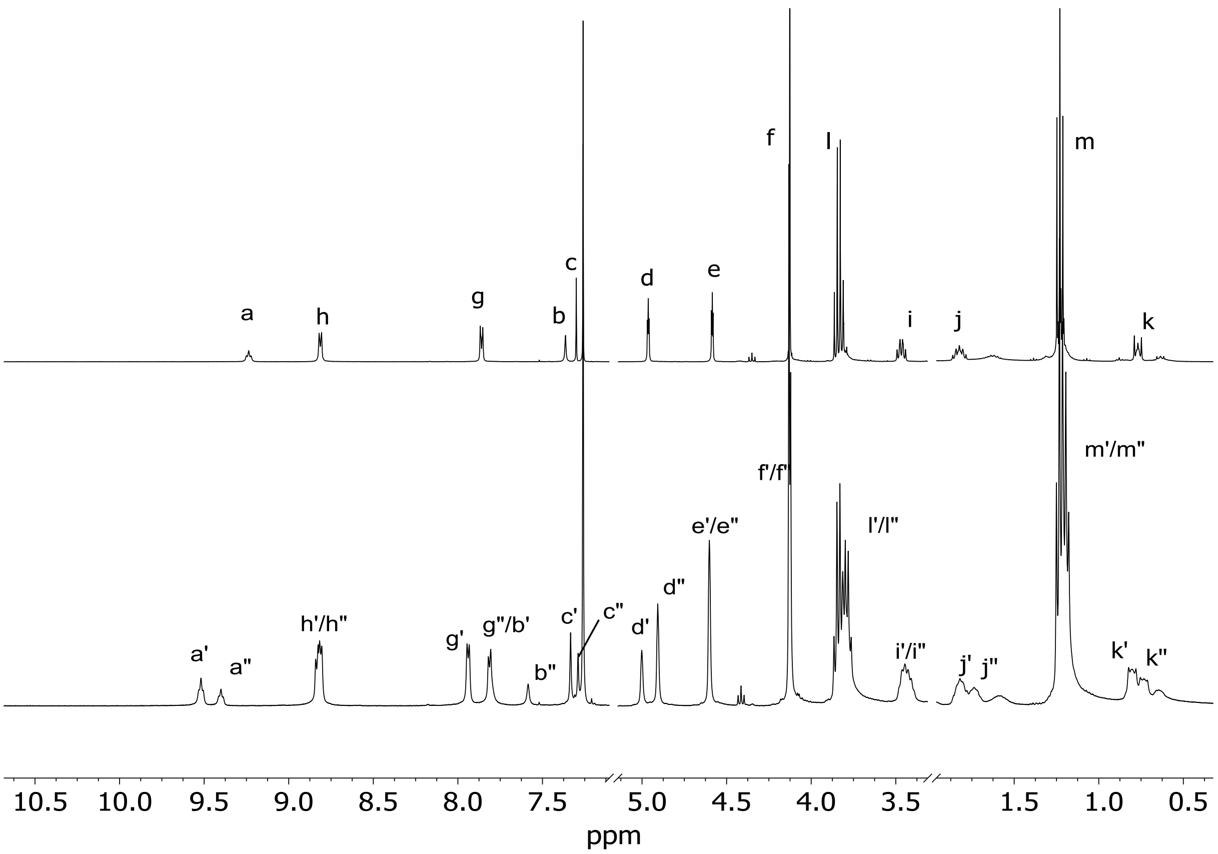

Figure S4. ^{1}H NMR spectra of **9c** at 300 K (top) and **9c**'/**9c**" at 209 K (bottom) ($CDCl_3$, 50 mM **9c**, 400 MHz).

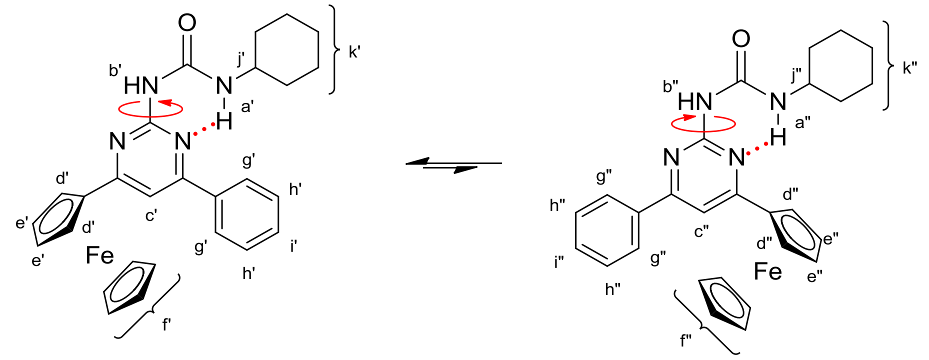

11' **11"**

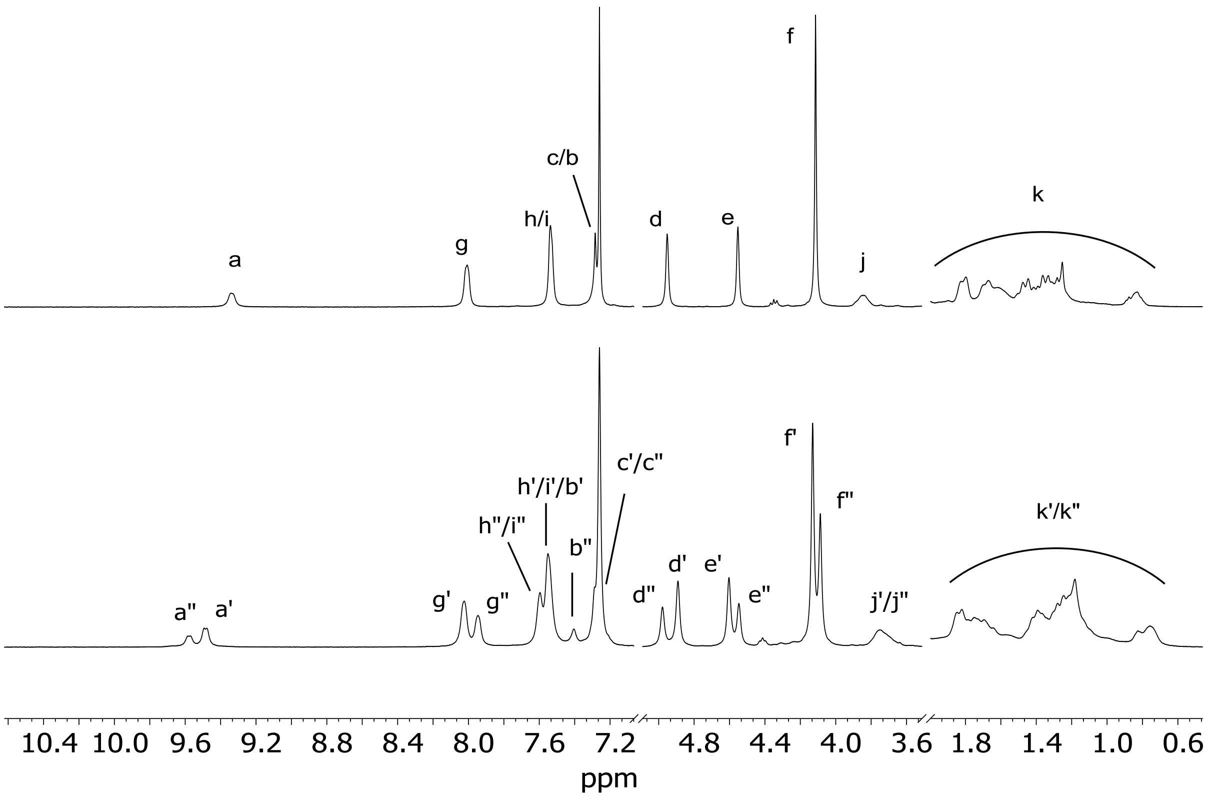

Figure S5. ^{1}H NMR spectra of **11** at 300 K (top) and **11**′/**11**″ at 209 K (bottom) ($CDCl_3$, 10 mM **11**, 400 MHz).

Table S1. Reaction rate constants and parameters obtained by lineshape fitting of low temperature NMR spectra of **10**.

T (K)	$10^3/T$ (K^{-1})	k (Hz)	$10^3 \cdot k/T$ ($Hz \cdot K^{-1}$)	ln(k/T)
209	4.785	0.473	2.262	−6.091
218	4.587	1.974	9.057	−4.704
227	4.405	8.230	36.257	−3.317
236	4.237	23.615	100.062	−2.302
245	4.082	70.229	286.647	−1.250

Table S2. Reaction rate constants and parameters obtained by lineshape fitting of low temperature NMR spectra of **11**.

T (K)	$10^3/T$ (K^{-1})	k (Hz)	$10^3 \cdot k/T$ ($Hz \cdot K^{-1}$)	ln(k/T)
209	4.785	2.382	11.396	−4.474
218	4.587	7.559	34.675	−3.362
227	4.405	16.858	74.265	−2.600
236	4.237	50.000	211.864	−1.552
245	4.082	99.500	406.122	−0.901

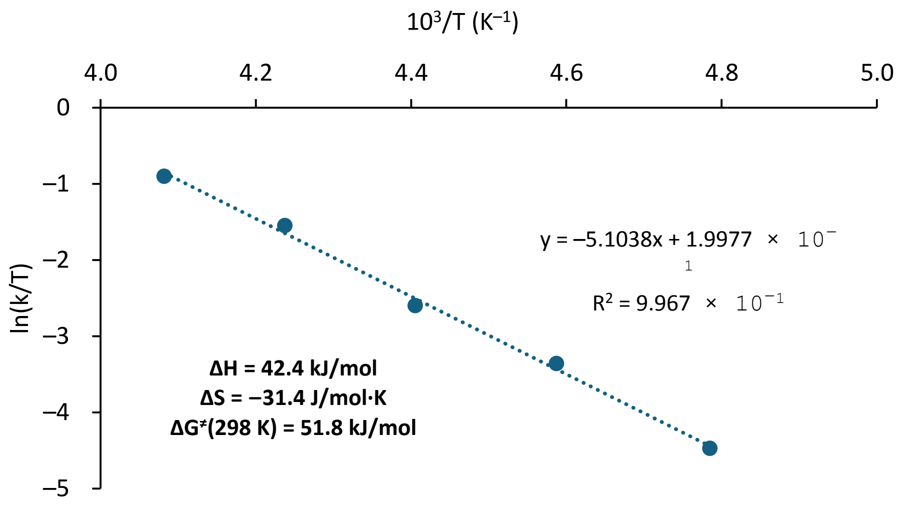

Figure S6. Eyring-plot for the rotational conformation change reaction of **11**.

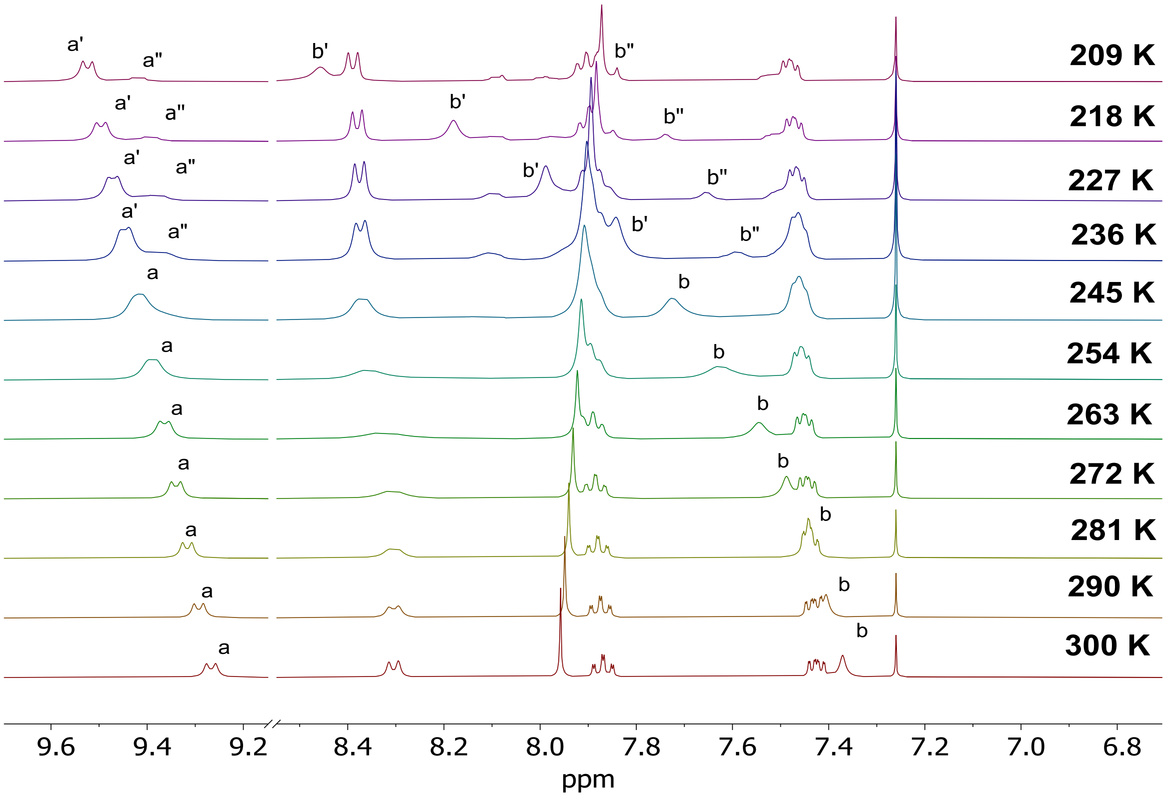

Figure S7. Temperature dependence of the chemical shift of protons H_a and H_b of **10** ($CDCl_3$, 78 mM **10**, 400 MHz).

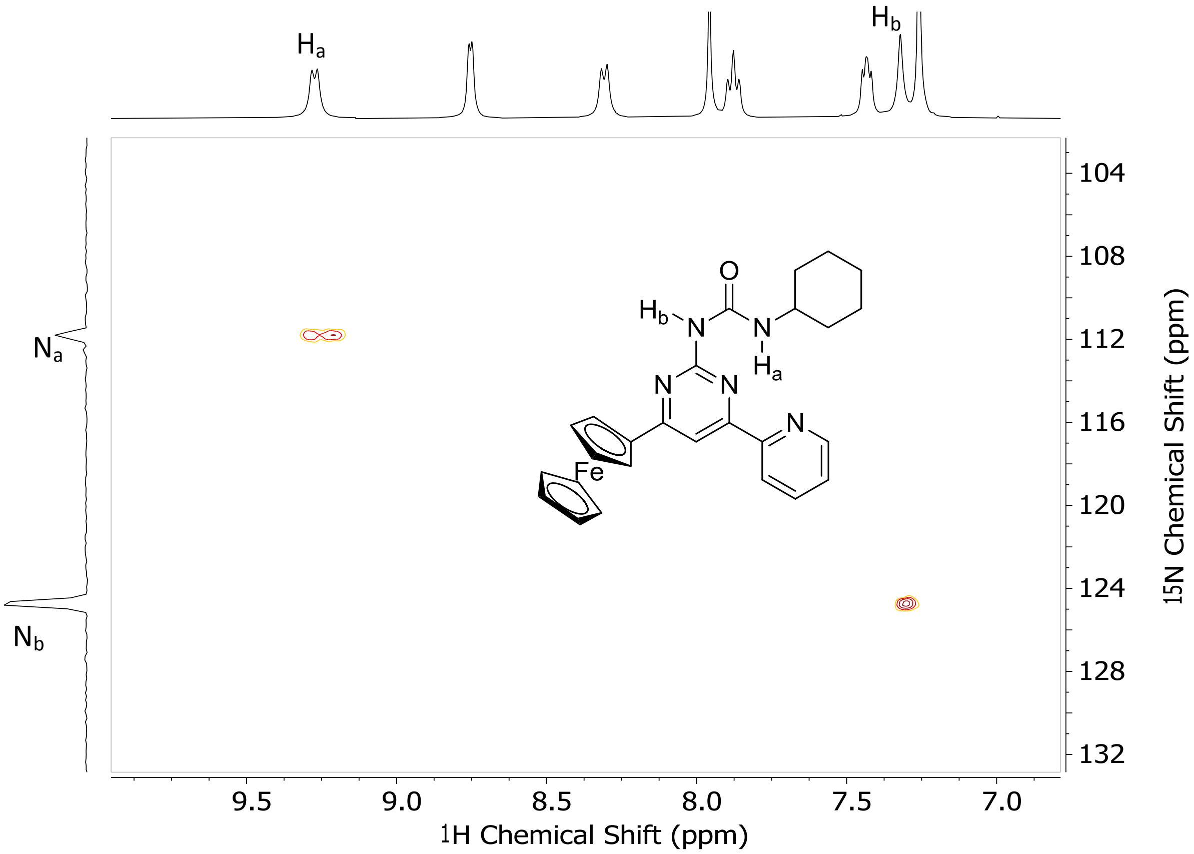

Figure S8. ^{1}H–^{15}N HSQC spectrum of compound **10** ($CDCl_3$, 299 K, 400 MHz).

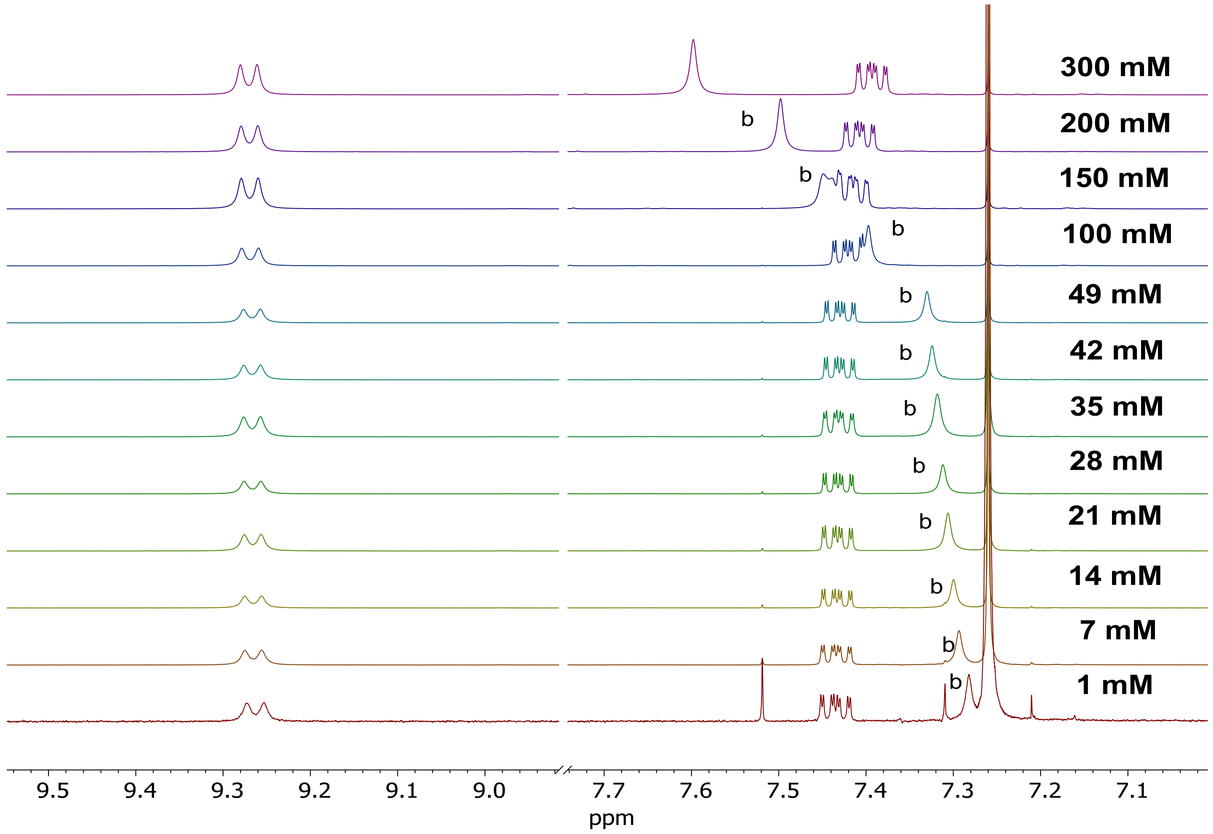

Figure S9. Concentration dependence of the chemical shift of protons H_a and H_b of **10** ($CDCl_3$, 299 K, 400 MHz).

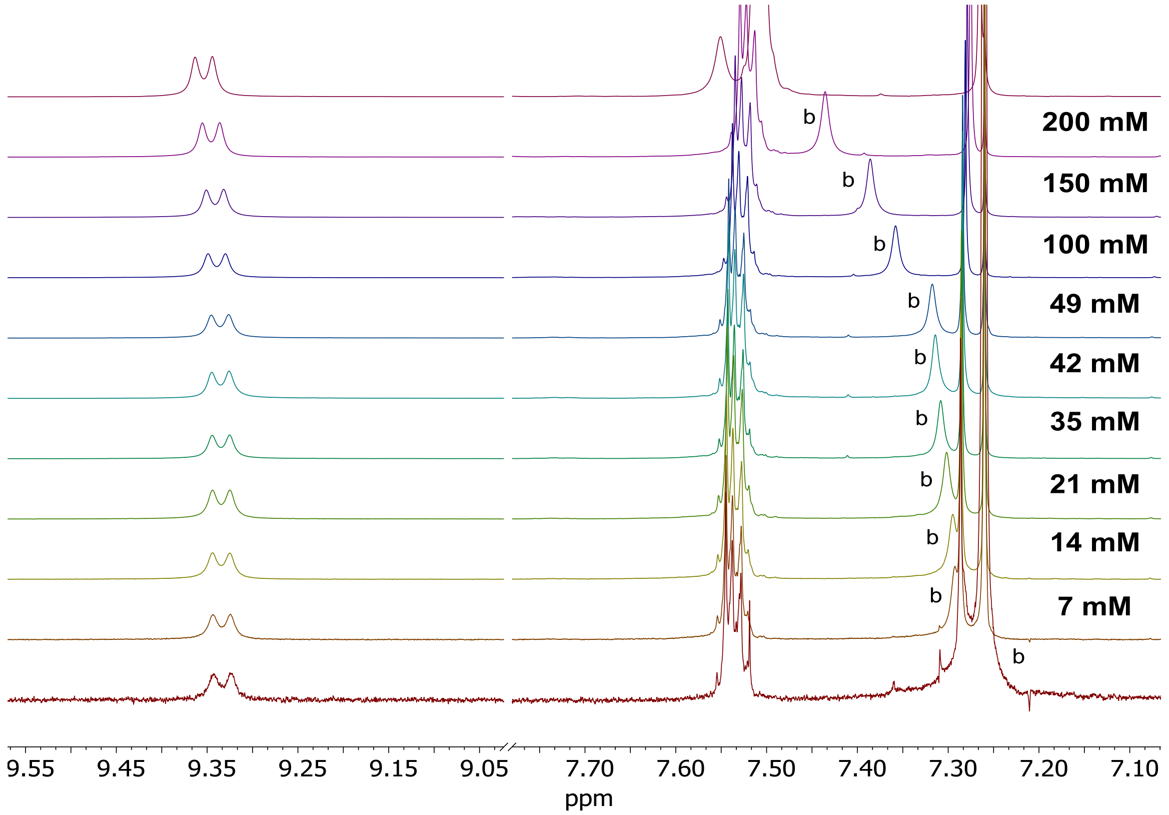

Figure S10. Concentration dependence of the chemical shift of protons H_a and H_b of **11** ($CDCl_3$, 297 K, 400 MHz).

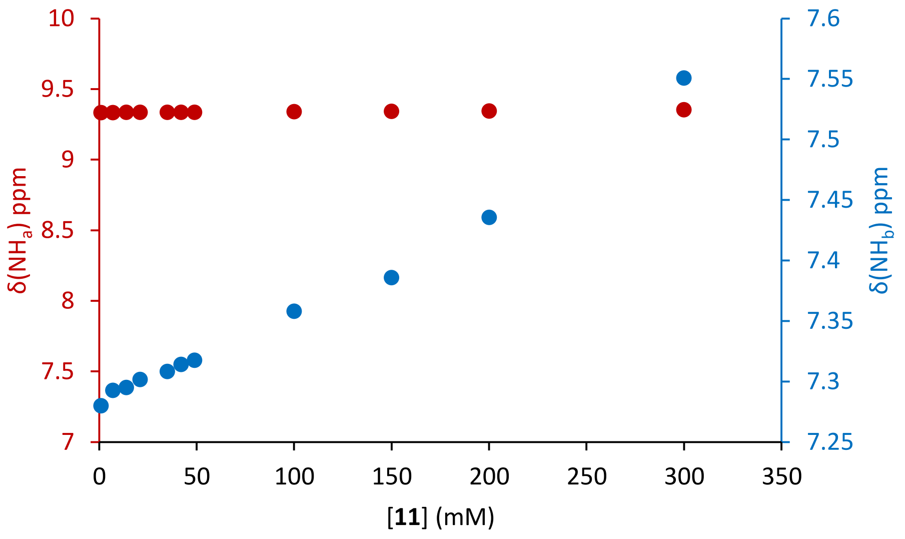

Figure S11. Change of chemical shift of urea protons with the concentration of **11** ($CDCl_3$, 297 K, 400 MHz).

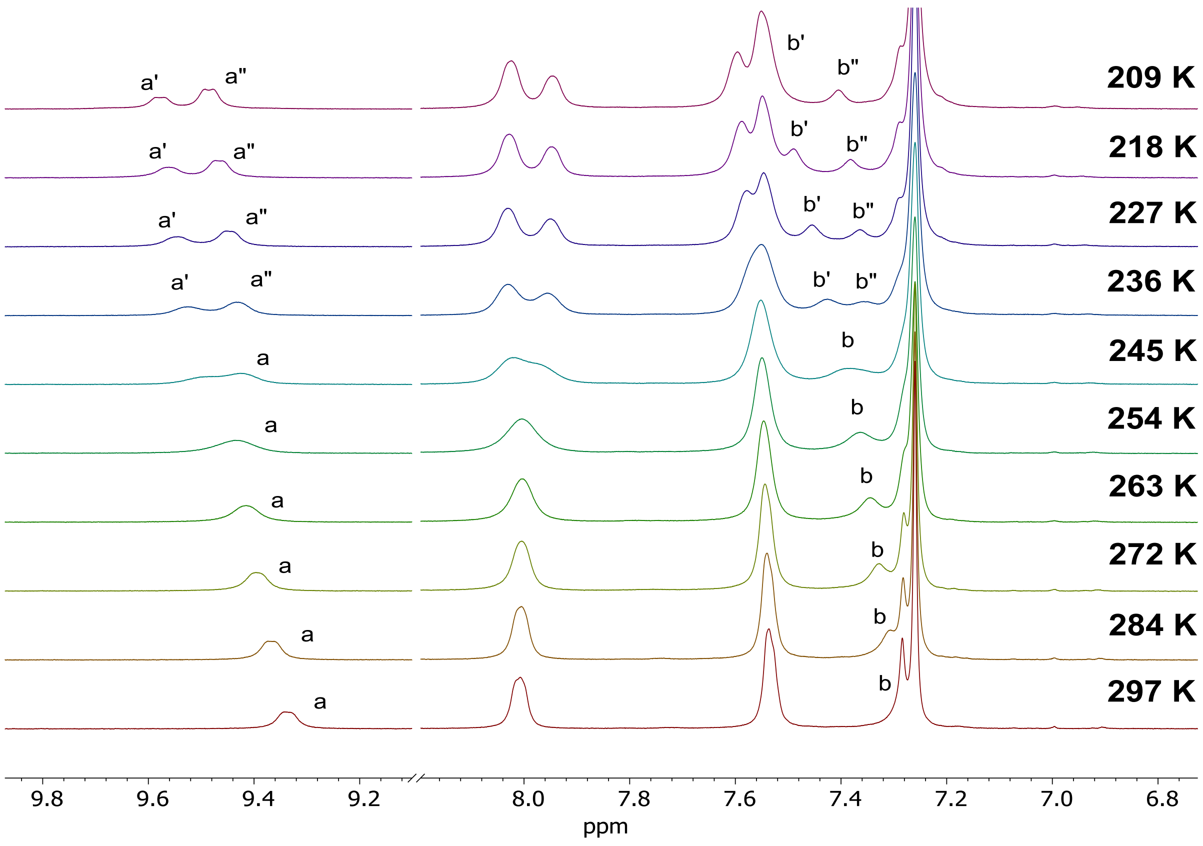

Figure S12. Temperature dependence of the chemical shift of protons H_a and H_b of **11** ($CDCl_3$, 10 mM **11**, 400 MHz).

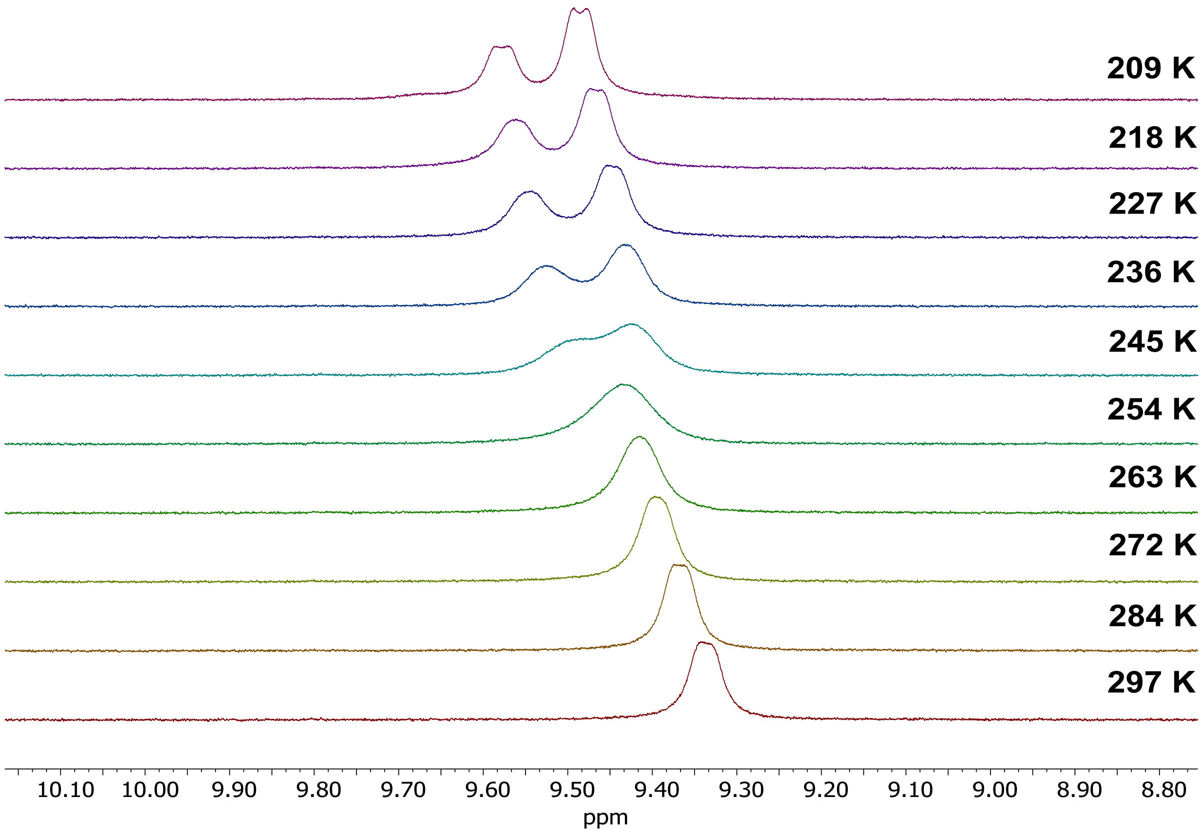

Figure S13. Coalescence of the signal of proton NH_a of **11** ($CDCl_3$, 10 mM **11**, 400 MHz).

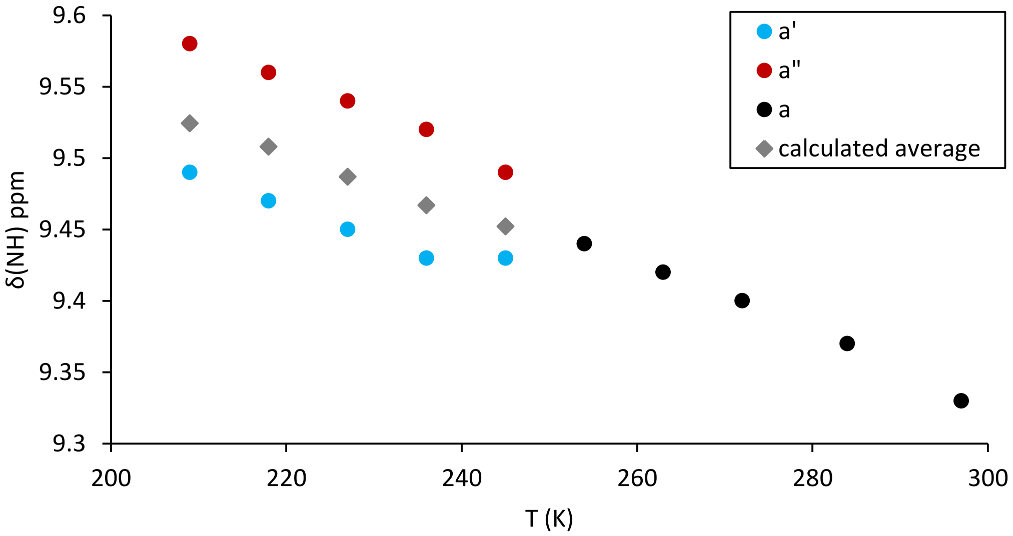

Figure S14. Temperature dependence of the chemical shift of NH_a proton of **11** ($CDCl_3$, 10 mM **11**, 400 MHz). Calculated average values at each temperature are given as the mole-fraction-weighted sum of the chemical shifts of H_a' and H_a" protons of the conformers.

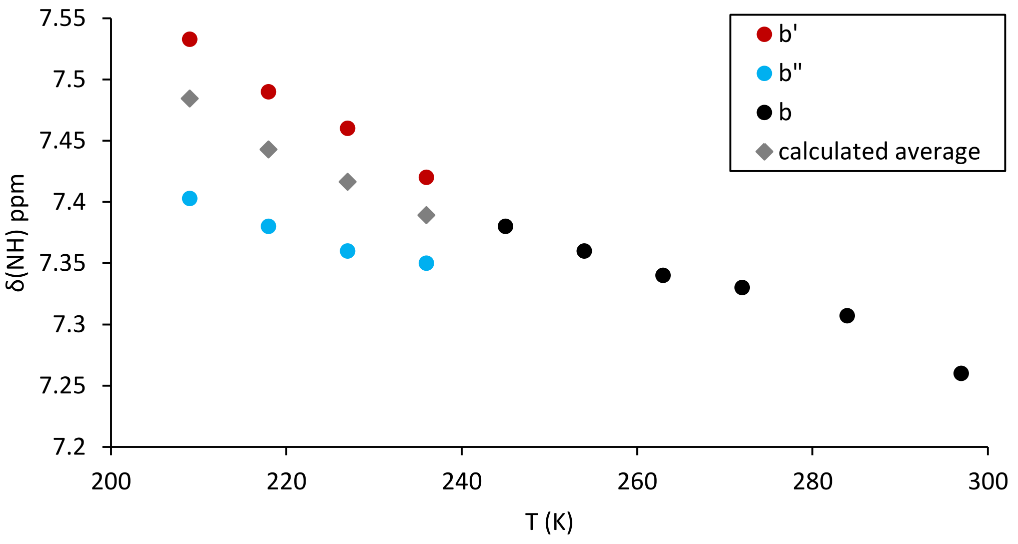

Figure S15. Temperature dependence of the chemical shift of NH_b proton of **11** ($CDCl_3$, 10 mM **11**, 400 MHz). Calculated average values at each temperature are given as the mole-fraction-weighted sum of the chemical shifts of H_b' and H_b'' protons of the conformers.

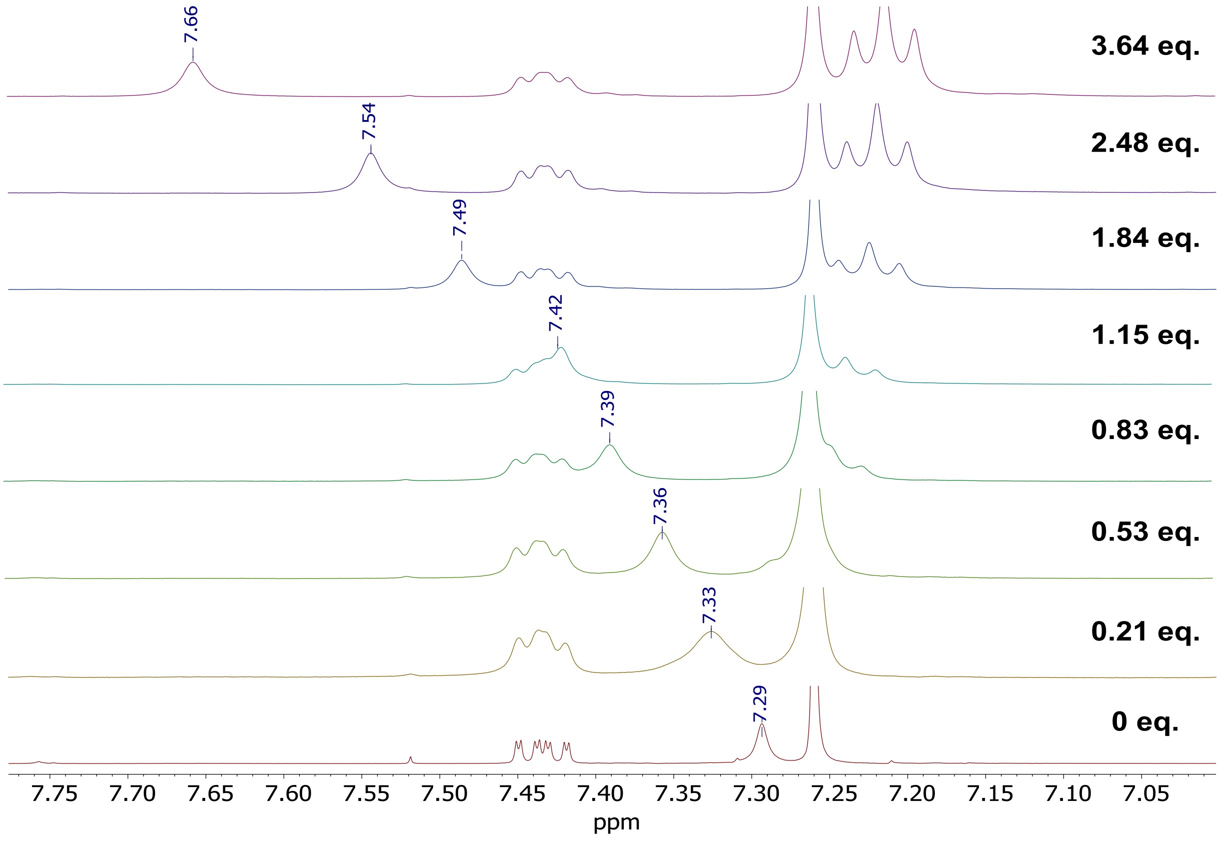

Figure S16. Changes in the chemical shift of NH_b proton of **10** upon titration with **2** ($CDCl_3$, 10 mM **10**, 298 K, 400 MHz). Equivalents of **2** were calculated from the integrals of signals of **10** and **2**.

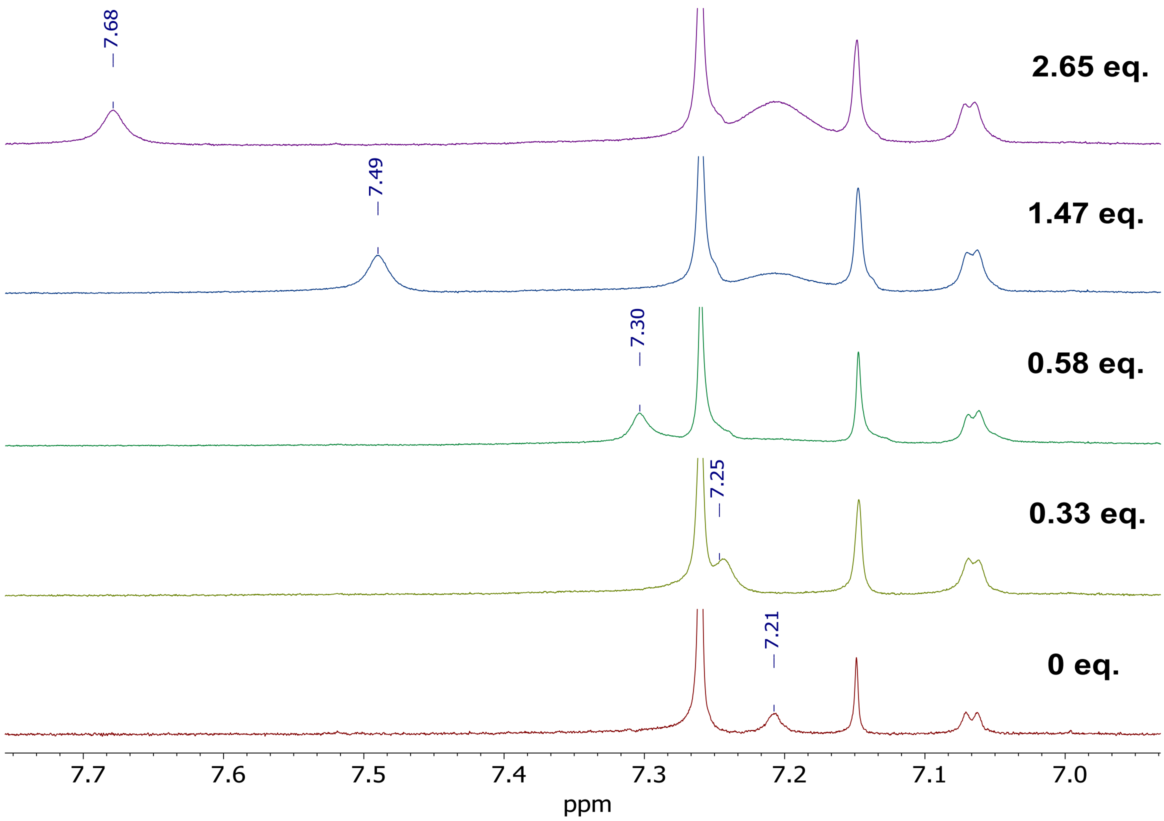

Figure S17. Changes in the chemical shift of proton NH_b of **9a** upon titration with **2** ($CDCl_3$, 5 mM **9a**, 300 K, 400 MHz). Equivalents of **2** were calculated from the integrals of signals of **9a** and **2**.

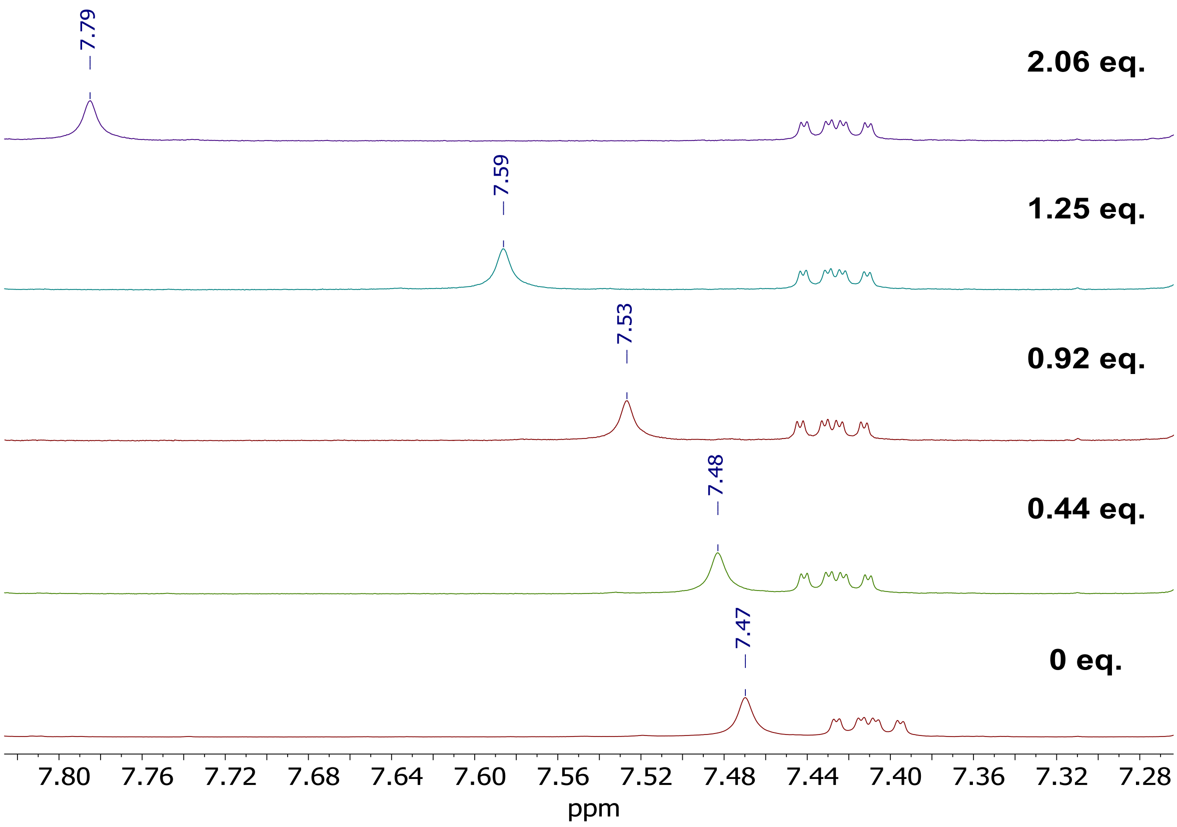

Figure S18. Changes in the chemical shift of NH_b proton of **9b** upon titration with **2** ($CDCl_3$, 173 mM **9b**, 300 K, 400 MHz). Equivalents of **2** were calculated from the integrals of signals of **9b** and **2**.

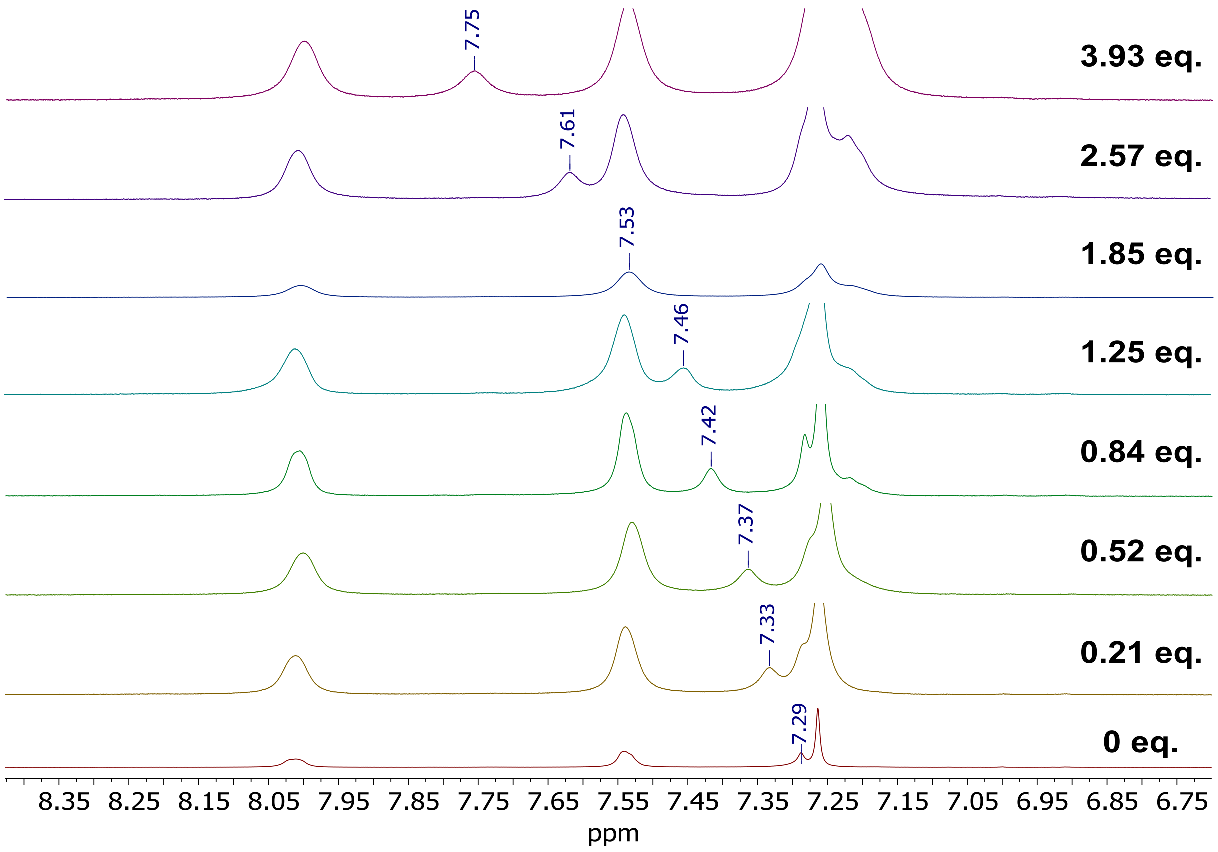

Figure S19. Changes in the chemical shift of NH_b proton of **11** upon titration with **2** ($CDCl_3$, 10 mM **11**, 297 K, 400 MHz). Equivalents of **2** were calculated from the integrals of signals of **11** and **2**.

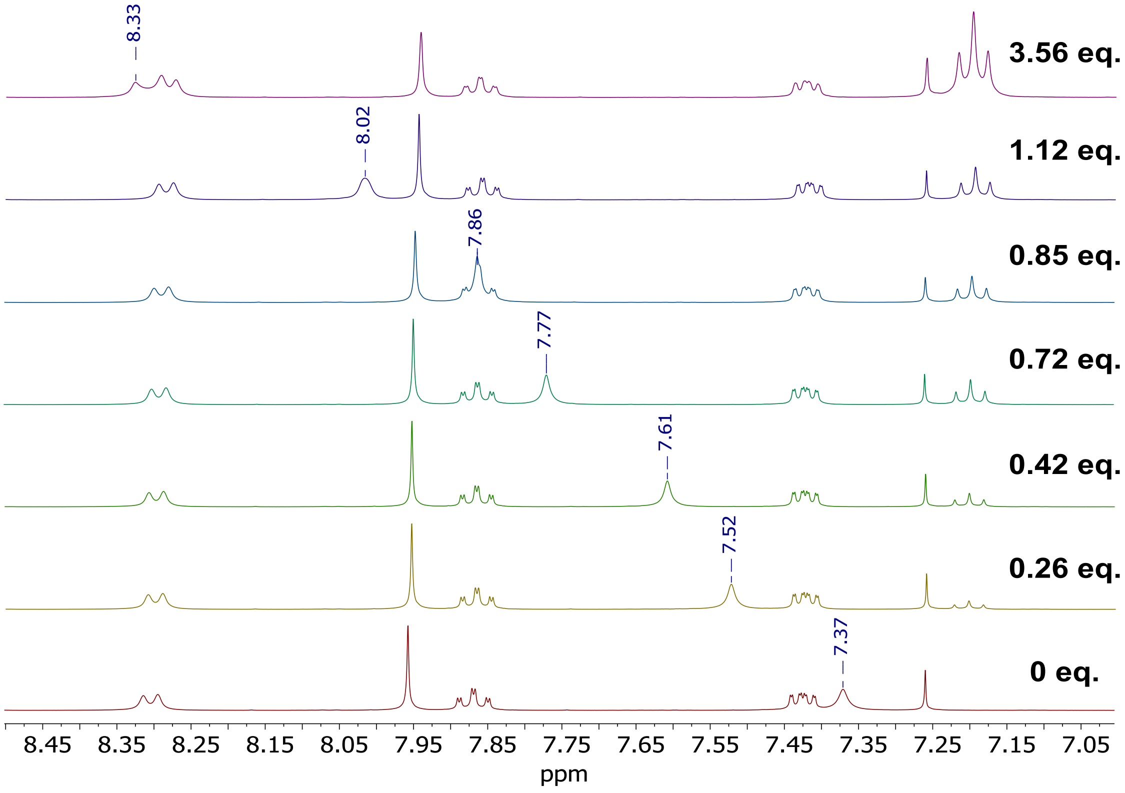

Figure S20. Change in the chemical shift of proton NH_b of **10** at 300 K upon titration with **2** ($CDCl_3$, 78 mM **10**, 400 MHz). Equivalents of **2** were calculated from the integrals of signals of **10** and **2**.

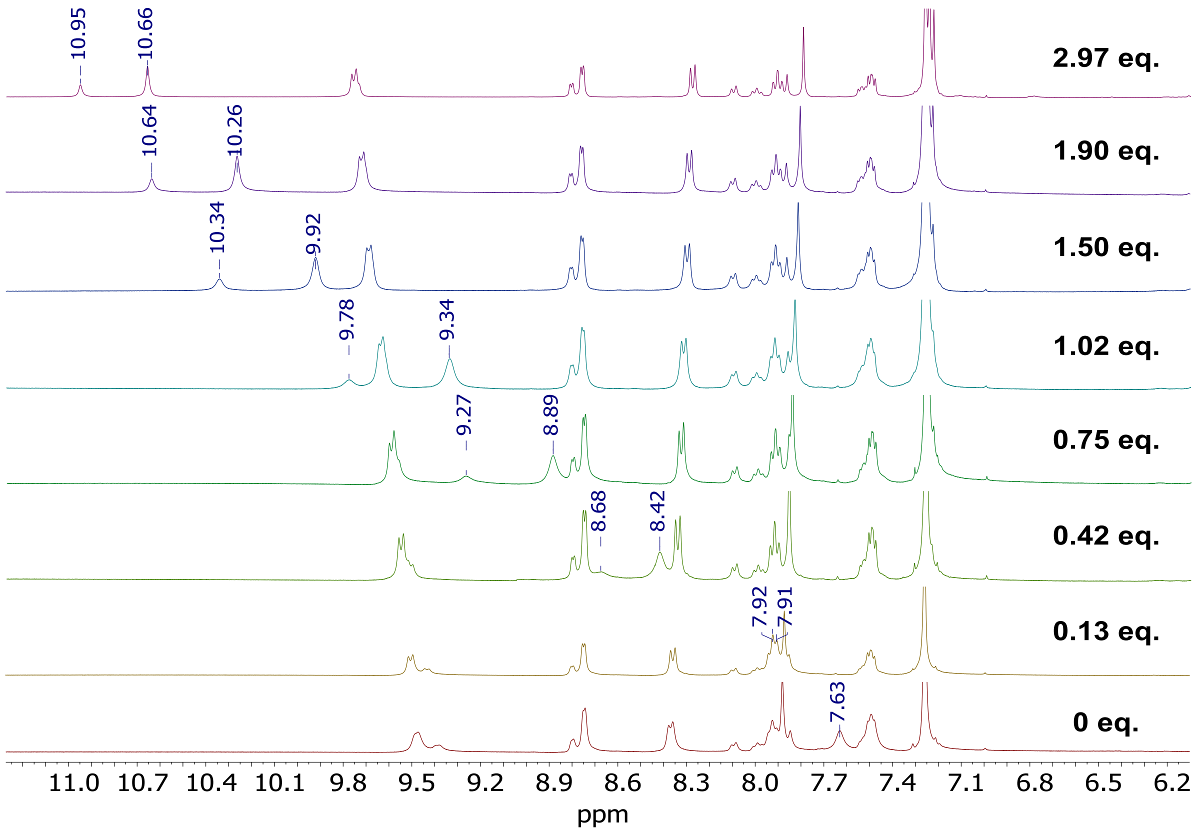

Figure S21. Chemical shift of proton NH_b of **10**′ and **10**″ at 209 K upon titration with **2** ($CDCl_3$, 10 mM **10**, 400 MHz). **10**″/**10**′ ratios were gained from lineshape fitting of H_j′ and H_j″ signals in all cases due to overlapping. Equivalents of **2** were calculated from the integrals of signals of **10** and **2**.

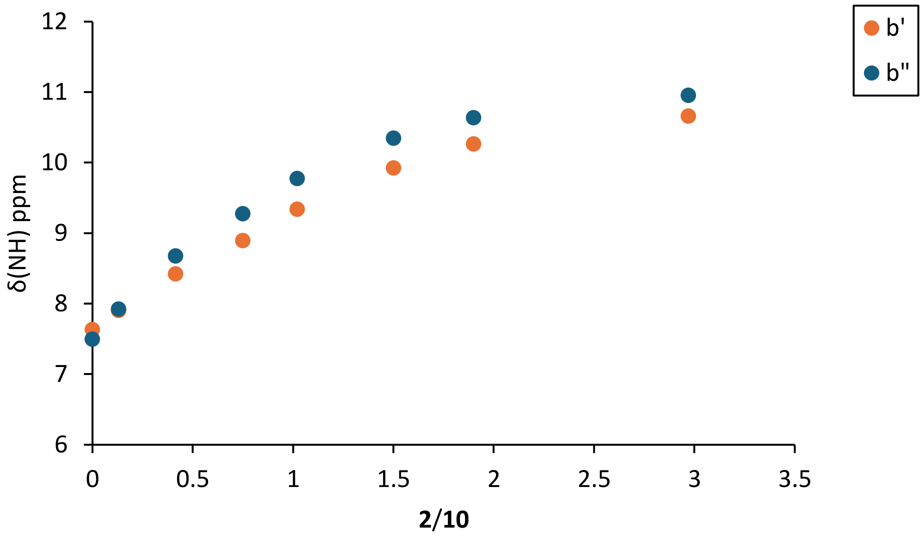

Figure S22. Effect of the addition of **2** on the chemical shift of proton NH_b of conformers of **10** at 209 K ($CDCl_3$, 10 mM **10**, 400 MHz).

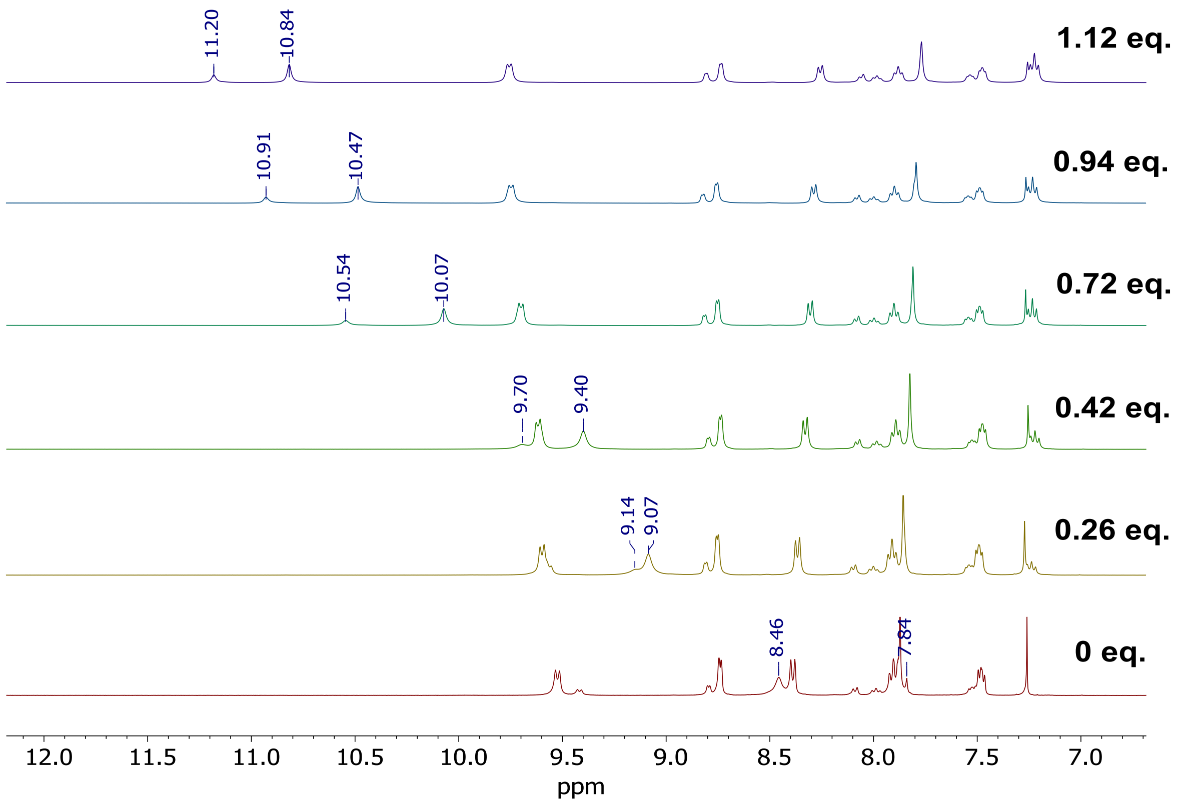

Figure S23.Chemical shifts of protons NH_b of **10’** and **10”** at 209 K upon titration with **2** ($CDCl_3$, 78 mM **10**, 400 MHz). **10**”/**10**’ ratios were gained from lineshape fitting of H_j’ and H_j” signals in all cases due to overlapping. Equivalents of **2** added were calculated from the integrals of signals of **10** and **2**.

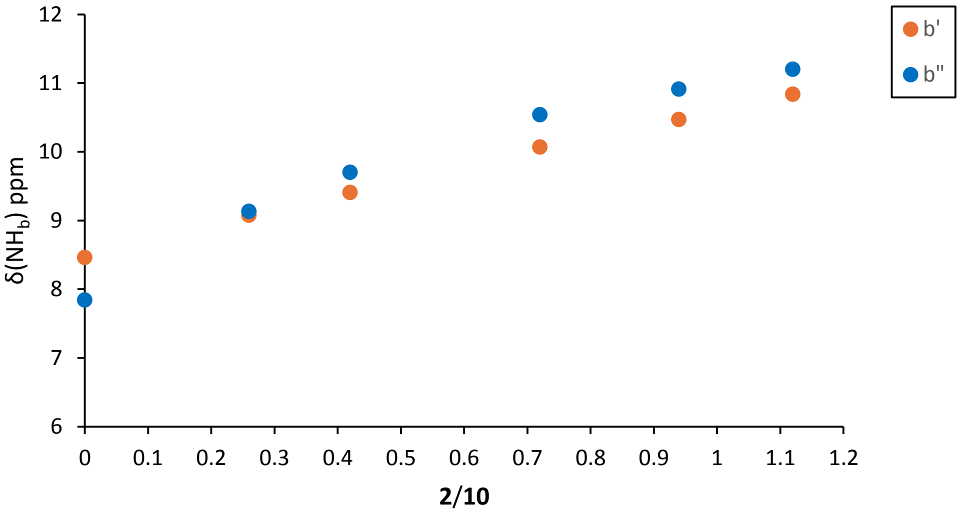

Figure S24. Effect of **2** on the chemical shift of proton NH_b of conformers of **10** at 209 K ($CDCl_3$, 78 mM **10**, 400 MHz).

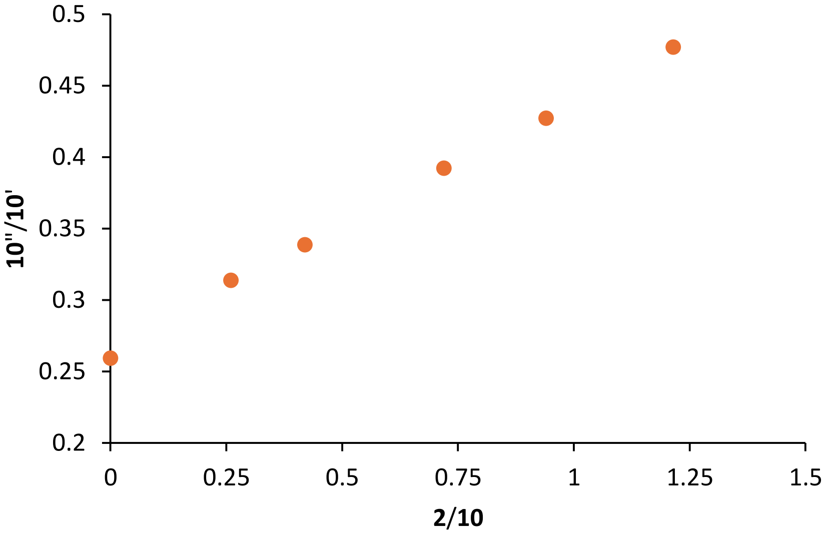

Figure S25. Change in the ratio of **10**”/**10**’ conformers upon titration with **2** ($CDCl_3$,78 mM **10**, 209 K, 400 MHz).

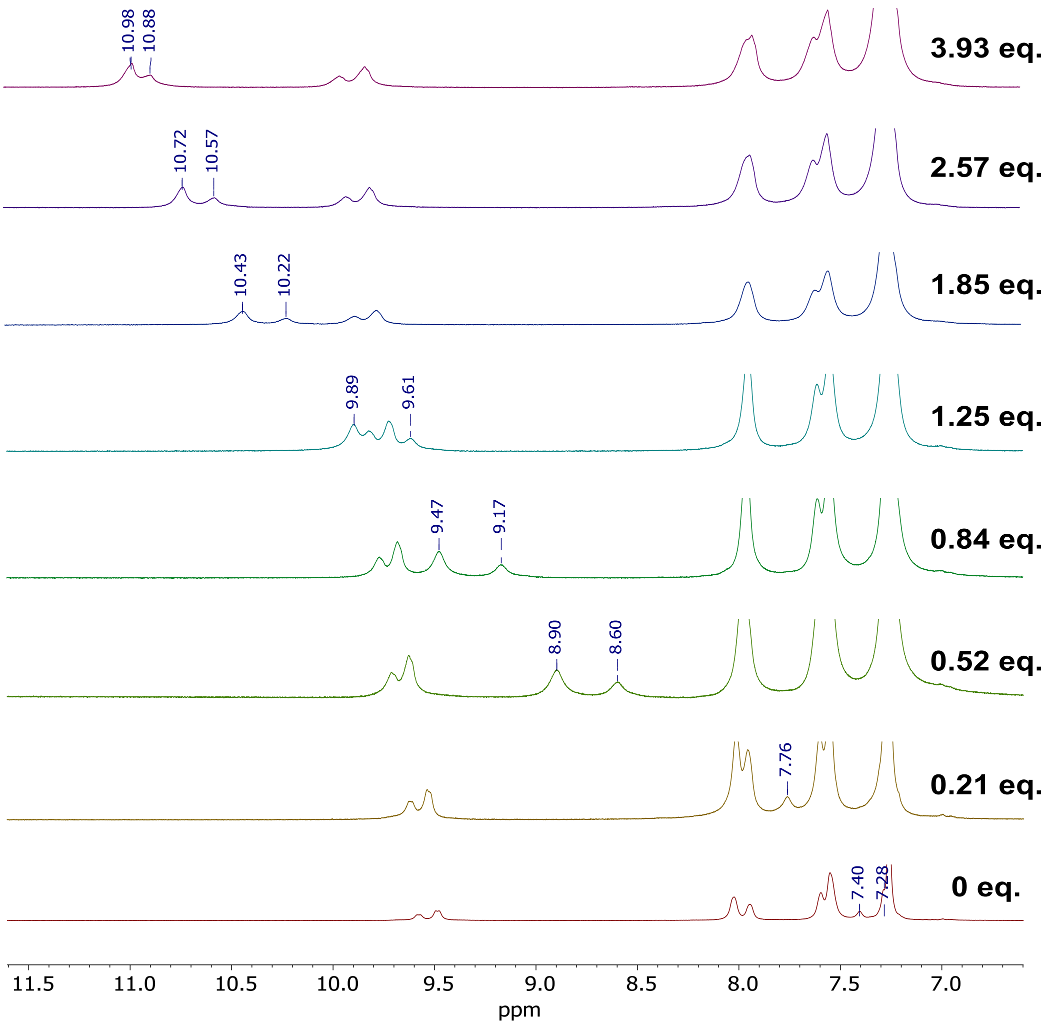

Figure S26. Chemical shift of proton NH_b of **11**’ and **11**” at 209 K upon titration with **2** ($CDCl_3$, 10 mM **11**, 400 MHz). **11**”/**11**’ ratios were gained from lineshape fitting of H_b’ and H_b” signals in all cases due to overlapping. Equivalents of **2** were calculated from the integrals of signals of **11** and **2**.

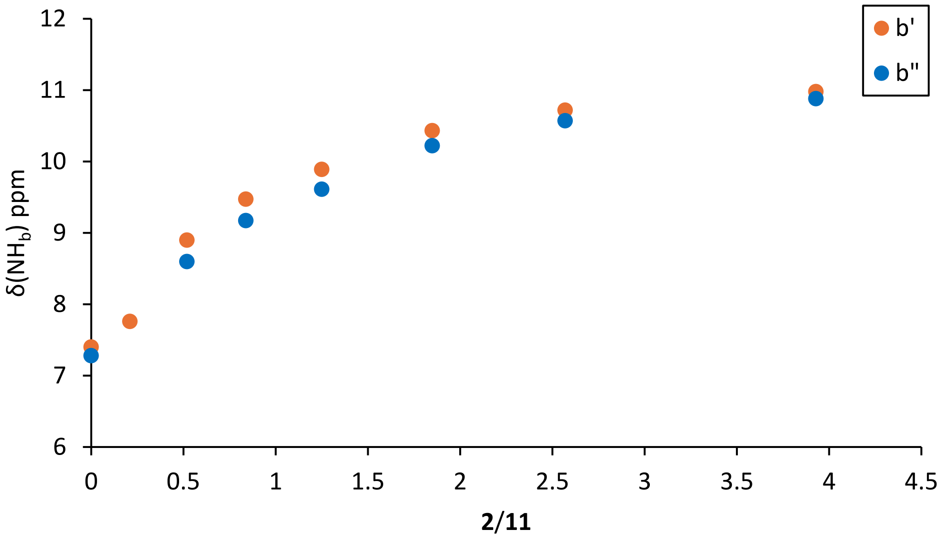

Figure S27. Effect of **2** on the chemical shift of NH_b proton of conformers of **11** at 209 K ($CDCl_3$, 10 mM **11**, 400 MHz).

S2. Supplementary electrochemical data

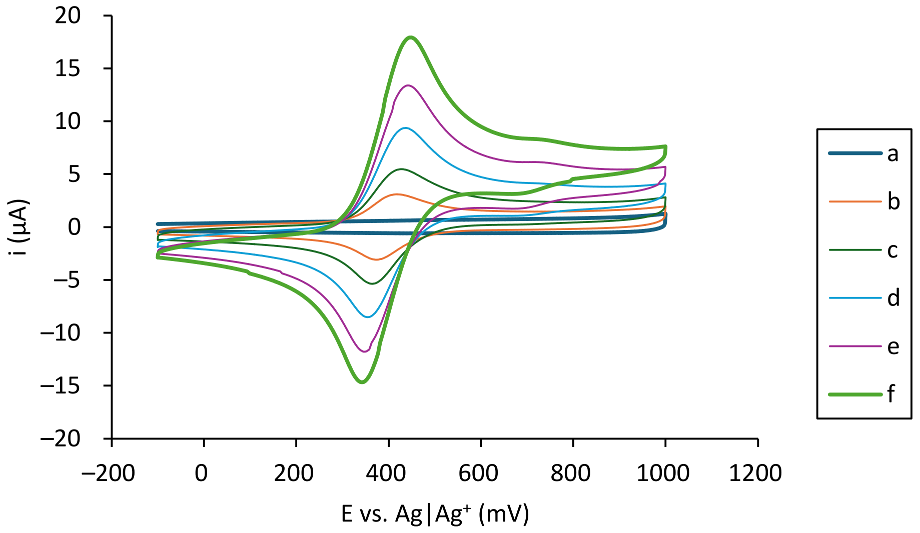

Figure S28. Cyclic voltammograms of **10** of different concentration (glassy carbon working electrode (OD = 3 mm), Ag|Ag^+ reference electrode, Pt-wire counter electrode, 0.1 M [*n*Bu_4N][PF_6] supporting electrolyte in CH_2Cl_2, 100 mVs^{-1} scan rate, concentration of **10** in mM: a: 0, b: 0.1, c: 0.25, d: 0. 5, e: 0.75, f: 1.0).

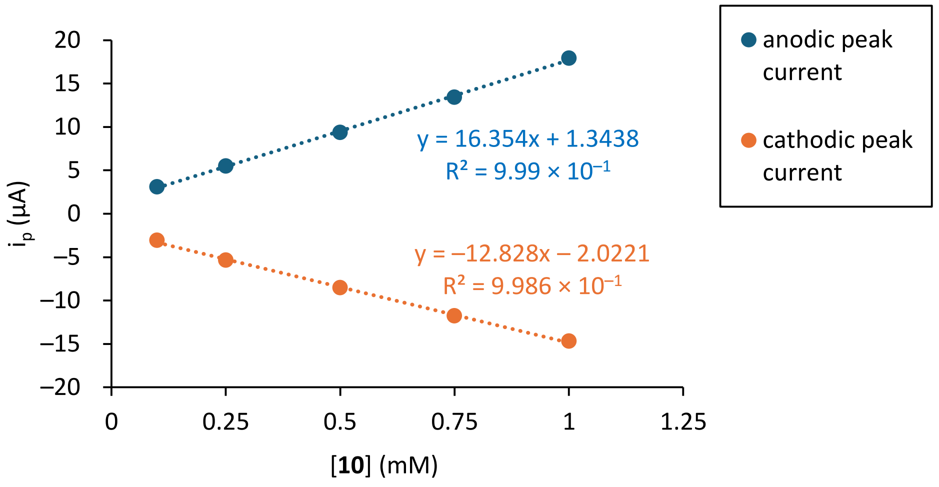

Figure S29. Randles-Ševčík plot of **10** at different concentrations (glassy carbon working electrode (OD = 3 mm), Ag|Ag^+ reference electrode, Pt-wire counter electrode, 0.1 M [*n*Bu_4N][PF_6] supporting electrolyte in CH_2Cl_2, 100 mVs^{-1} scan rate).

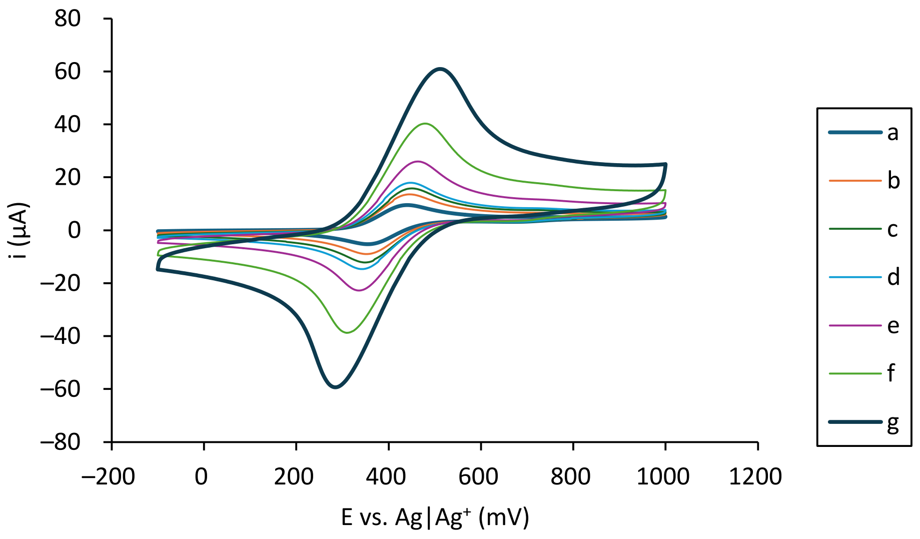

Figure S30. Cyclic voltammograms of **10** at different scan rates (glassy carbon working electrode (OD = 3 mm), Ag|Ag^+ reference electrode, Pt-wire counter electrode, 0.1 M [*n*Bu_4N][PF_6] supporting electrolyte in CH_2Cl_2, 1.0 mM **10**, scan rates in mVs^{-1}: a: 25, b: 50, c: 75, d: 100, e: 200, f: 500, g:1000).

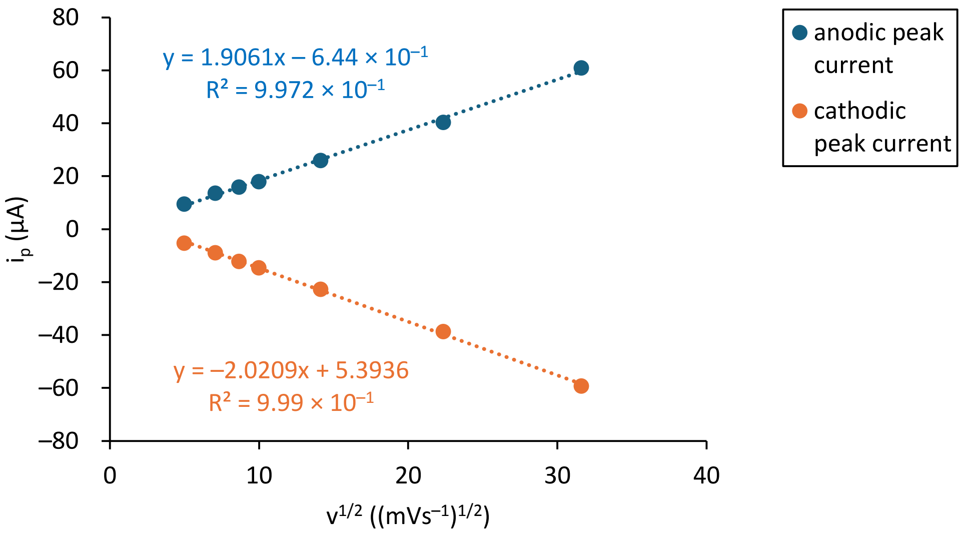

Figure S31. Randles-Ševčík plot of **10** at different scan rates (glassy carbon working electrode (OD = 3 mm), Ag|Ag^+ reference electrode, Pt-wire counter electrode, 0.1 M [*n*Bu_4N][PF_6] supporting electrolyte in CH_2Cl_2, 1 mM **10**).

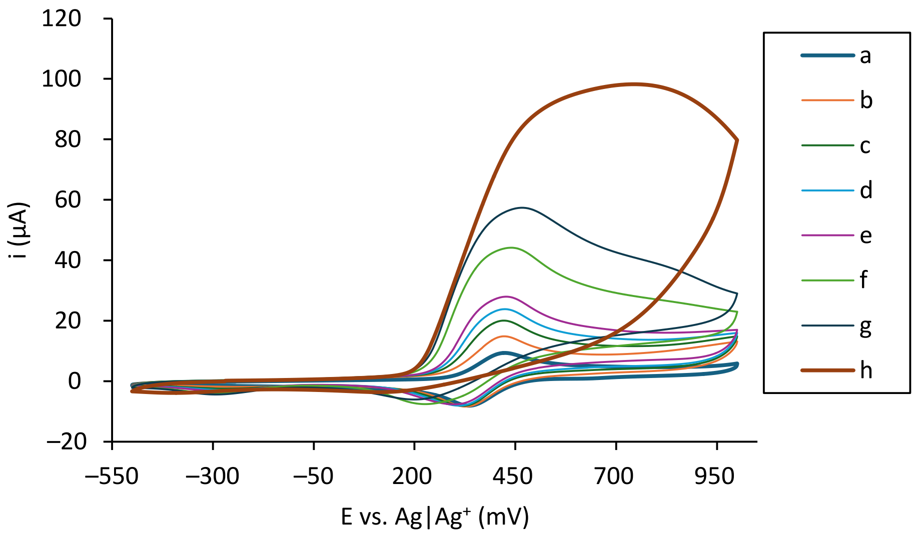

Figure S32. Cyclic voltammograms of **2**/**10** mixtures (0.56 mM **10** in CH_2Cl_2, glassy carbon working electrode (OD = 3 mm), Ag|Ag^+ reference electrode, Pt-wire counter electrode, 0.1 M [$n$$Bu_4$N][$PF_6$] supporting electrolyte, 100 mVs^{-1} scan rate, equivalents of **2**: a: 0, b: 0.5, c: 1.0, d: 1.5, e: 2.0, f: 4.0, g: 6.0, h: 20.0).

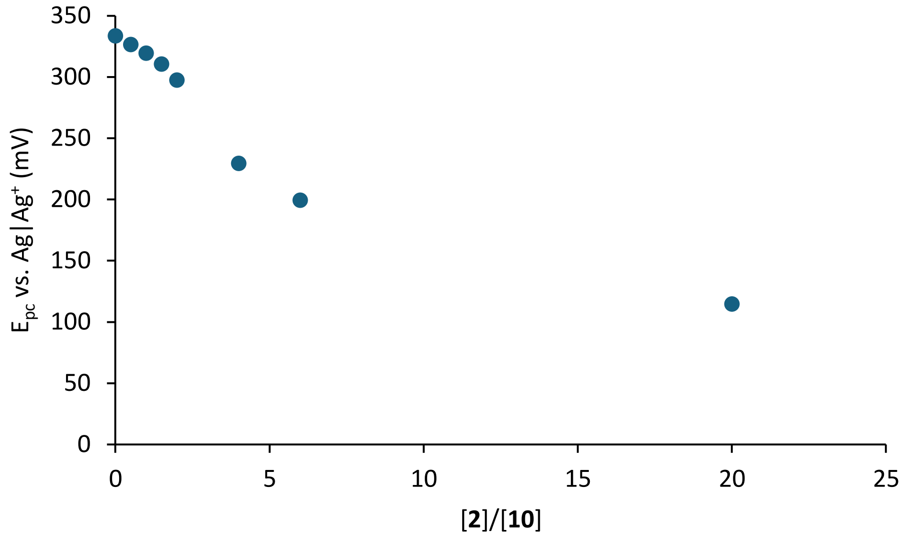

Figure S33. Change of the cathodic peak potential of **10** as a function of [**2**]/[**10**] ratio (0.56 mM **10** in CH_2Cl_2, glassy carbon working electrode (OD = 3 mm), Ag|Ag^+ reference electrode, Pt-wire counter electrode, 0.1 M [$n$$Bu_4$N][$PF_6$] supporting electrolyte, 100 mVs^{-1} scan rate).

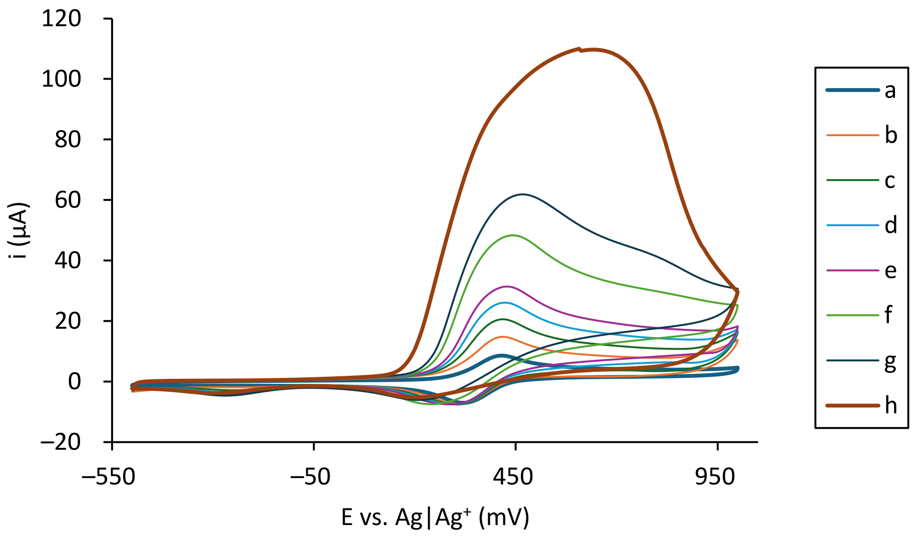

Figure S34. Cyclic voltammograms of **2**/**11** mixtures (0.64 mM **11** in CH_2Cl_2, glassy carbon working electrode (OD = 3 mm), Ag|Ag^+ reference electrode, Pt-wire counter electrode, 0.1 M [*n*Bu_4N][PF_6] supporting electrolyte, 100 mVs^{-1} scan rate, equivalents of **2**: a: 0, b: 0.5, c: 1.0, d: 1.5, e: 2.0, f: 4.0, g: 6.0, h: 20.0).

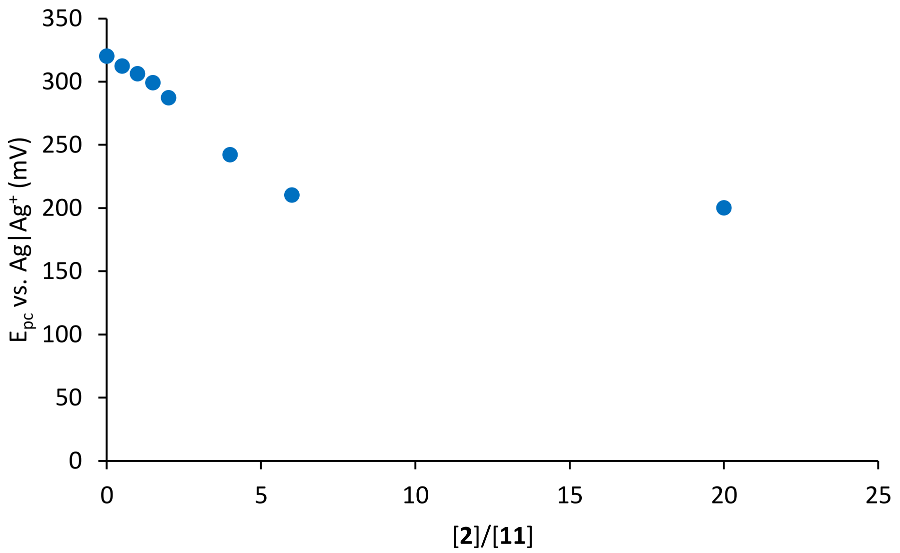

Figure S35. Change of the redox potentials of **11** as a function of **2**/**11** ratio (0.64 mM **11**, glassy carbon working electrode, Ag|Ag^+ reference electrode, Pt-wire counter electrode, 0.1 M [*n*Bu_4N][PF_6] supporting electrolyte in CH_2Cl_2, 100 mVs^{-1} scan rate).

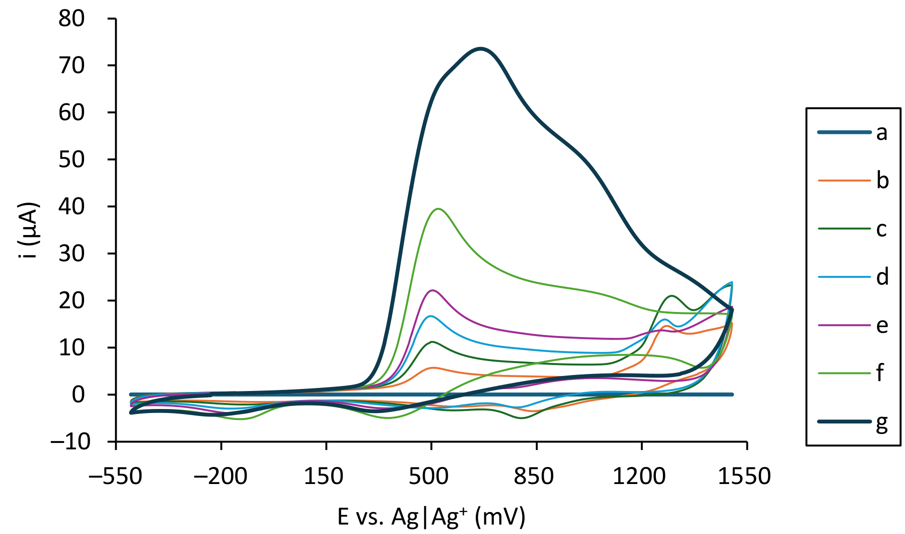

Figure S36. Cyclic voltammograms of **2** of different concentration (glassy carbon working electrode (OD = 3 mm), Ag|Ag$^+$ reference electrode, Pt-wire counter electrode, 0.1 M [nBu$_4$N][PF$_6$] supporting electrolyte in CH_2Cl_2, 100 mVs^{-1} scan rate, concentration of **2** in mM: a: 0, b: 0.25, c: 0.5, d: 0.75, e: 1.0, f: 2.0, g: 5.0).

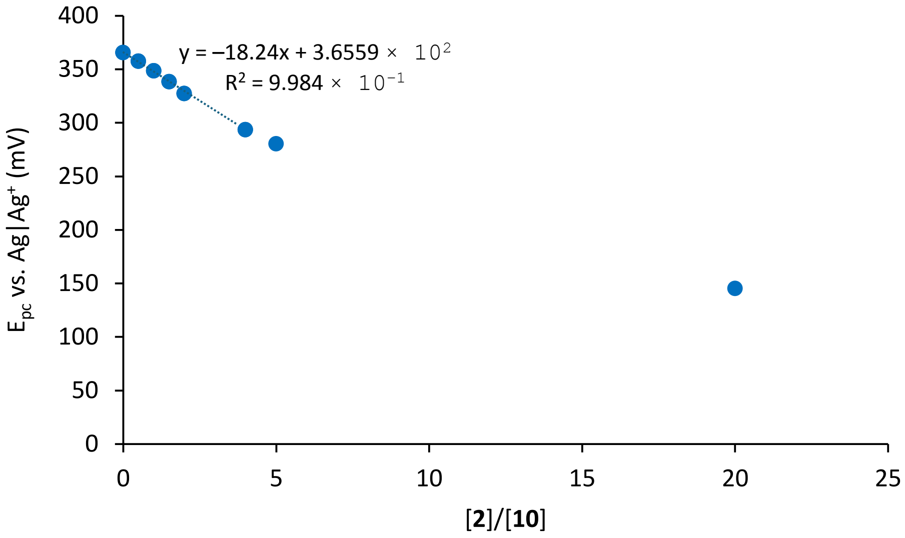

Figure S37. Slope of the cathodic peak potential of LSV response curves of **10** vs. [**2**]/[**10**] ratio at low ratios (0.56 mM **10** in CH_2Cl_2, glassy carbon working electrode (OD = 3 mm), Ag|Ag$^+$ reference electrode, Pt-wire counter electrode, 0.1 M [nBu$_4$N][PF$_6$] supporting electrolyte, 100 mVs^{-1} scan rate).

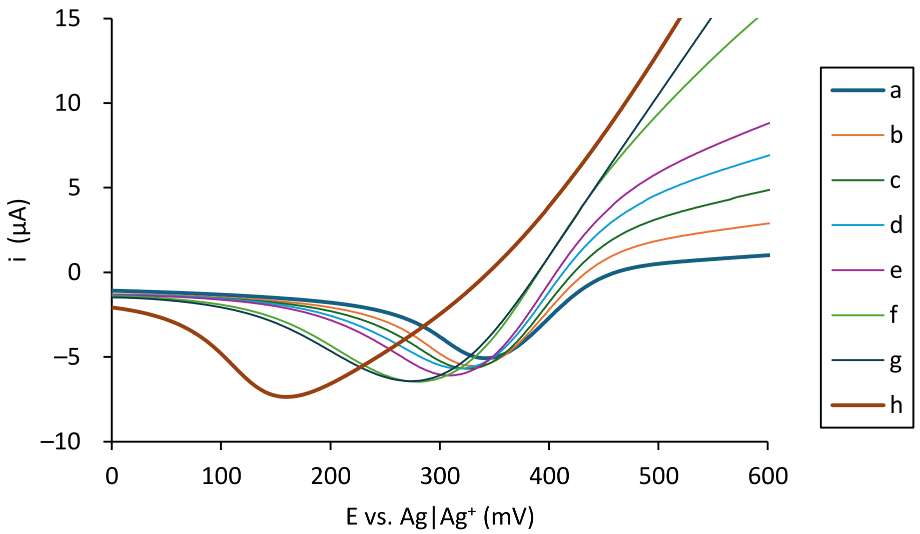

Figure S38. LSV response of **2**/**11** mixtures (0.56 mM **11** in CH_2Cl_2, glassy carbon working electrode (OD = 3 mm), Ag|Ag$^+$ reference electrode, Pt-wire counter electrode, 0.1 M [nBu$_4$N][PF$_6$] supporting electrolyte, 100 mVs^{-1} scan rate, equivalents of **2**: a: 0, b: 0.5, c: 1.0, d: 1.5, e: 2.0, f: 4.0, g: 5.0, h: 20.0).

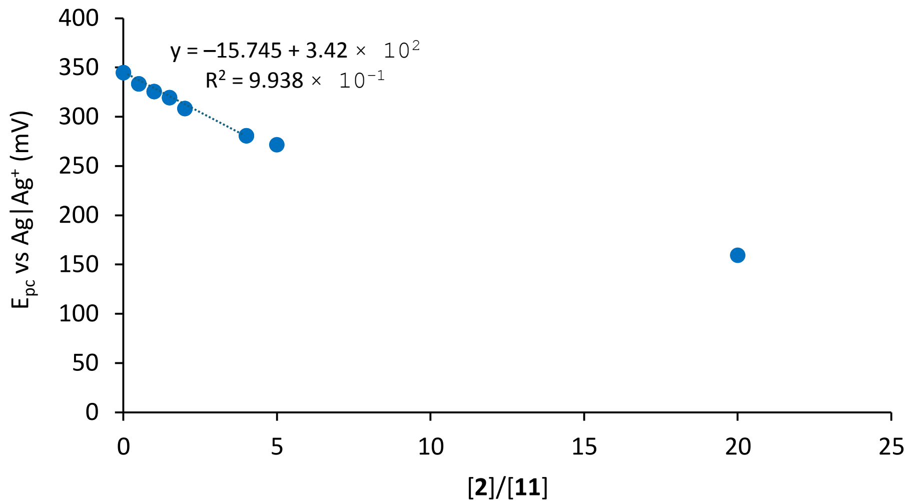

Figure S39. Slope of the cathodic peak potential of LSV response curves of **11** vs. [**2**]/[**11**] ratio at low ratios (0.56 mM **11** in CH_2Cl_2, glassy carbon working electrode (OD = 3 mm), Ag|Ag$^+$ reference electrode, Pt-wire counter electrode, 0.1 M [nBu$_4$N][PF$_6$] supporting electrolyte, 100 mVs^{-1} scan rate).

Table S3. Elemental composition of the surface of electrode **E9b**

eZAF Smart Quant Results

Element	Weight %	Atomic %	Net Int.	Error %	K ratio	Z	A	F
CK	36.01	48.77	3887.01	8.68	0.1451	1.0651	0.3782	1.0000
OK	43.65	44.38	6900.35	9.44	0.1036	1.0199	0.2326	1.0000
AlK	0.80	0.48	403.19	10.09	0.0042	0.9081	0.5837	1.0026
SiK	0.90	0.52	560.88	7.97	0.0060	0.9281	0.7112	1.0041
SK	0.26	0.13	151.92	19.27	0.0021	0.9092	0.8851	1.0105
ClK	0.65	0.30	358.59	9.65	0.0053	0.8650	0.9349	1.0157
KK	1.32	0.55	641.54	6.43	0.0116	0.8610	0.9890	1.0298
CaK	0.65	0.26	272.53	11.99	0.0059	0.8767	0.9973	1.0391
CrK	0.70	0.22	217.81	15.74	0.0063	0.7867	1.0174	1.1379
FeK	15.06	4.39	3329.50	2.90	0.1231	0.7820	1.0170	1.0276

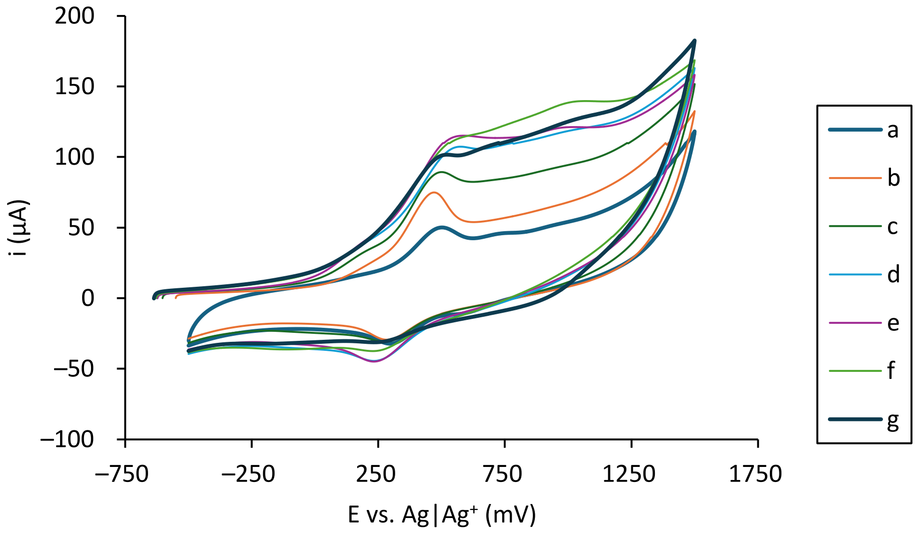

Figure S40. Cyclic voltammograms of **E9b** in solutions of **2** of different concentration, concentration of **2** in mM: a: 0, b: 0.1, c: 0.2, d: 0.4, e: 0. 5, f:0.75, g: 1.0) (Ag|Ag^+ reference electrode, Pt-wire counter electrode, 0.1 M [$n$$Bu_4N$][$PF_6$] supporting electrolyte in CH_2Cl_2, 100 mVs^{-1} scan rate).

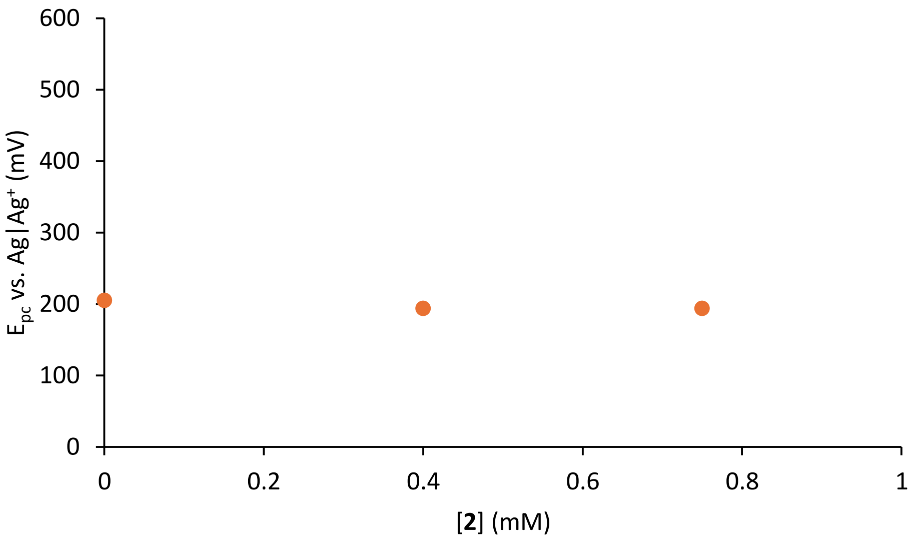

Figure S41. Change of the cathodic potential of **E9a** as a function of **2** concentration (Ag|Ag$^+$ reference electrode, Pt-wire counter electrode, 0.1 M [nBu$_4$N][PF$_6$] supporting electrolyte in CH_2Cl_2, 100 mVs^{-1} scan rate).

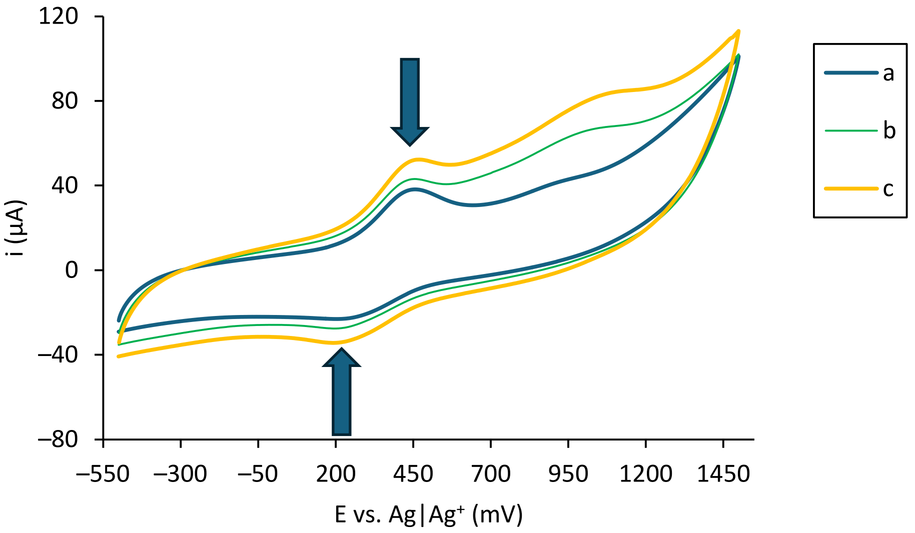

Figure S42. Cyclic voltammograms of **E9a** in solutions of **2** of different concentration with indication of Fe^{2+}/Fe^{3+} redox waves (Ag|Ag$^+$ reference electrode, Pt-wire counter electrode, 0.1 M [nBu$_4$N][PF$_6$] supporting electrolyte in CH_2Cl_2, 100 mVs^{-1} scan rate, concentration of **2**: a: 0 mM, b: 0.4 mM, c: 0.75 mM).

S3. Supplementary computational data

*S3.1. Cartesian coordinates and the internal energy of conformer **10'***

E -2619.536923153558

C	0.40224282451247	-0.98645718389989	-0.67520649828213
N	-0.91106220487304	-1.11928080938387	-0.93022491680494
C	-1.61953720880301	0.01527415583238	-0.99420423187817
C	-1.01314697945880	1.27006969808052	-0.81509056311978
C	0.34529264683863	1.28734602936837	-0.52939686843687
N	1.05795618109274	0.15787935418947	-0.45512748099885
H	-1.58557252324883	2.18214033915521	-0.92320869317005
N	1.10881826433747	-2.16471791174504	-0.63006977890549
H	0.56391533415842	-3.00474040465703	-0.75843074145246
C	2.49401587517269	-2.43081614060700	-0.45634301545114
O	2.84904576887553	-3.60347746373227	-0.40761649969387
N	3.31097427901835	-1.35893660394507	-0.38871098236783
H	2.87784248204645	-0.43554707273027	-0.36137089138541
C	-3.04656827942460	-0.11493646551322	-1.26355645353698
C	-4.03158966545951	0.93539846763792	-1.26605322480881
C	-3.74154426191483	-1.36016606820608	-1.43902143127893
C	-5.31082075275254	0.33684828187341	-1.45519557281096
H	-3.84849975413540	1.99231073086260	-1.13048337328768
C	-5.13054288504990	-1.07604623268258	-1.56090631877249
H	-3.27150750126228	-2.33246530753499	-1.45024811849809
H	-6.25661791979982	0.86125292676618	-1.47919469606201
H	-5.91856614071126	-1.80801027254714	-1.67607126720755
Fe	-4.39816598441768	-0.39199189023535	0.23268420123048
C	-5.60888200952268	0.03789597410369	1.82851488782711
C	-4.32496155523727	0.64102110835454	2.00177353187866
C	-5.42030265749604	-1.37318418682851	1.71072228490297

H	-6.55398464352740	0.56022942966688	1.76625141700844
C	-3.34590428654380	-0.39788036730984	1.99201364280935
H	-4.12731629992768	1.70025384925741	2.09675322788479
C	-4.02047516098988	-1.64220487777960	1.81118840316128
H	-6.19773129992812	-2.10575494092233	1.54092673196906
H	-2.27377886916419	-0.26277094063025	2.05586071036372
H	-3.54928533594745	-2.61196972553784	1.72753620205507
C	4.73628154054998	-1.50549917850083	-0.12804476766929
C	5.48592514845189	-0.33388205826831	-0.76533423580637
C	5.02474247194029	-1.58859239049916	1.37690197889143
H	5.06178787867704	-2.44457667898121	-0.59643052964926
C	6.98784560353943	-0.42262405036001	-0.48616877514747
H	5.08662465036302	0.60646666323472	-0.35419082123212
H	5.28980213202056	-0.31697993190456	-1.84438301249888
C	6.52753706232324	-1.68200924589486	1.64782810478292
H	4.61667849953337	-0.68775445665645	1.85939509696075
H	4.49522135744265	-2.45314273490990	1.79413506761013
C	7.27221353484055	-0.50423586860652	1.01533371913323
H	7.50177074415200	0.43967230186645	-0.92841525280238
H	7.39360355008590	-1.31798631123728	-0.97932258936915
H	6.71340645611509	-1.71912141692531	2.72811171596476
H	6.91443184228814	-2.62279685385673	1.22970370906458
H	8.35106000946935	-0.59174819307777	1.19399306727812
H	6.94327846385551	0.42943339059776	1.49530836720350
C	1.09830951189961	2.54756219947010	-0.28870393404560
C	0.45030852172505	3.70912347582260	0.14310631534761
C	1.19988740520656	4.85676856928435	0.36015047743105
H	-0.61797651552049	3.70811273319339	0.33331921150514
C	3.13003931907306	3.60997634237510	-0.28002881949050

C	2.57056714813880	4.81388793222219	0.14013372408385
H	0.72047239639763	5.76867954363451	0.70419103428441
H	4.20265573789410	3.53574452796610	-0.45940723418097
H	3.19622319453390	5.68713361515133	0.29628606870787
N	2.42470285854704	2.49908259613991	-0.48791130923779

*S3.2. Cartesian coordinates and the internal energy of conformer **10**”*

E -2619.534743411177

C	0.50820837057723	-0.70384739949030	-0.80737490347357
N	-0.78761105422025	-0.83992128340007	-1.11339454816442
C	-1.53474544134928	0.27790426047326	-1.10032558680795
C	-0.96967109380017	1.51470511899222	-0.78880841922692
C	0.39432171213078	1.54093659935549	-0.49058378733574
N	1.14429932120041	0.43857257014693	-0.49350755999856
H	-1.55547763775458	2.42429571509155	-0.83856763913977
N	1.31869101008619	-1.81780268605626	-0.79909403982273
H	2.29446950670915	-1.62757242286804	-0.61914118023426
C	1.06497629176047	-3.18365189764154	-1.06338998656889
O	2.01603328931637	-3.95462024530683	-1.09950664600257
N	-0.23423502496537	-3.53980555675519	-1.21952754698535
H	-0.90641504794101	-2.77853803810770	-1.29407292168794
C	-2.94303495330518	0.12952426437577	-1.44515574629113
C	-4.00406733203599	1.06141832515011	-1.17186504718599
C	-3.53778111618175	-1.04396921317568	-2.02580099271340
C	-5.23048636807296	0.46821436755069	-1.58987668727137
H	-3.90050406031506	2.02715676317179	-0.69628598870596
C	-4.94193944531981	-0.82656986614333	-2.11877239963519
H	-3.00131283888788	-1.92962277111221	-2.33506667041680
H	-6.21312147569186	0.90885157391089	-1.48867384258211

H	-5.66813268841701	-1.53481332038770	-2.49416905292017
Fe	-4.30160449989486	-0.69269612784175	-0.17356405185814
C	-5.58506625948470	-0.86562028817518	1.41359194850659
C	-4.36631274155237	-0.24352753838203	1.82552859361143
C	-5.26554873939516	-2.15470865331050	0.88714325057978
H	-6.57203872394412	-0.42573160992510	1.46224058722632
C	-3.29640178762888	-1.14843368141384	1.55424758707108
H	-4.26799216241218	0.75056449823075	2.24028481549641
C	-3.84965237916696	-2.32880705048398	0.97448284417705
H	-5.96818937953300	-2.86151596450685	0.46691428660888
H	-2.24265310179540	-0.95516867223428	1.70821269411814
H	-3.28854578332368	-3.18733356715295	0.63169982964719
C	-0.58674278483557	-4.87669716270046	-1.68091186171310
C	-1.97635535618094	-5.25379184261138	-1.16532082536630
C	-0.52348599856353	-4.97789476698106	-3.21041760045603
H	0.15368875922886	-5.56716783394281	-1.25560278798271
C	-2.38863335810786	-6.64512068100639	-1.65285472641294
H	-2.70790014182444	-4.51171094206256	-1.52555283096566
H	-1.98286658092903	-5.21088891553770	-0.06878373923887
C	-0.93144740498627	-6.37180509876903	-3.69065341764206
H	-1.20220539572232	-4.22464757095335	-3.63997186652536
H	0.49143764587171	-4.73006739385287	-3.54242868285932
C	-2.32432834846725	-6.74409645828843	-3.17826611553176
H	-3.39713635582778	-6.88252161189939	-1.29306232139433
H	-1.71289213446233	-7.39305388762779	-1.21357256112156
H	-0.89999647005900	-6.41654143379132	-4.78588331936084
H	-0.20101839738699	-7.10777506676341	-3.32455224154369
H	-2.59444414857512	-7.75602093117627	-3.50390081878478
H	-3.06623323967531	-6.06049166874969	-3.61735295931426

C	1.07750935163657	2.82142628876638	-0.16333762532971
C	0.40926561742522	3.81966587323613	0.55233227957079
C	1.07950132161063	5.00103067553527	0.83955464253460
H	-0.60606288862991	3.66037595153413	0.90243827334227
C	2.96882793162583	4.10064046107284	-0.30811921898694
C	2.38728732355350	5.15168894857066	0.39714074876227
H	0.58924438618701	5.78809616202874	1.40527707918088
H	3.99298035560109	4.18341144716596	-0.67157209233735
H	2.94996443135564	6.05865574324278	0.59565289594991
N	2.34276720674649	2.95692101198293	-0.58298929848706

*S3.3. Cartesian coordinates and the internal energy of transition state TS-**10’-10”***

E -2619.509684514826

C	0.56385817940590	-0.30371720100779	1.12195171509799
N	-0.46921242652544	-0.83995854638146	0.47544506259959
C	-1.12571198643511	-0.03873149627640	-0.38776595263018
C	-0.71019987052639	1.28191383687883	-0.59159327953728
C	0.38766343748963	1.73350902977360	0.13029369714457
N	1.03611526051659	0.94057109059423	0.99992974342712
H	-1.20073191328648	1.91869685665209	-1.31938449941085
N	1.24571550247563	-1.16347129256404	2.03220647116204
H	1.15908114342635	-0.91035719699715	3.00725138580124
C	2.53641864394565	-1.64805431585857	1.76795070545143
O	3.22952639607674	-2.10199353485896	2.66755343083507
N	2.90354228036368	-1.65160699917319	0.44594938304299
H	2.41200234163657	-1.02519706971885	-0.17641059648927
C	-2.25746260389240	-0.62150270834934	-1.09205781855432
C	-3.21525603468684	0.06154288617134	-1.92240242107636
C	-2.69393396796996	-1.98425332432700	-0.95462333406355

C	-4.21607712887750	-0.87975023830704	-2.29987393326073
H	-3.19966220521189	1.10660120434496	-2.20008002427541
C	-3.89328174995970	-2.13739564451089	-1.70444431070739
H	-2.19204975173050	-2.73390430997759	-0.36047130510346
H	-5.08738648427727	-0.66736060253045	-2.90465312321228
H	-4.48061011692066	-3.04256736827199	-1.78015419236395
Fe	-4.11198926928948	-0.69513802657567	-0.25799578742838
C	-5.96865070267582	-0.14423982847135	0.41231455760906
C	-5.00625687596377	0.86119863754327	0.73528556453140
C	-5.55491898764187	-1.36417793598770	1.02983321615616
H	-6.84078524665980	-0.01246005537664	-0.21374057947698
C	-4.00092676048799	0.26147191081590	1.55193371374338
H	-5.02252244437351	1.88853811081709	0.39716221390634
C	-4.33675765323757	-1.11371803414886	1.73357887064714
H	-6.05837096398855	-2.31822235838537	0.95076143834369
H	-3.10914701415232	0.74881501333328	1.92481567206479
H	-3.74888546074196	-1.84296814190299	2.27408433449740
C	4.29006854783267	-1.93377725572902	0.08009314241738
C	4.35531863435848	-2.36716357872114	-1.38462455948200
C	5.20547133737336	-0.73064144490817	0.33810181034072
H	4.61679253071591	-2.76589939760776	0.71730009876937
C	5.79706049174034	-2.66869914637039	-1.80278799241754
H	3.95750693769634	-1.55761089076190	-2.01749975772579
H	3.71231985942390	-3.24231573338031	-1.53821099491594
C	6.64575353431569	-1.03713197502587	-0.07795736888517
H	4.82687114496206	0.12899954759562	-0.23577203581311
H	5.15162164552996	-0.46317953005673	1.39934978123767
C	6.71505772881445	-1.47178010700772	-1.54358116580610
H	5.82728486406273	-2.95492207301973	-2.86117569562828

H	6.16199664668951	-3.53414301387690	-1.23088670025924
H	7.28194879655209	-0.16027469758103	0.09305331296442
H	7.04179888518629	-1.84204146343497	0.55828682107236
H	7.74707171883409	-1.71855439786249	-1.82219396505835
H	6.40433093078112	-0.63320272469973	-2.18426293700941
C	0.91045687894596	3.11362829476984	-0.04472256562872
C	0.03682362940421	4.19025268839330	-0.22272589084711
C	0.56834981694552	5.46296690140824	-0.38201404390242
H	-1.03798483915573	4.03537880870599	-0.20581810512056
C	2.73716287714119	4.48271350019315	-0.18876643439009
C	1.94858984951847	5.61680774365141	-0.36911670162880
H	-0.08581751311353	6.32097555585705	-0.50726515708674
H	3.82372457984463	4.56499518340680	-0.17593523969481
H	2.40888570969309	6.59240661375711	-0.49104566934686
N	2.24264988408321	3.25619646234003	-0.02400560562589

*S3.4. Cartesian coordinates and the internal energy of the adduct **10'·2***

E -2978.504343389724

C	-0.54305944705154	0.21457752990161	-0.45759545145599
N	0.79272478071659	0.24875177926594	-0.64906646106570
C	1.40636364244462	-0.92658304995095	-0.82409846553019
C	0.68869117014623	-2.13392970792421	-0.84293069189851
C	-0.68145135342876	-2.06049687907346	-0.63407582673768
N	-1.29701805216045	-0.89025156118109	-0.43402180025260
H	1.19502902487938	-3.07484410740692	-1.01431190050923
N	-1.13494107633095	1.43429490064556	-0.26430251022796
H	-0.50889089060986	2.25822767679720	-0.28898422696381
C	-2.48944987964491	1.75326823853685	-0.03440739682557
O	-2.77830156722869	2.92350496566729	0.23819802233117

N	-3.39682365812478	0.76445252855963	-0.15227843543785
H	-3.04196801013704	-0.18263334235319	-0.29388086223982
C	2.85722793601898	-0.89213903580858	-0.97459658211405
C	3.71969398372720	-1.98556877840822	-1.33938224629539
C	3.69456644373220	0.22652231145516	-0.63570466848537
C	5.06737109149826	-1.53358457361269	-1.23921424750803
H	3.40942777851691	-2.97682017418499	-1.63966266493049
C	5.05118538677088	-0.17335216135603	-0.80315690115312
H	3.34026950638141	1.18082150758621	-0.27209157804437
H	5.94896314575092	-2.13111662425972	-1.42813651983164
H	5.92039218015600	0.43592493085659	-0.59496764400493
Fe	4.09950460797466	-1.37403116245409	0.56459078167573
C	5.11379165342924	-2.52365219099236	1.92290196256964
C	3.75707825713603	-2.96034103952647	1.81627301929332
C	5.10477197352553	-1.15600809029674	2.33541404108955
H	5.99182731307082	-3.11517441287163	1.70128551924586
C	2.91320526926391	-1.86238728539704	2.16421347834489
H	3.42832941131051	-3.94203381791698	1.50263539210717
C	3.74380572616080	-0.74704295318043	2.48386934645587
H	5.97516457484833	-0.52956250627038	2.47645042732125
H	1.83102672039617	-1.85812008804035	2.13379347874289
H	3.39972898106296	0.24322696634135	2.74974172356599
C	-4.80471690926725	0.98081247084500	0.15352061636693
C	-5.64096014056816	-0.05655556720358	-0.59750910055351
C	-5.07142122219269	0.90981520050315	1.66296630145556
H	-5.07164949589388	1.98687706907455	-0.19931221110555
C	-7.13007149812595	0.10598495833258	-0.28532869465253
H	-5.30579091458995	-1.06219537646778	-0.29923275411584
H	-5.45630274325536	0.03302528745678	-1.67509209248524

C	-6.56055508075477	1.08139672617836	1.96822792906461
H	-4.72847575767548	-0.06877161678987	2.03089832589186
H	-4.47485580378926	1.67832902882158	2.16781784404183
C	-7.39640118867485	0.04030871770912	1.22039640296192
H	-7.70787230929986	-0.66381653295781	-0.81151458843396
H	-7.47536527358378	1.07652521656307	-0.67041498617832
H	-6.73349108930513	1.00907111239239	3.04887103347204
H	-6.88025881531280	2.08924838581334	1.66532645688098
H	-8.46430825485561	0.18748558376089	1.42394061022493
H	-7.13664226749435	-0.96305977574371	1.58940206772889
C	-1.55575154347572	-3.26468912394441	-0.61208183027239
C	-1.03744732600761	-4.53731117315600	-0.35147567923043
C	-1.89852003869419	-5.62556311596369	-0.34051259157866
H	0.01716240408209	-4.67306903686245	-0.13547599347648
C	-3.67595283947840	-4.11182349488807	-0.82827125232478
C	-3.24865524259577	-5.41544125194651	-0.58940979841428
H	-1.52036931003489	-6.62235789949980	-0.13270733515077
H	-4.72843393413882	-3.90565861435839	-1.02323146207773
H	-3.95828252959169	-6.23690333334286	-0.59178700827986
N	-2.86243541231675	-3.05704976263059	-0.83736939529044
C	1.68296425901708	3.92980464264519	-1.28576711431135
C	2.51687562103293	5.05423336379524	-1.38299296344802
C	2.21679196921950	6.14993190362343	-0.58661173727047
C	1.12709728656517	6.12378125500229	0.26746121793120
C	0.34170270753345	4.95754744170146	0.29877627839398
N	0.62631527864572	3.88060815903465	-0.45730759460926
H	2.84057458809719	7.03876878454130	-0.63666507891792
H	3.35971751314424	5.06155152886788	-2.06640145960042
H	0.87571158627580	6.97446572787431	0.89248536826124

N	-0.73495121395344	4.84518384236015	1.14089208686958
H	-1.45152819423191	4.17230746070770	0.86456118257647
H	-1.09649057595877	5.71369902116929	1.49993563737225
N	1.88280020098420	2.82328619573708	-2.08425274188430
H	1.51863502130846	1.95204982000102	-1.70616737173488
H	2.80345186500973	2.73889197809685	-2.48529963532794

S3.5. Cartesian coordinates and the internal energy of the adduct ***10”·2***

E -2978.503942761051

C	-0.92063015651308	-0.04537577232787	-0.59151057422526
N	0.34819377311219	-0.43025281932596	-0.79636353531348
C	0.61277571369542	-1.74534448040426	-0.75671183280001
C	-0.40288233805017	-2.67414837924777	-0.53035195202845
C	-1.69810739763284	-2.17783874773044	-0.37981757678822
N	-1.97159644689499	-0.87268548228088	-0.41224664614170
H	-0.20380899457942	-3.73830842080157	-0.55432056579643
N	-1.22294906577001	1.29313976202210	-0.54400582838335
H	-2.21242969250583	1.53639479817299	-0.35056745540968
C	-0.36997730133012	2.41215723140011	-0.57056604014984
O	-0.85541207282966	3.53401275299877	-0.40095628227468
N	0.94574898401423	2.20164838350258	-0.79347929240798
H	1.25131510719935	1.23179719309460	-0.85620118771660
C	1.99619862676514	-2.15033421577313	-0.97733584182120
C	2.59574495033485	-3.39903581159959	-0.59329557481197
C	3.03312741894294	-1.32797002680767	-1.53885801337273
C	3.98005297809103	-3.34476495454881	-0.92418770656839
H	2.09750239256940	-4.21969971703533	-0.09518110891730
C	4.24752716368093	-2.07103692915302	-1.51125870759628
H	2.89859196784669	-0.32575373193918	-1.92050219936533

H	4.70868095849507	-4.11946995215383	-0.72679798024130
H	5.21348490956723	-1.71613597090277	-1.84430885078281
Fe	3.48341406185263	-1.83730931530523	0.38130086902037
C	4.99201817924327	-1.23973317288505	1.64128217000777
C	4.23281565648901	-2.29258388035158	2.23501034738419
C	4.10553430436489	-0.15192327519148	1.37764147321326
H	6.04623181970569	-1.27260682976165	1.40145397712763
C	2.87661495854321	-1.85582154355198	2.33585021520065
H	4.61223645934874	-3.26324978820287	2.52488313599484
C	2.79856668786181	-0.53212002645170	1.80418591078513
H	4.37310145855131	0.78255960323509	0.90302374733248
H	2.04705396375180	-2.43534475960825	2.71829723818521
H	1.89675007172167	0.05587067383887	1.69056484065796
C	1.90695735786541	3.29440987153968	-0.72748134340291
C	2.26695866689033	3.64885445217265	0.72123290701095
C	3.15587967278617	2.91718064742504	-1.52393438937498
H	1.43817849302376	4.17456180770561	-1.18991654024688
C	3.30846171330163	4.76801009683404	0.77156391245411
H	2.66656815560265	2.74892607212329	1.21383692157339
H	1.35425900374950	3.93571754723611	1.25541431856972
C	4.20419210113203	4.03103567182274	-1.46738437351257
H	3.58468573956863	1.99428680151032	-1.10032053798549
H	2.87938429808450	2.69668937846814	-2.56289522849333
C	4.55997315162608	4.39040558963635	-0.02308638195956
H	3.56797485363641	4.99381122260758	1.81272815397818
H	2.87356010300019	5.68570331028154	0.34948865735783
H	5.10112941308719	3.72768639761792	-2.02085166322368
H	3.80758101121825	4.92261228175327	-1.97408030989321
H	5.28836883742228	5.21033216719286	-0.00532014164311

H	5.04563794210802	3.52659081221905	0.45614403463849
C	-2.84154288127290	-3.10870037038839	-0.18091098524432
C	-2.67933883841997	-4.27956085675192	0.56613008382970
C	-3.76653923943457	-5.12617180869757	0.73164179329301
H	-1.72570752933513	-4.50476940866700	1.03326283788967
C	-5.04212965464502	-3.59743700719180	-0.58273556081674
C	-4.97622529192831	-4.78477424629171	0.14080865405187
H	-3.67129796968002	-6.03498605931104	1.31874649620022
H	-5.97534899483641	-3.29954888039785	-1.06090709347912
H	-5.85293828252405	-5.41699575759210	0.24066962754701
N	-4.00991462493742	-2.76864163059509	-0.74210940892213
C	-4.33041820077316	3.23396533061821	0.78890541817465
C	-5.63404798424602	3.68450607890358	1.05832226132758
C	-6.69324565054261	2.93793879121282	0.56775767872787
C	-6.46105830502354	1.78217453156622	-0.16045037437540
C	-5.12642219599905	1.39787391732798	-0.37611301675722
N	-4.08445782731442	2.11072568323018	0.08931219507193
H	-7.71391322114730	3.26199682690403	0.75497366583804
H	-5.79544484561106	4.59292405420137	1.62957589698995
H	-7.27682178907124	1.18527401199861	-0.55528611625850
N	-4.82305029583828	0.28516220096575	-1.12170984520469
H	-3.93272884712420	-0.16279392892628	-0.92277906559013
H	-5.57178461168780	-0.37709983852428	-1.23971857479170
N	-3.23407143295185	3.90699796583321	1.27406615240212
H	-2.37578916246271	3.77963053048616	0.73883388180784
H	-3.40748793693844	4.86127434701743	1.54621023044503

*S4. ¹H- and ¹³C NMR spectra of chalcones **5a-5c**, 2-aminopyrimidines **6a-6c** and hosts **9a-c**, **10** and **11***

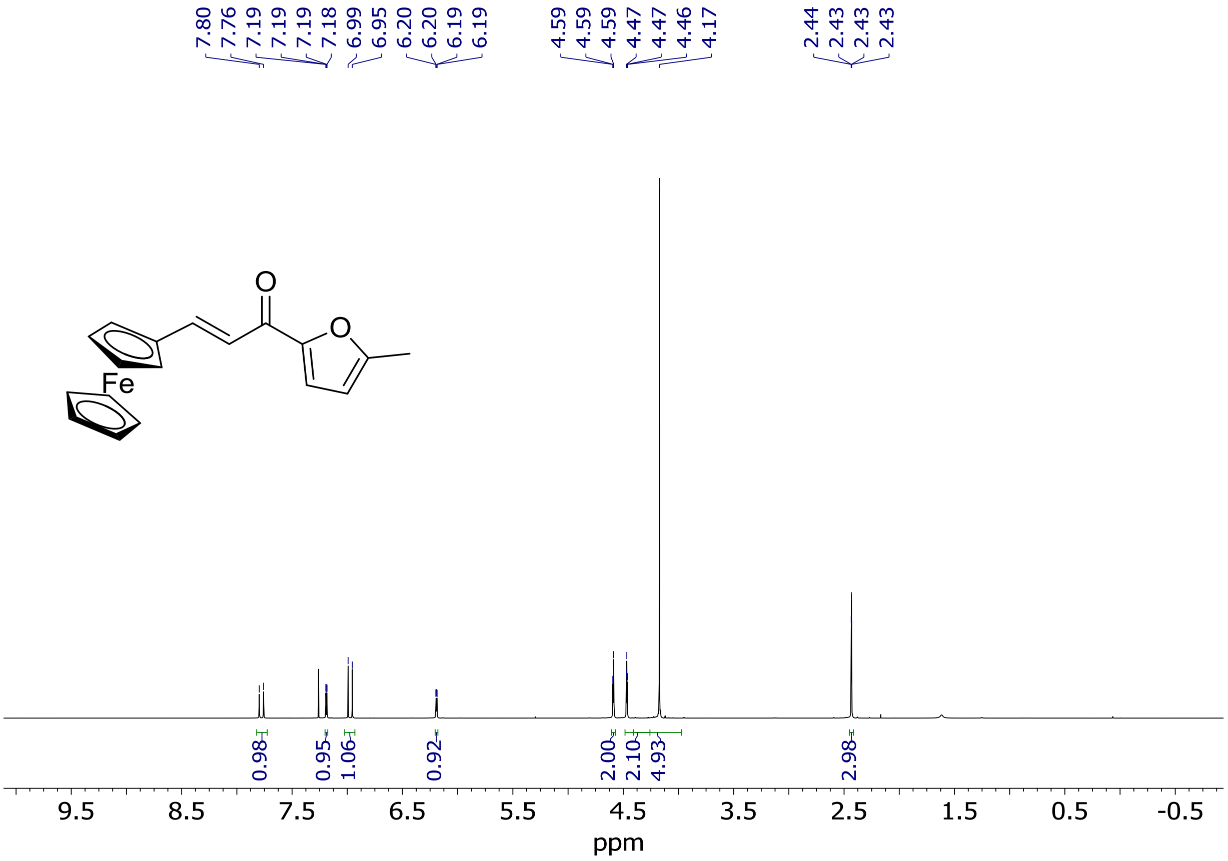

^{1}H NMR spectrum of compound **5a** ($CDCl_3$, 400.13 MHz).

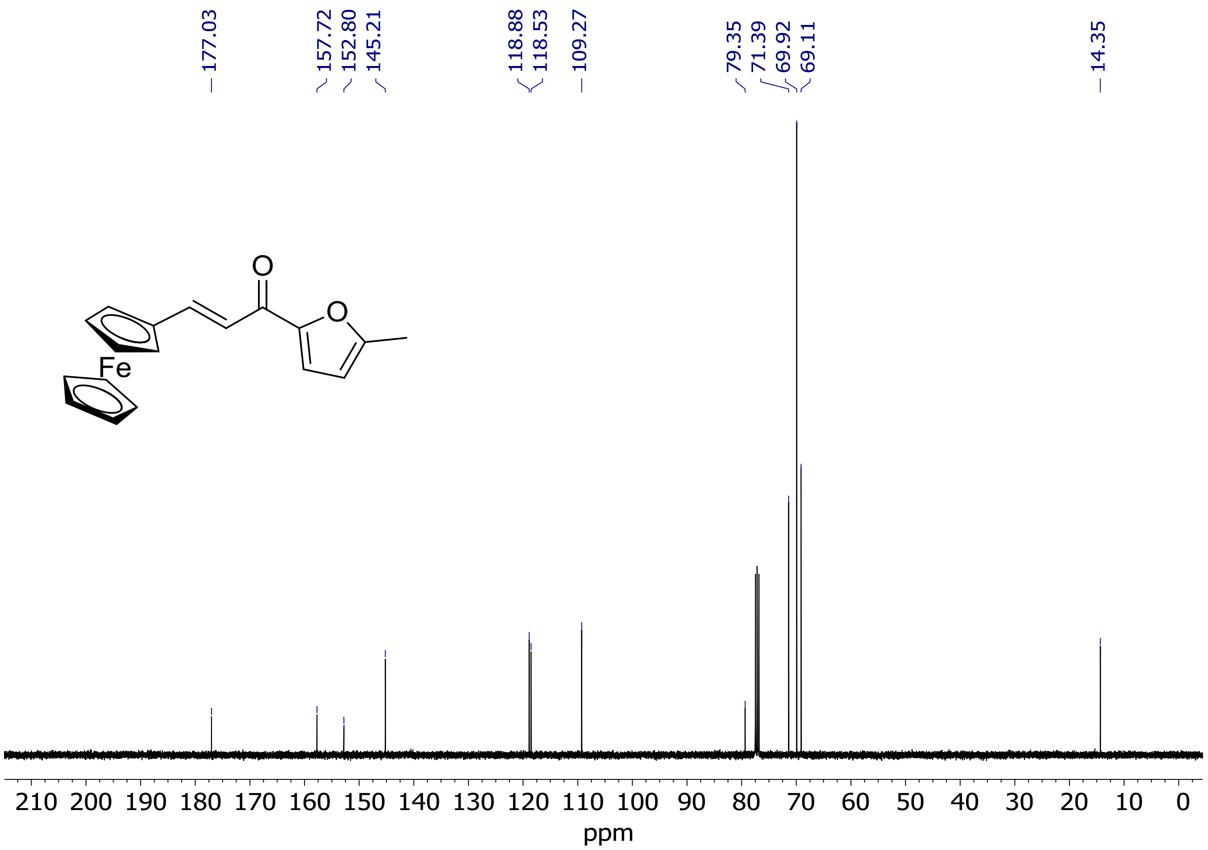

$^{13}C\{^{1}H\}$ NMR spectrum of compound **5a** ($CDCl_3$, 100.62 MHz).

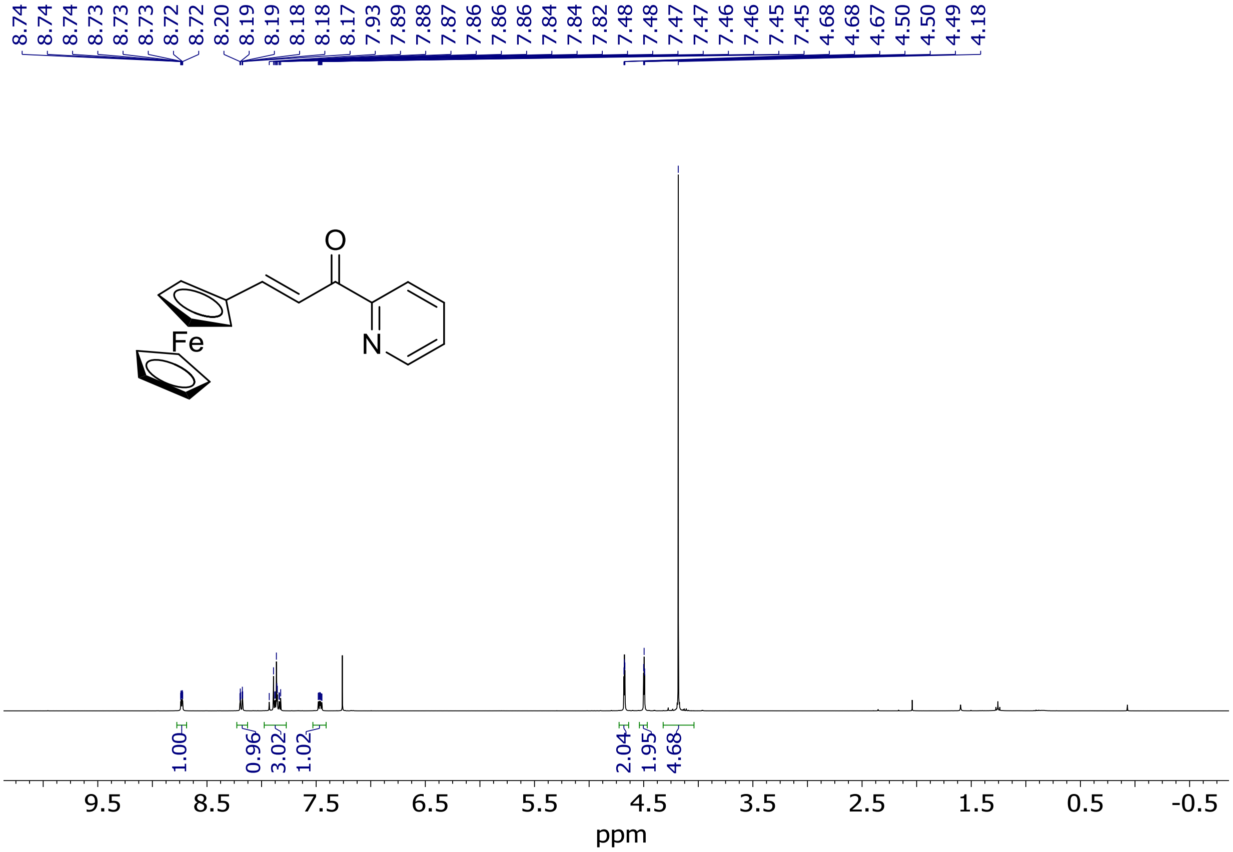

^{1}H NMR spectrum of compound **5b** ($CDCl_3$, 400.13 MHz).

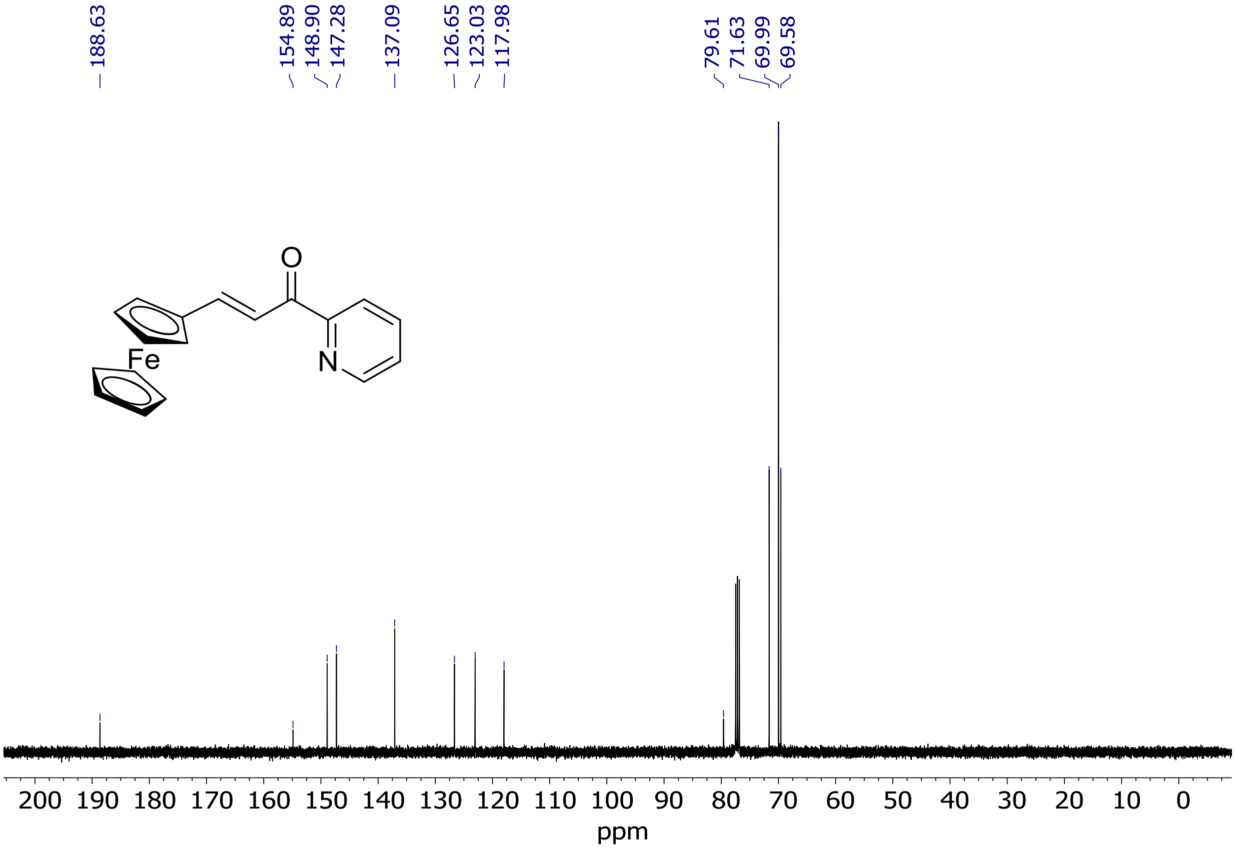

$^{13}C\{^{1}H\}$ NMR spectrum of compound **5b** ($CDCl_3$, 100.62 MHz).

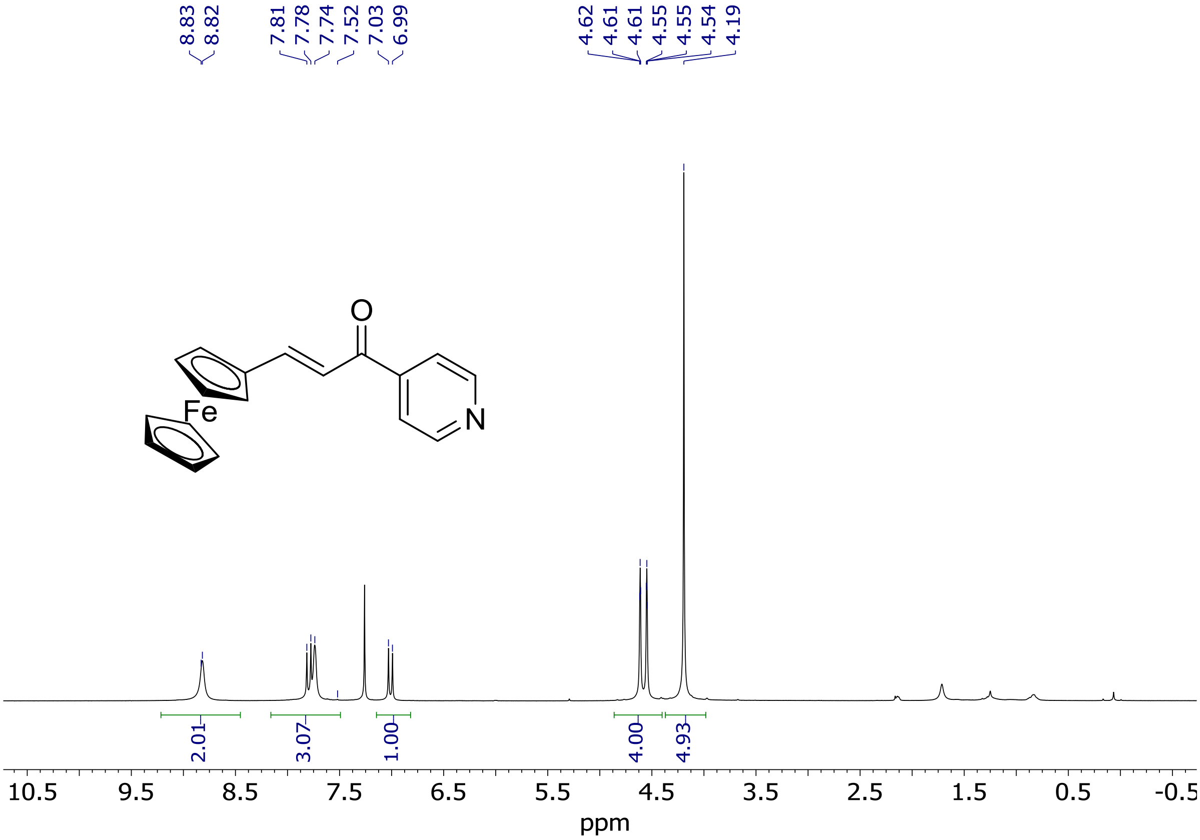

^{1}H NMR spectrum of compound **5c** ($CDCl_3$, 400.13 MHz).

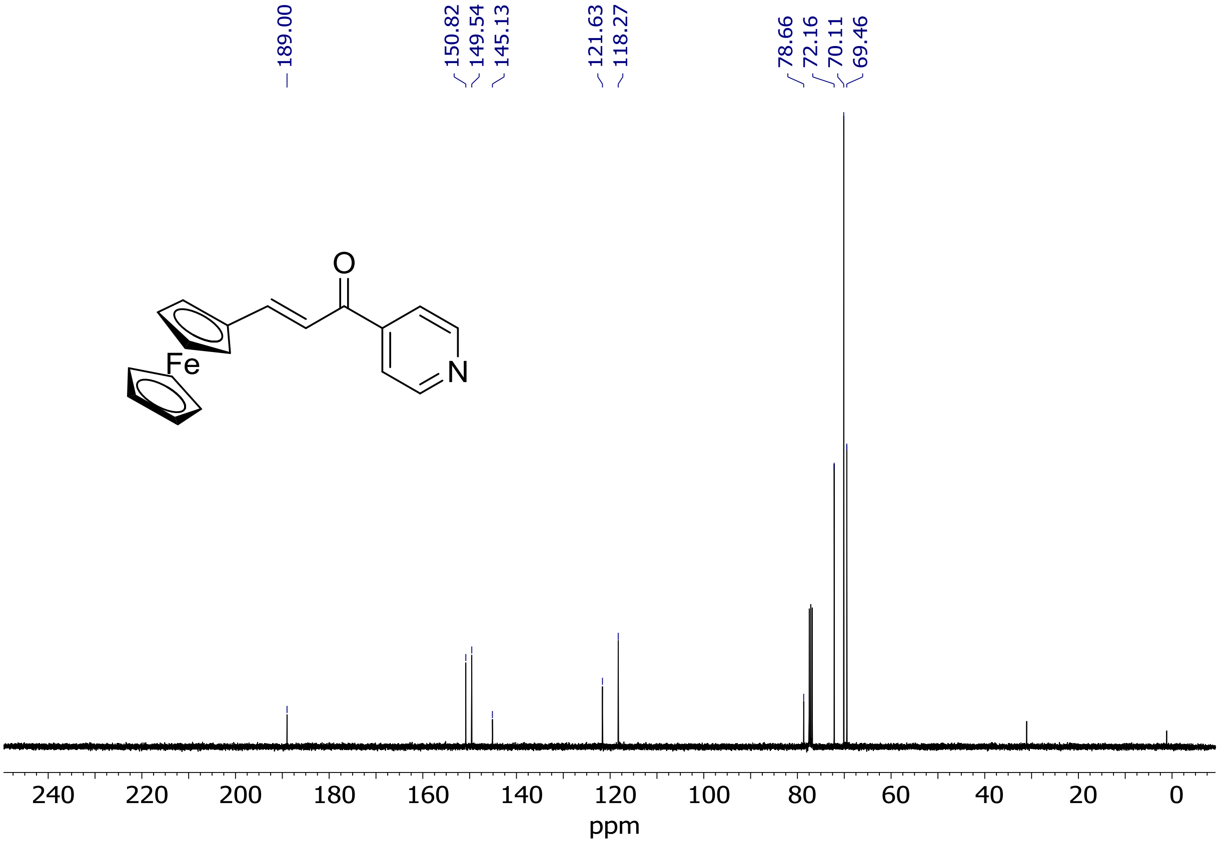

$^{13}C\{^1H\}$ NMR spectrum of compound **5c** ($CDCl_3$, 100.62 MHz).

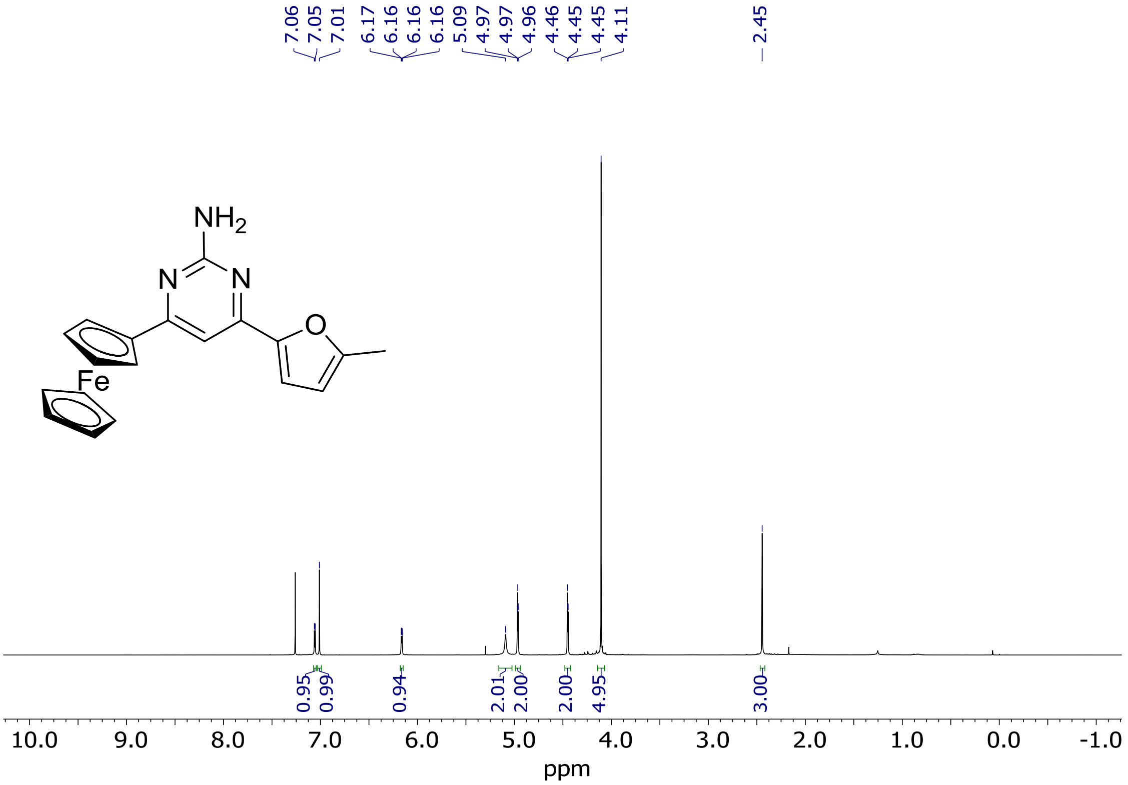

^1H NMR spectrum of compound **6a** ($CDCl_3$, 400.13 MHz).

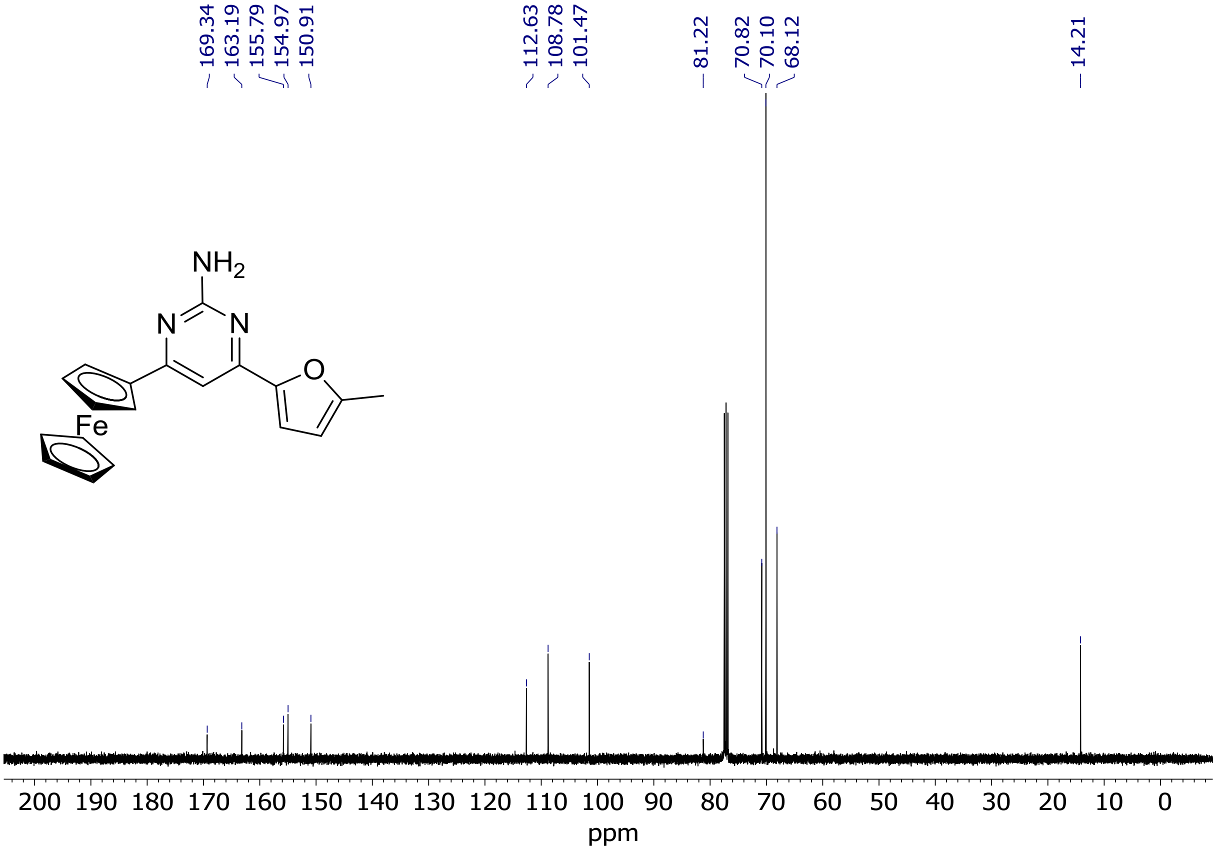

$^{13}C\{^1H\}$ NMR spectrum of compound **6a** ($CDCl_3$, 100.62 MHz).

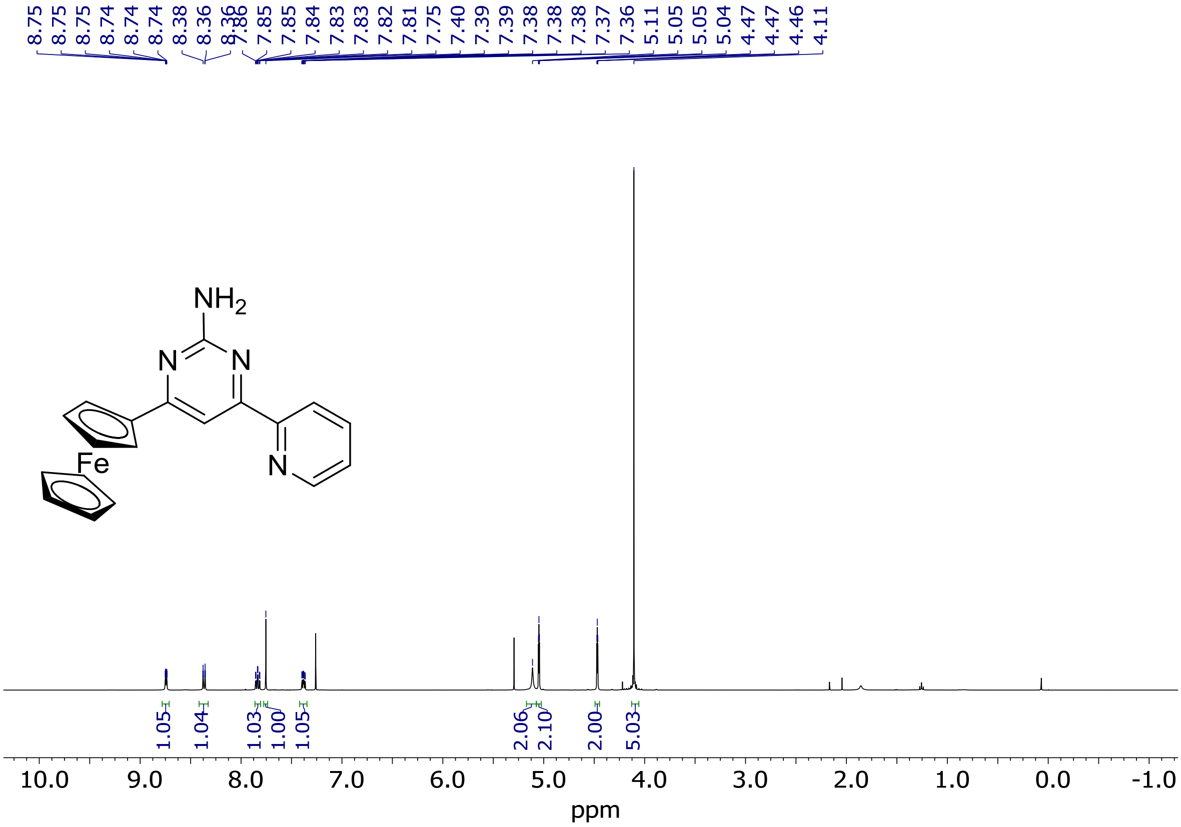

^1H NMR spectrum of compound **6b** ($CDCl_3$, 400.13 MHz).

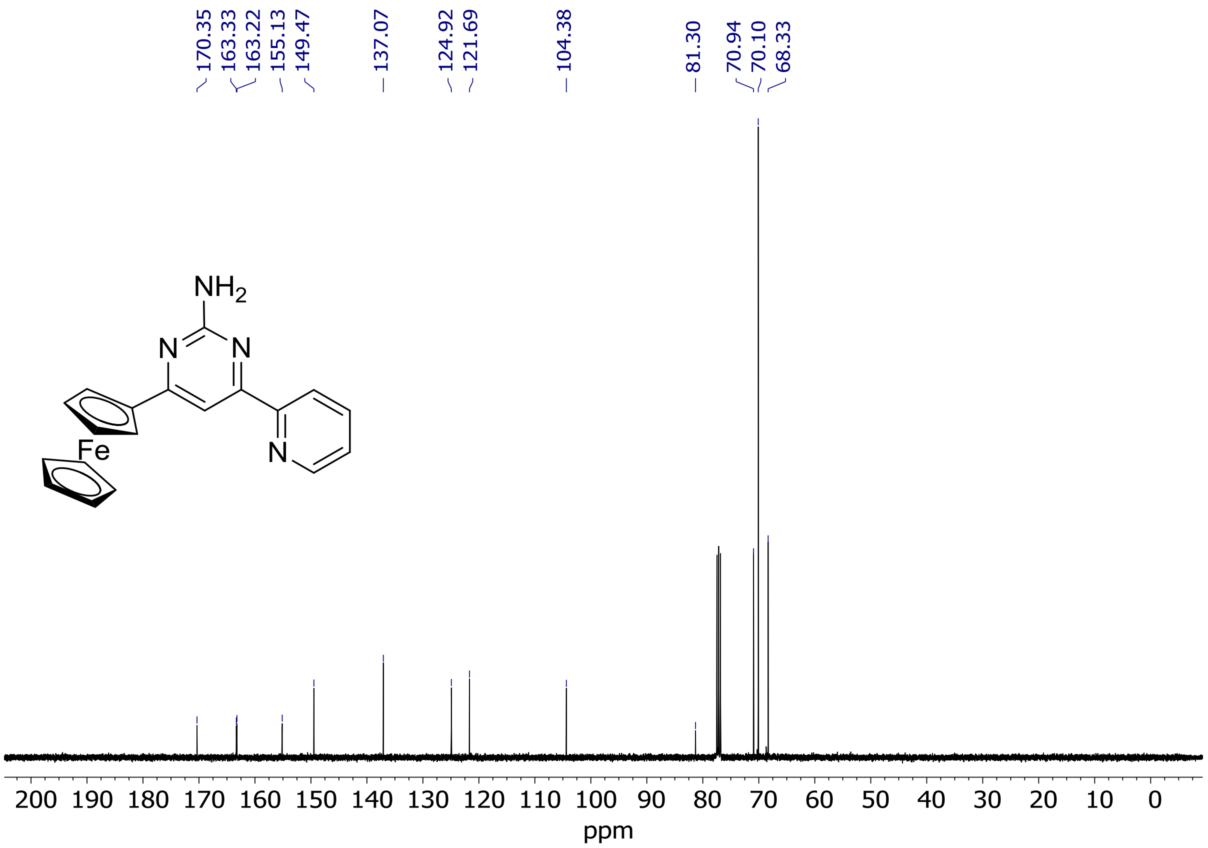

$^{13}C\{^1H\}$ NMR spectrum of compound **6b** ($CDCl_3$, 100.62 MHz).

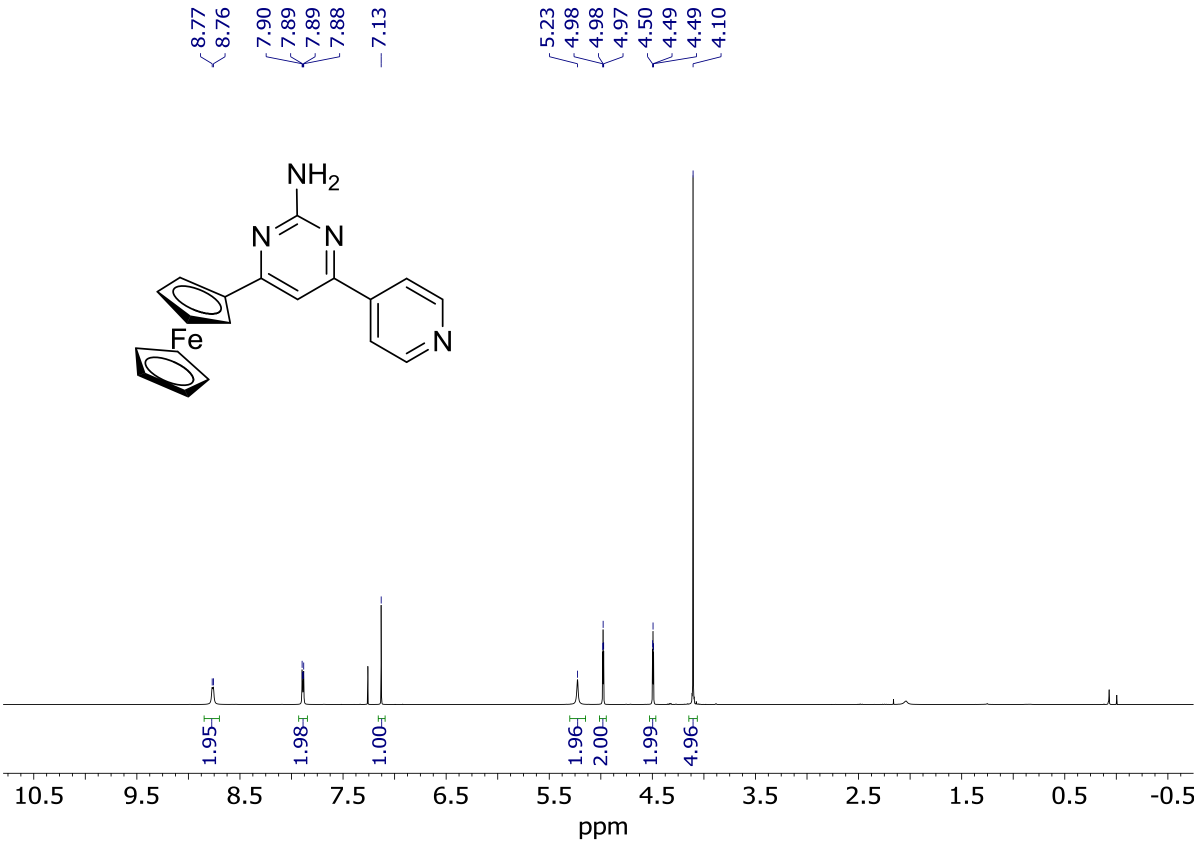

^1H NMR spectrum of compound **6c** ($CDCl_3$, 400.13 MHz).

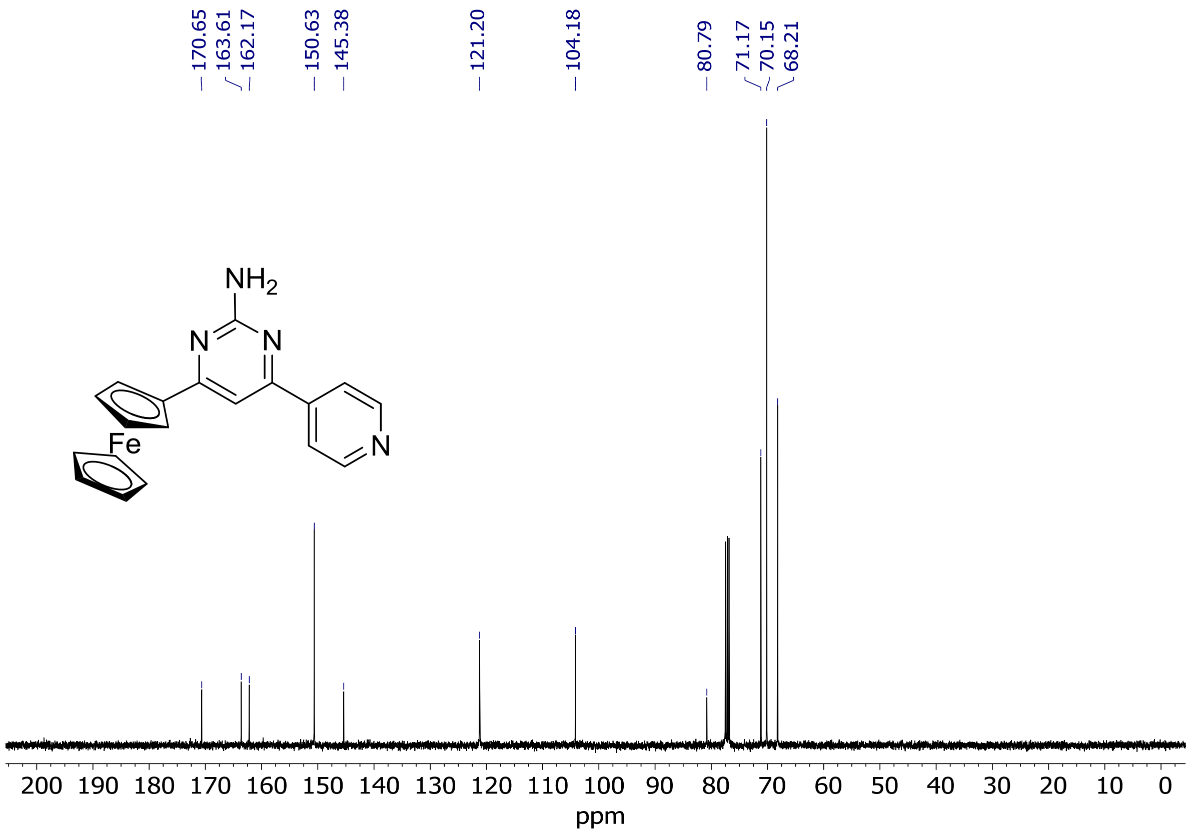

$^{13}C\{^1H\}$ NMR spectrum of compound **6c** ($CDCl_3$, 100.62 MHz).

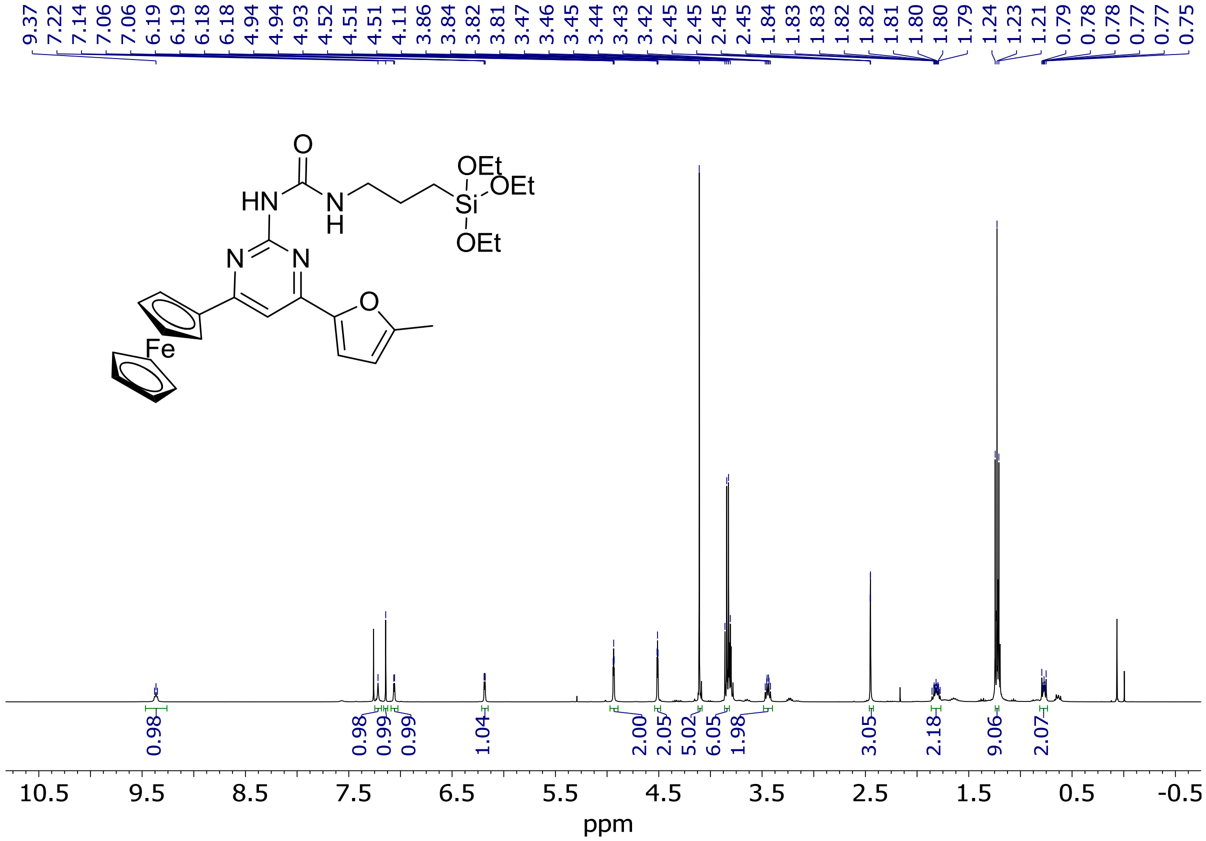

^1H NMR spectrum of compound **9a** ($CDCl_3$, 400.13 MHz).

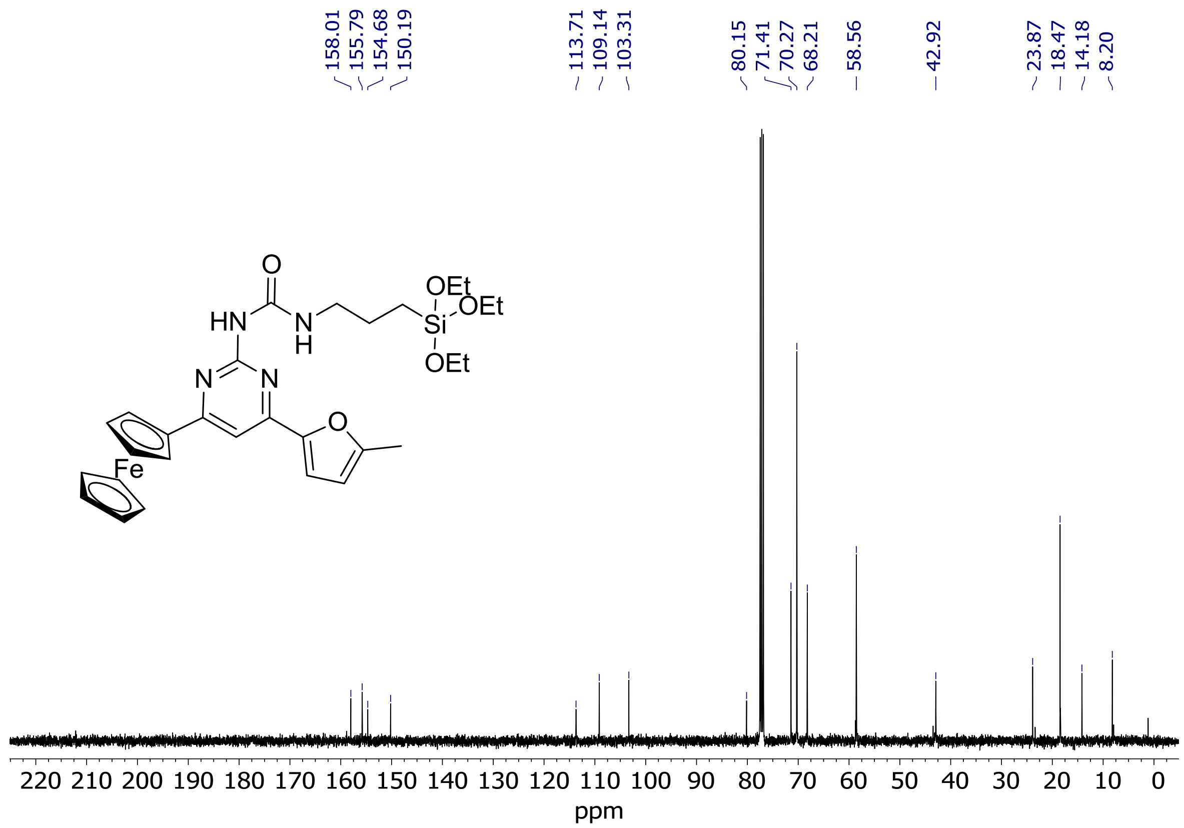

$^{13}C\{^{1}H\}$ NMR spectrum of compound **9a** ($CDCl_3$,100.62 MHz).

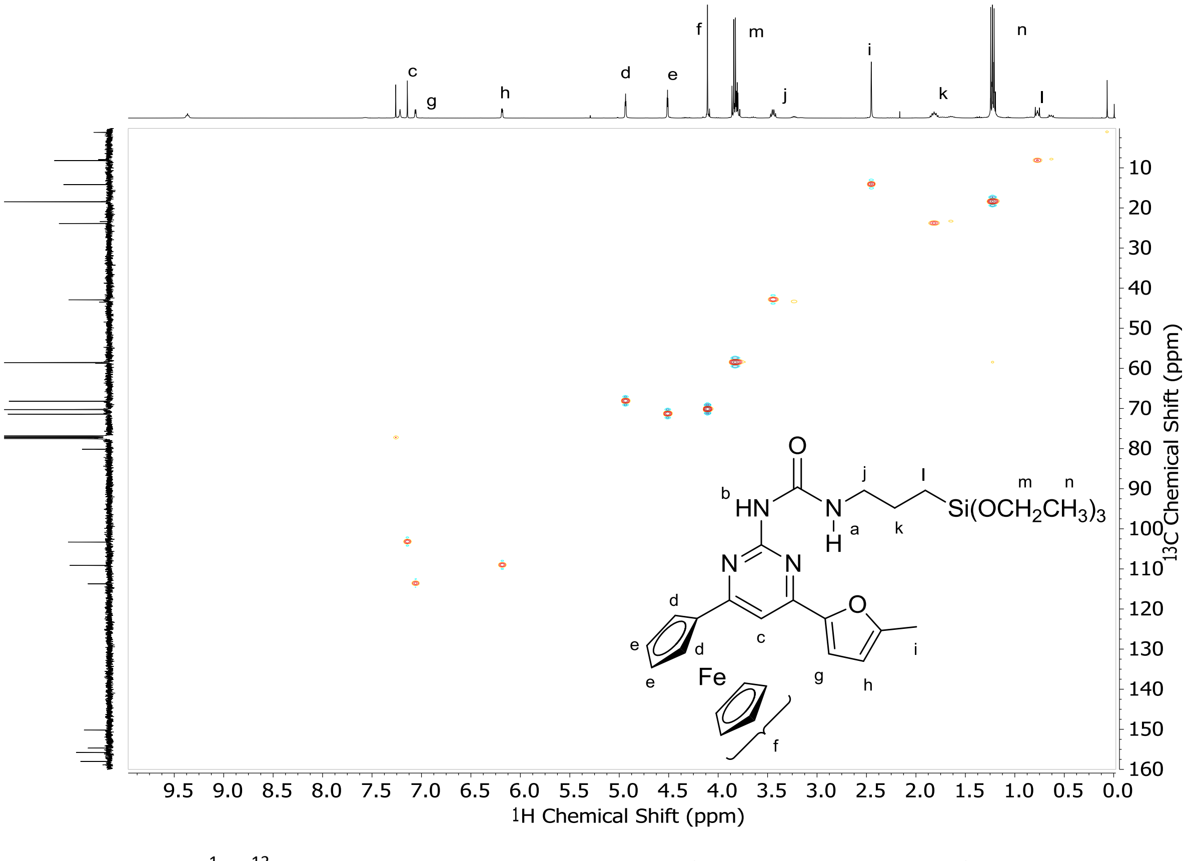

^{1}H–^{13}C HSQC spectrum of compound **9a** ($CDCl_3$, 298 K, 400 MHz).

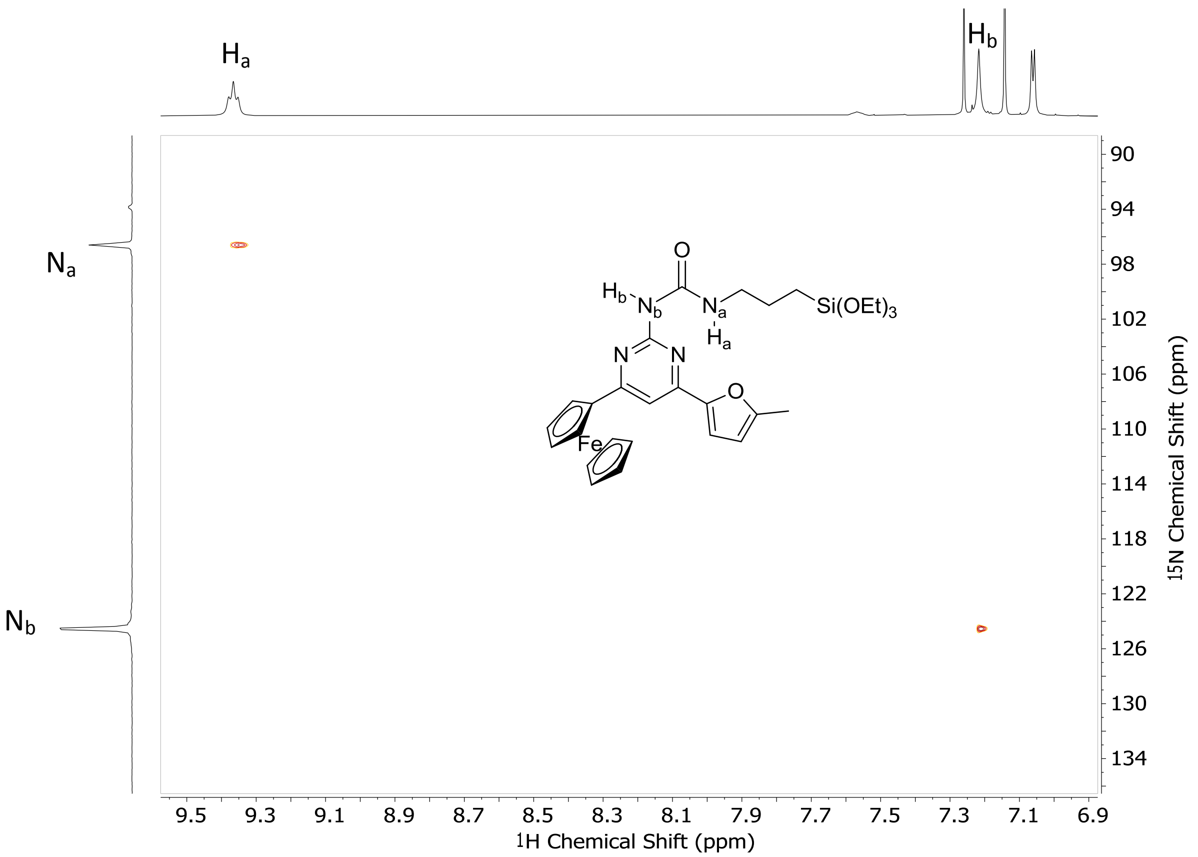

^{1}H–^{15}N HSQC spectrum of compound **9a** ($CDCl_3$), 298 K, 400 MHz).

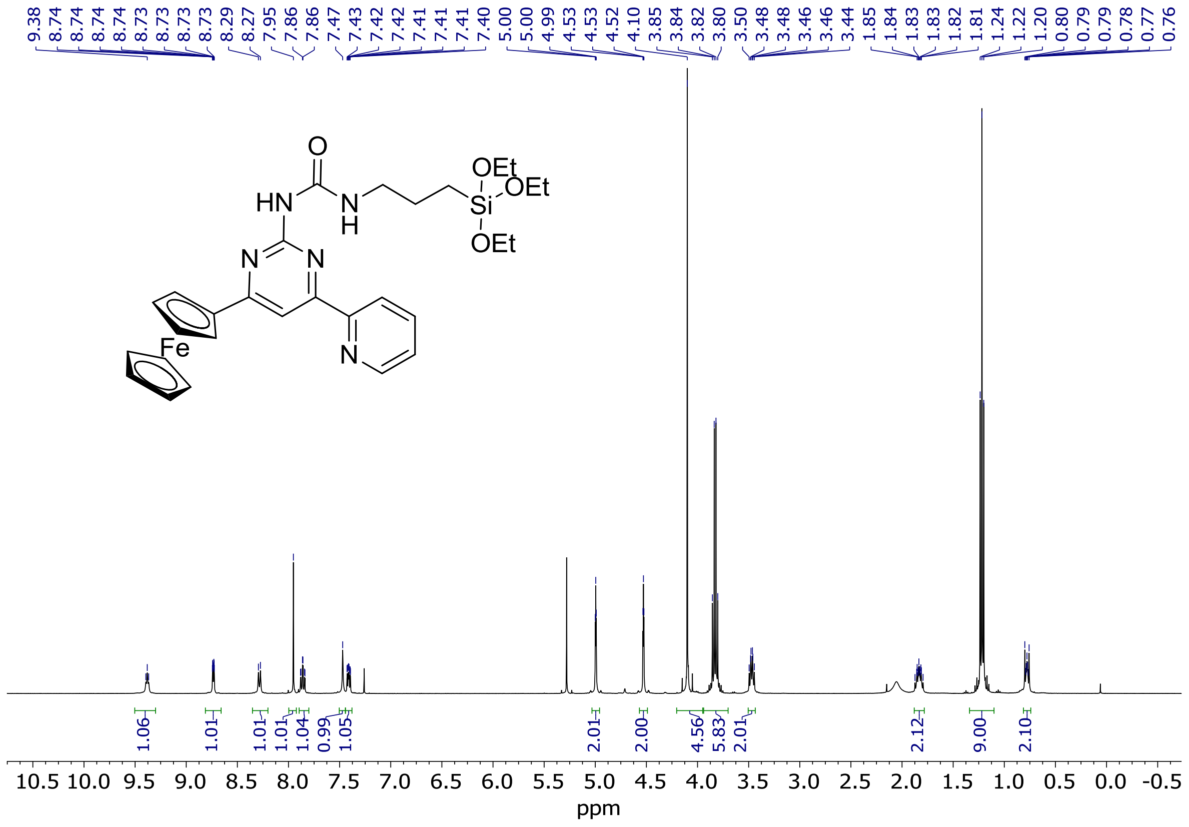

^{1}H NMR spectrum of compound **9b** ($CDCl_3$, 400.13 MHz).

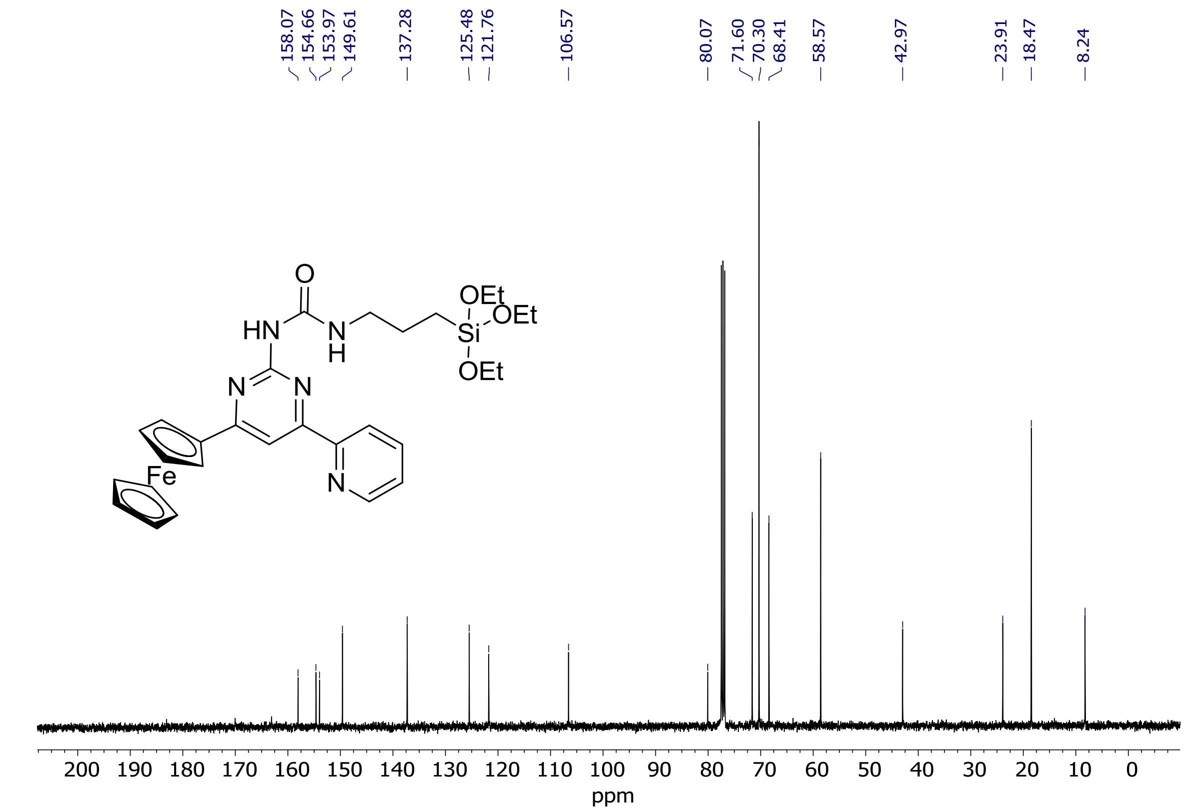

$^{13}C\{^{1}H\}$ NMR spectrum of compound **9b** ($CDCl_3$, 100.62 MHz).

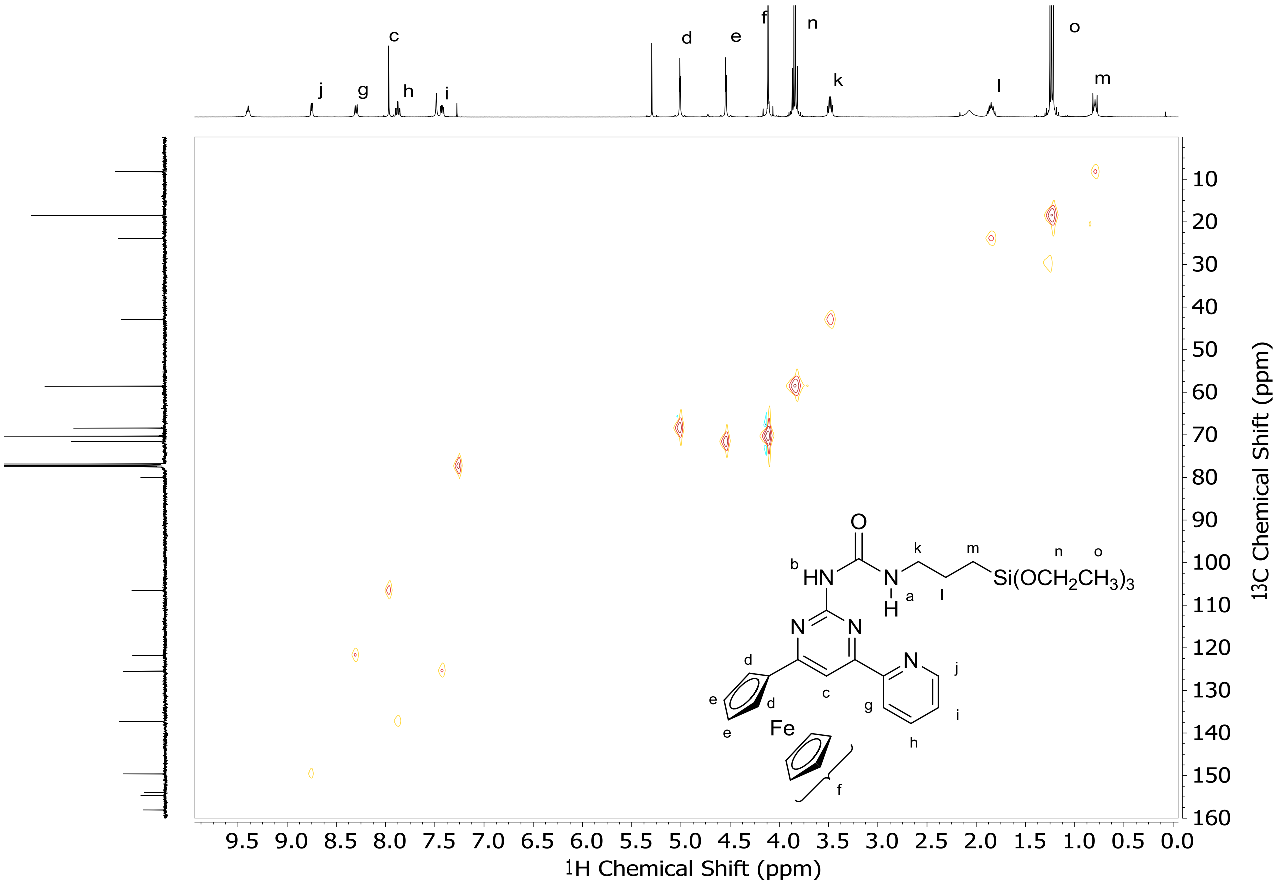

^{1}H–^{13}C HSQC spectrum of compound **9b** ($CDCl_3$, 295 K, 400 MHz).

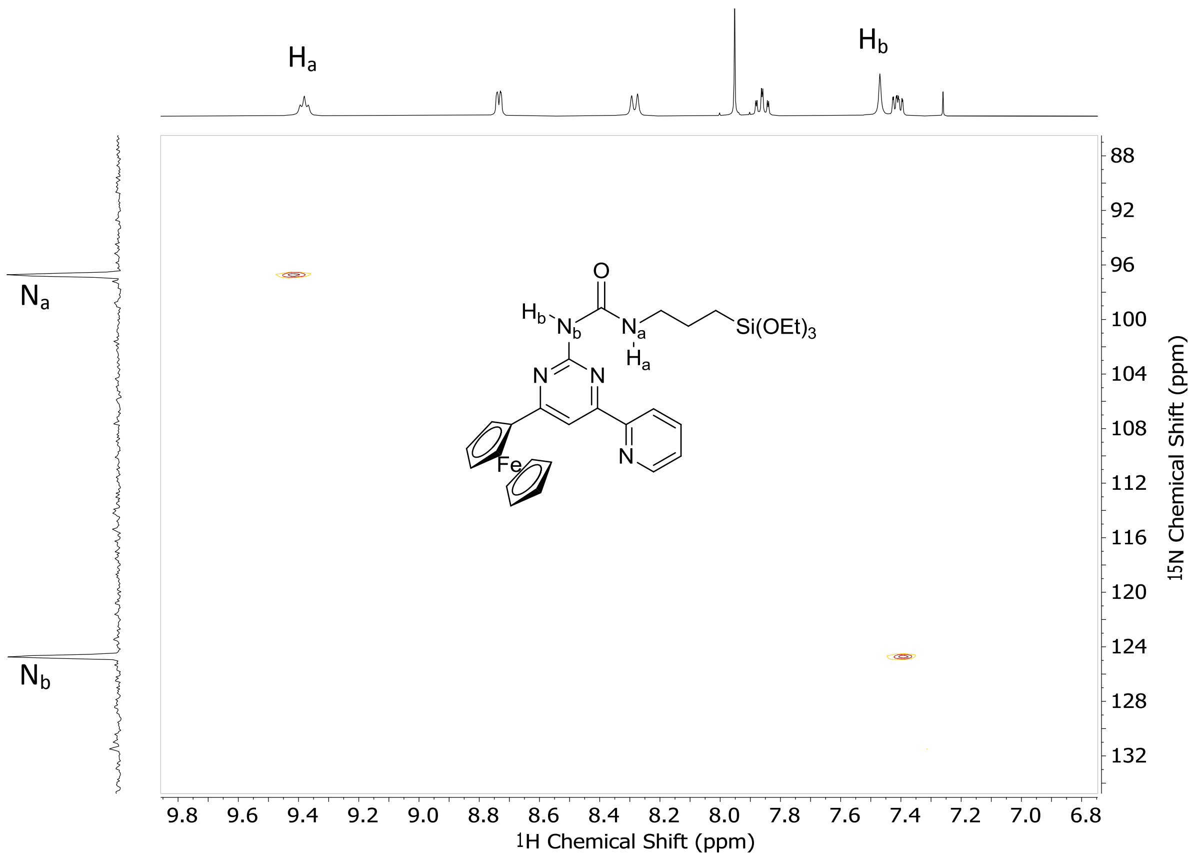

^{1}H–^{15}N HSQC spectrum of compound **9b** ($CDCl_3$, 298 K, 400 MHz).

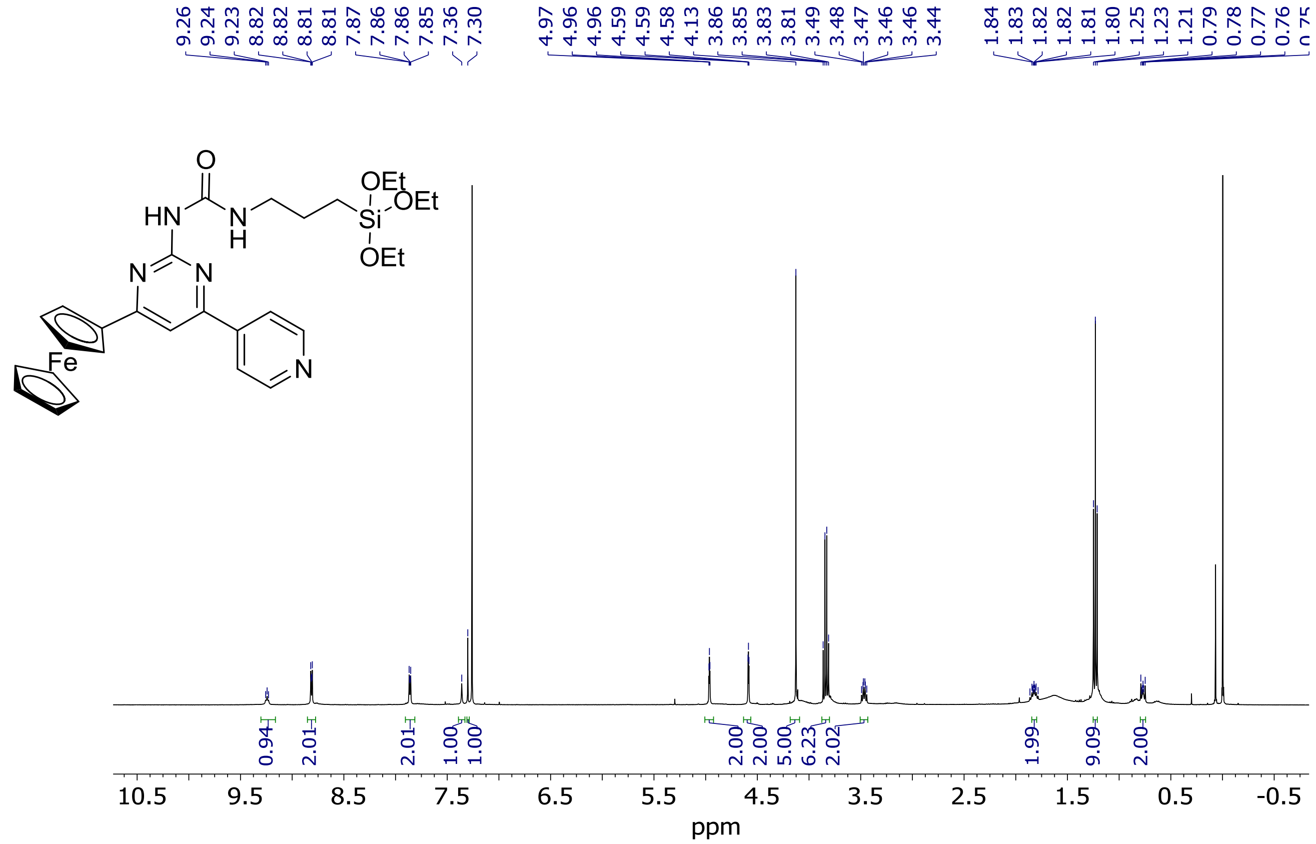

^{1}H NMR spectrum of compound **9c** ($CDCl_3$, 400.13 MHz).

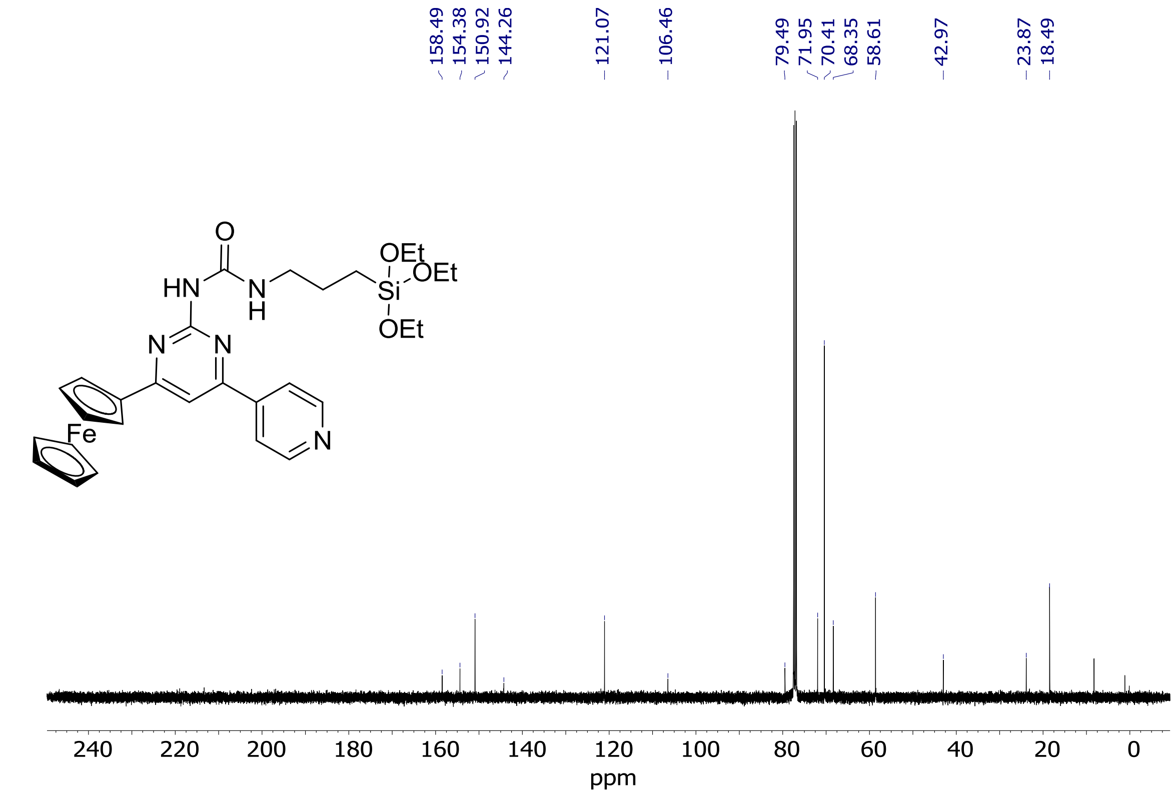

$^{13}C\{^1H\}$ NMR spectrum of compound **9c** ($CDCl_3$, 100.62 MHz).

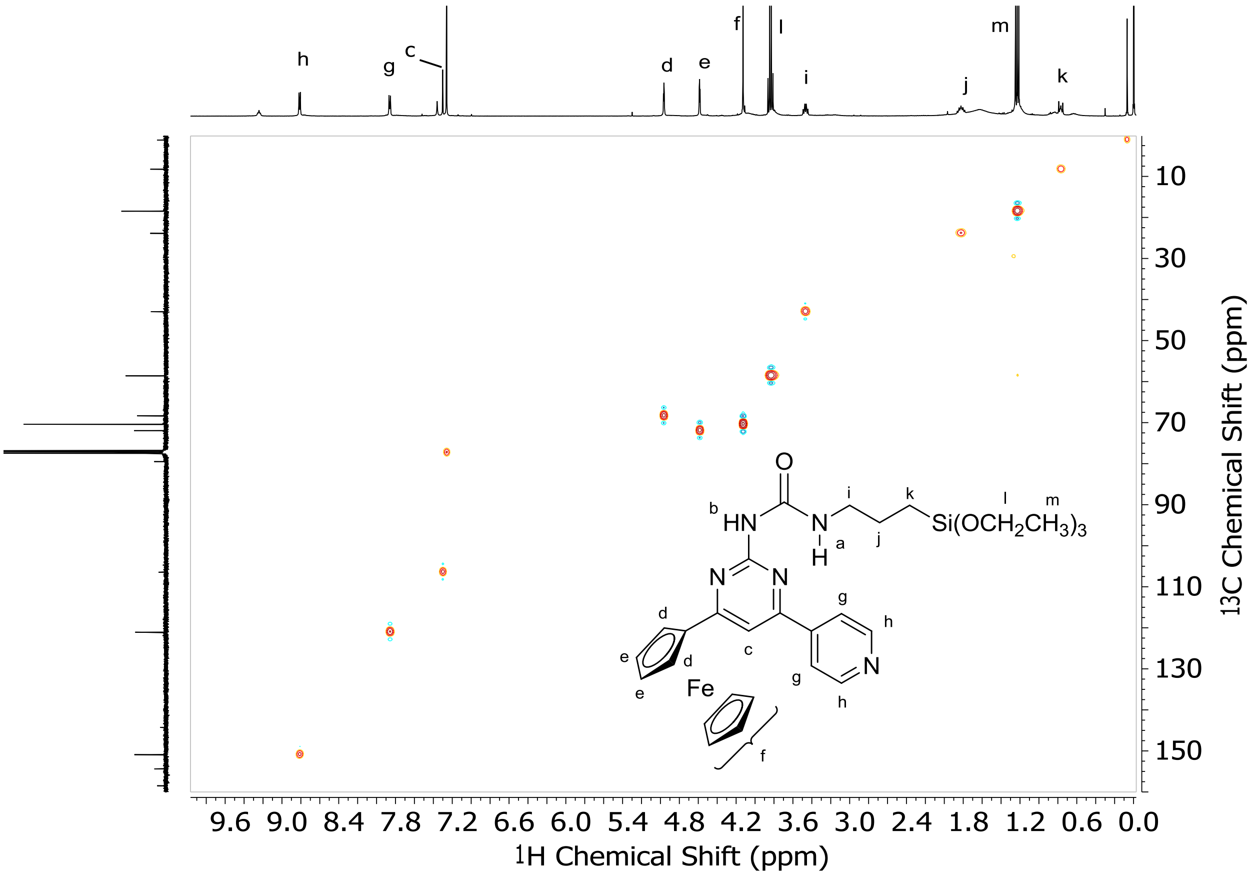

^1H–^{13}C HSQC spectrum of compound **9c** ($CDCl_3$, 298 K, 400 MHz).

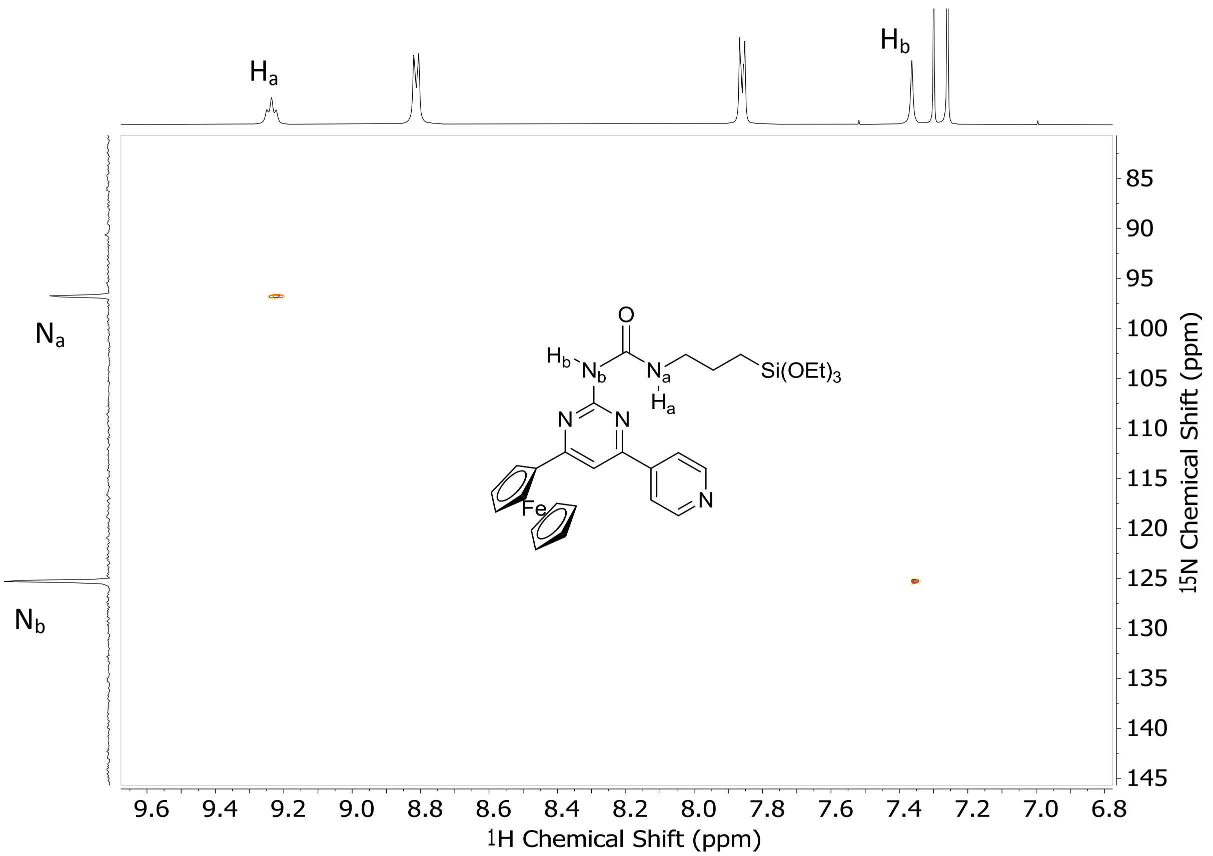

^{1}H–^{15}N HSQC spectrum of compound **9c** ($CDCl_3$, 298 K, 400 MHz).

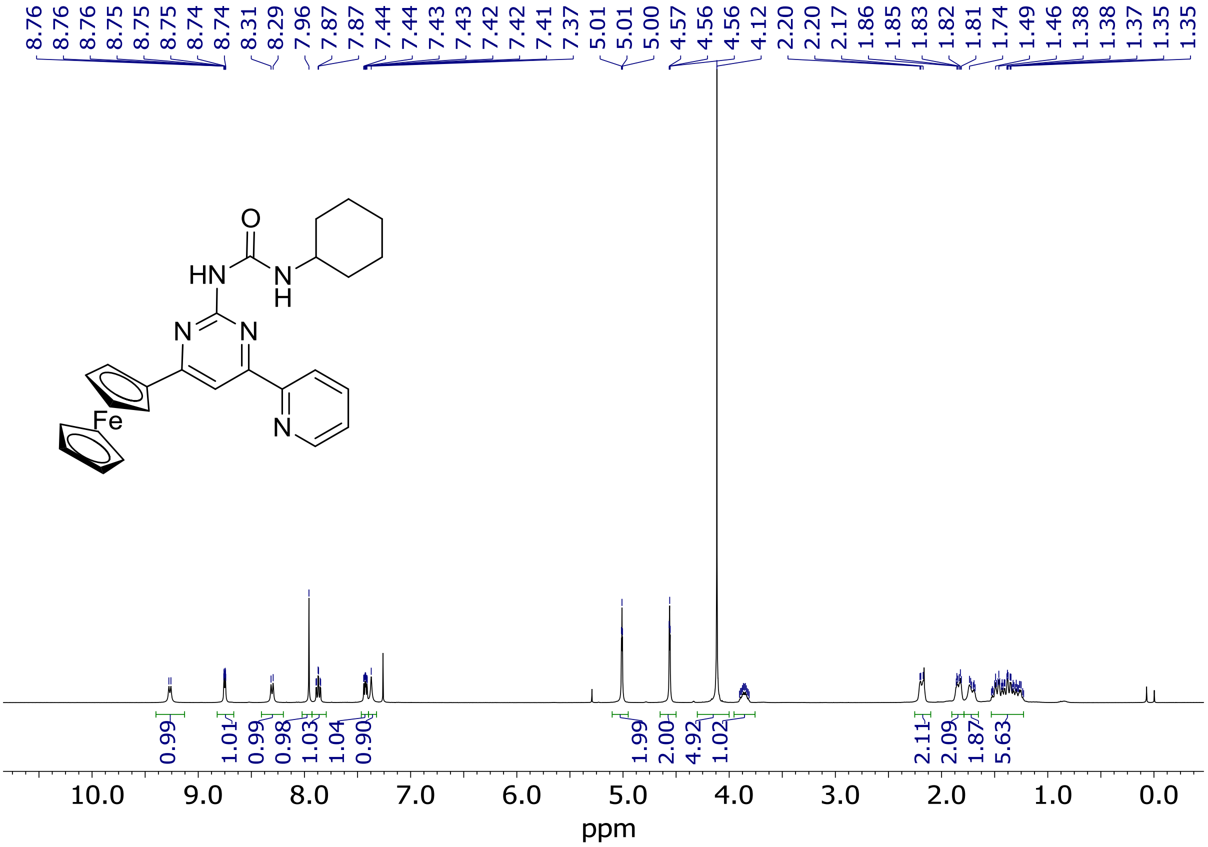

^{1}H NMR spectrum of compound **10** ($CDCl_3$, 400.13 MHz).

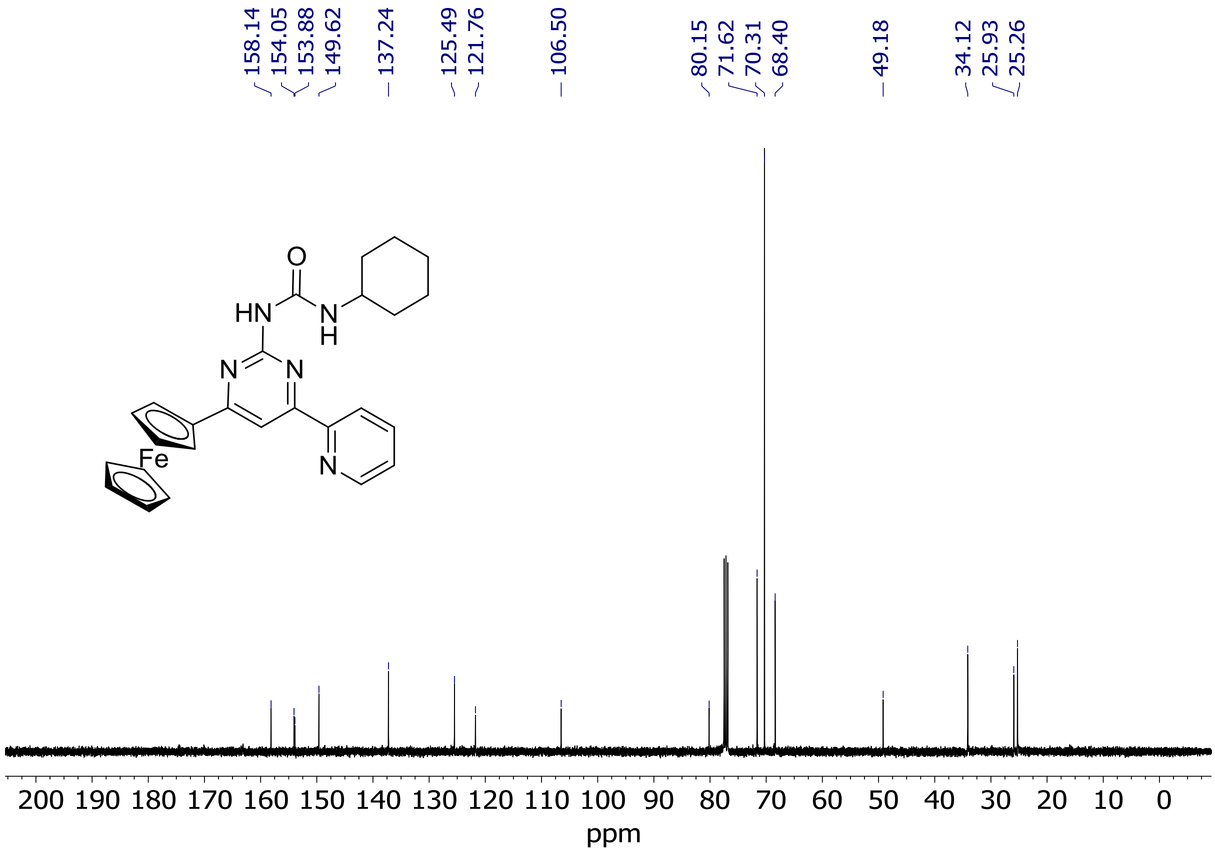

$^{13}C\{^1H\}$ NMR spectrum of compound **10** ($CDCl_3$, 100.62 MHz).

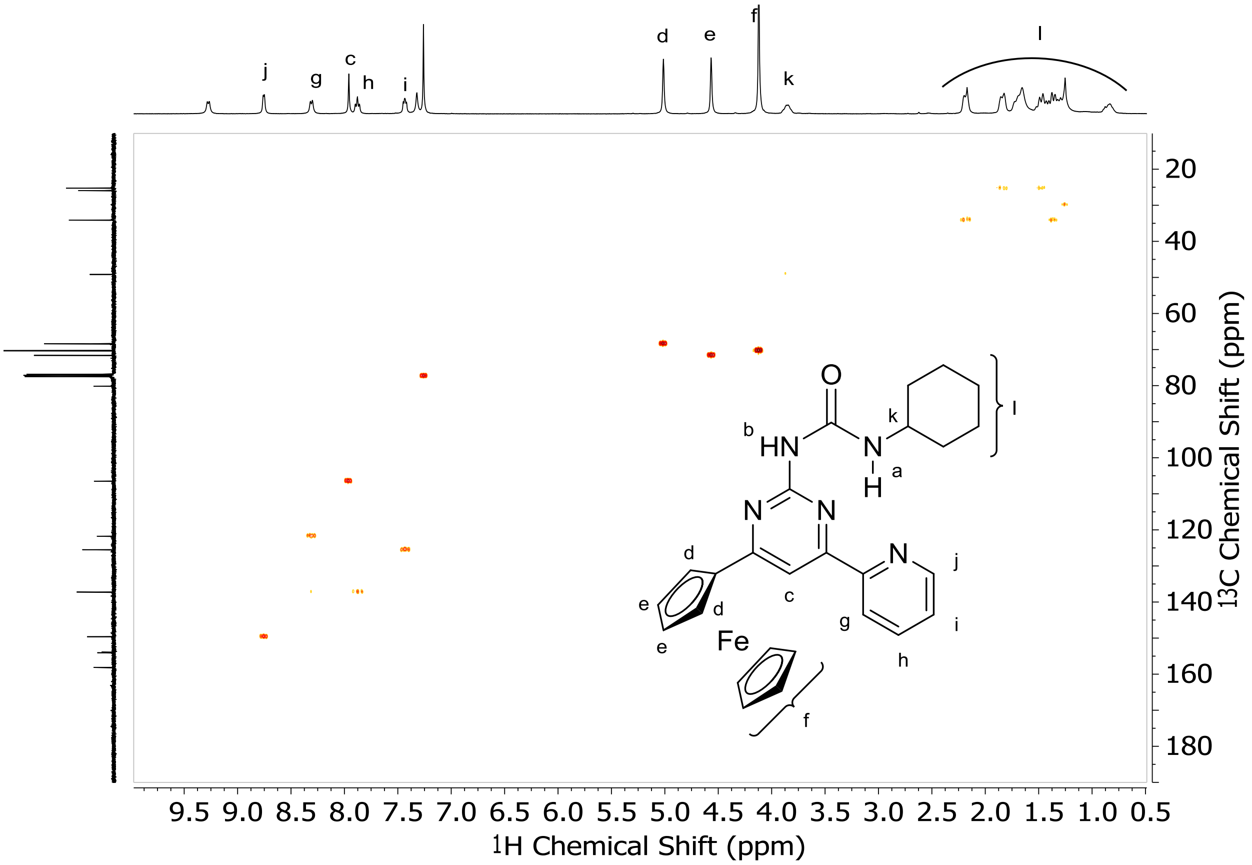

^1H–^{13}C HSQC spectrum of compound **10** ($CDCl_3$, 295 K, 400 MHz).
